# BeatBox—HPC simulation environment for biophysically and anatomically realistic cardiac electrophysiology

**DOI:** 10.1371/journal.pone.0172292

**Published:** 2017-05-03

**Authors:** Mario Antonioletti, Vadim N. Biktashev, Adrian Jackson, Sanjay R. Kharche, Tomas Stary, Irina V. Biktasheva

**Affiliations:** 1 EPCC, The University of Edinburgh, Edinburgh, United Kingdom; 2 CEMPS, University of Exeter, Exeter, United Kingdom; 3 EPSRC Centre for Predictive Modelling in Healthcare, University of Exeter, Exeter, United Kingdom; 4 Institute of Cardiovascular Sciences, School of Medical Sciences, University of Manchester, Manchester, United Kingdom; 5 Dept of Computer Science, University of Liverpool, Liverpool, United Kingdom; Georgia State University, UNITED STATES

## Abstract

The BeatBox simulation environment combines flexible script language user interface with the robust computational tools, in order to setup cardiac electrophysiology in-silico experiments without re-coding at low-level, so that cell excitation, tissue/anatomy models, stimulation protocols may be included into a BeatBox script, and simulation run either sequentially or in parallel (MPI) without re-compilation. BeatBox is a free software written in C language to be run on a Unix-based platform. It provides the whole spectrum of multi scale tissue modelling from 0-dimensional individual cell simulation, 1-dimensional fibre, 2-dimensional sheet and 3-dimensional slab of tissue, up to anatomically realistic whole heart simulations, with run time measurements including cardiac re-entry tip/filament tracing, ECG, local/global samples of any variables, etc. BeatBox solvers, cell, and tissue/anatomy models repositories are extended via robust and flexible interfaces, thus providing an open framework for new developments in the field. In this paper we give an overview of the BeatBox current state, together with a description of the main computational methods and MPI parallelisation approaches.

## Introduction

### Background

Cardiovascular disease (CVD) is the main cause of death in Europe, accounting for 47% of all deaths [1]. Cardiac arrhythmias, where the electrical activity of the heart responsible for its pumping action is disturbed, are among the most serious CVDs. Despite over a century of study, the circumstances from which such fatal cardiac arrhythmias arise are still poorly understood. Although several advancements have been made in linking genetic mutations to arrhythmogenic CVD [2–4], these do not explain the resultant mechanisms by which arrhythmia and fibrillation emerge and sustain at the whole heart level, for the position of the heart in torso makes *in vivo* measurement awkward and invasive, prohibitively so for study in humans. Thus, for some genetic cardiac diseases, the first presenting symptom is death with understandably limited opportunity to make even superficial examinations *in vivo*. The most modern experimental methods do not provide sufficient temporal and spatial resolution to trace down the multi-scale fine details of fibrillation development in samples of cardiac tissue, not to mention the heart in vivo.

Combination of mathematical modelling and the latest realistic computer simulations of electrical activity in the heart have much advanced our understanding of heart fibrillation and sudden cardiac death [5, 6], and the impact of *in-silico* modelling, or indeed in-silico “testing”, is expected to increase significantly as we approach the ultimate goal of the whole-heart modelling. With the vast amount of quantitative experimental data on cardiac myocytes action potential and the underlying transmembrane ionic currents ready for inclusion into the in-silico modeling, and the recent advance in high-resolution DT-MRI provision of detail anatomy models, the biophysically and anatomically realistic computer simulations allow unimpeded access to the whole heart with greater spatial and temporal resolutions than in a wet experiment, and allow to synthesise such elusive phenomena for closer study, hence improving prospects of their treatment and prevention.

The biophysically and anatomically realistic simulation of cardiac action potential propagation through the heart is computationally expensive due to the huge number of equations per cell and the vast spatial and temporal scales required. Complexity of realistic cardiac simulations spans multi-physical scales to include greater detail at cellular level, tissue heterogeneity, complex geometry and anisotropy of the heart. Due to huge number of strongly nonlinear equations to be solved on the vast temporal and spatial scales determined by the high-resolution DT-MRI anatomy models, its timely running relies on use of parallel processors—High Performance Computing (HPC).

**Fig 1:**
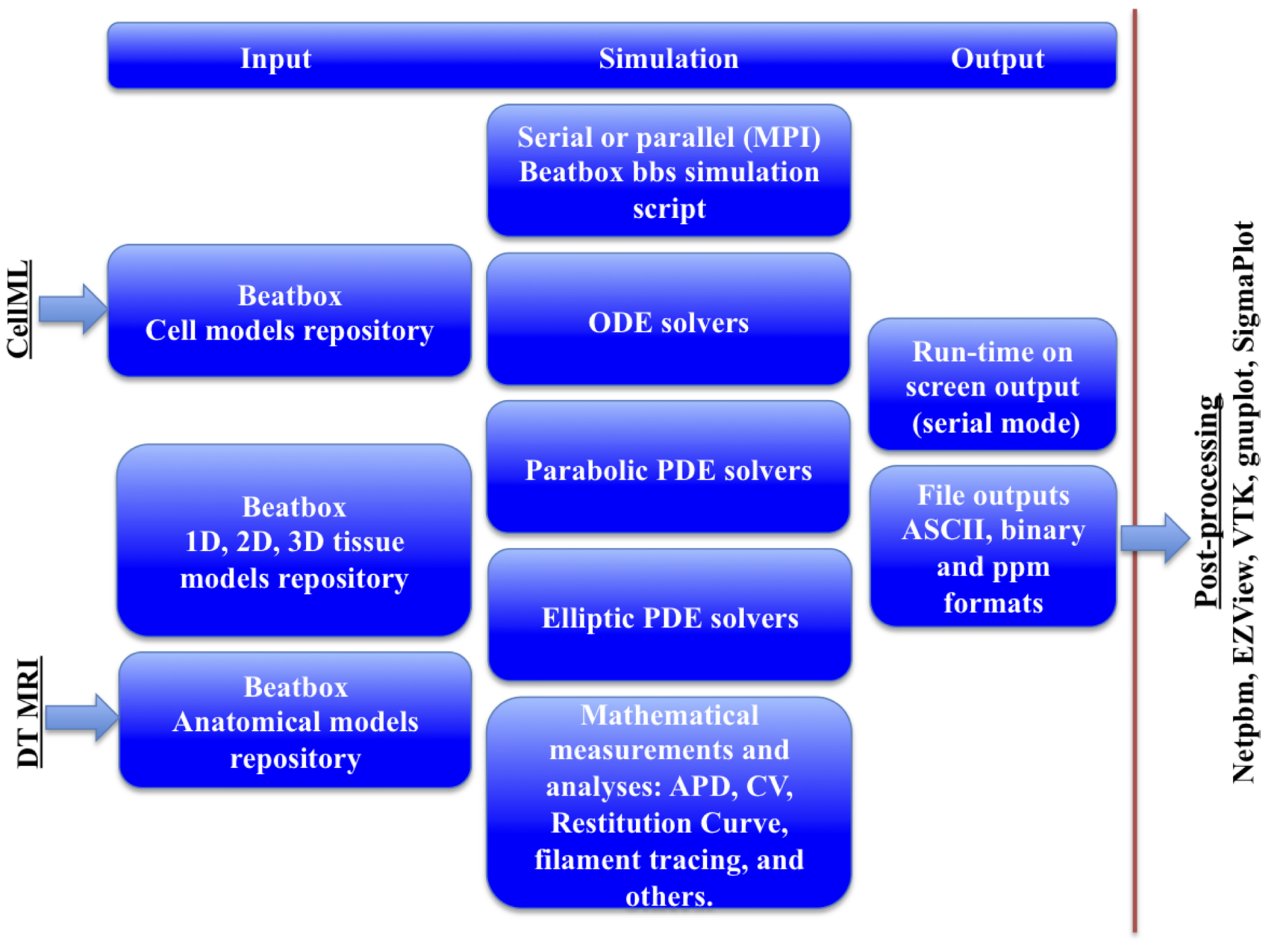
BeatBox formalism paradigm [7].

To address the intrinsically modular cardiac electrophysiology in silico modelling, we developed modular software package BeatBox ([7, 8]; the early stages of development of BeatBox and its predecessor QUI benefited from contributions by A.V. Karpov and R. McFarlane; Karpov provided the portable compiler of arithmetic expressions for QUI; McFarlane contributed to MPI parallelization of QUI, and treatment of complex geometries, and is the author of its new name, BeatBox), with a built-in simulation script interpreter, extendable repositories of cell and tissue/anatomy models, capable to run both sequentially and in parallel on distributed (MPI) memory architecture, Fig 1. The Beatbox cardiac simulation environment allows setup of complicated numerical experiments without re-coding at low-level, so that cell excitation, tissue and anatomy models, stimulation protocols may be included into a script, and BeatBox simulation run either sequentially or in parallel without re-compilation. Importantly, the BeatBox modular paradigm provides an open framework for new developments in the field, for the open source BeatBox solvers, and cell and tissue/anatomy repositories are extended via robust and flexible interfaces.

As HPC is a specialist field in its own right, it normally demands a high qualification of the end users if they were to modify an existing code for a different application problem. The main idea of BeatBox is to make use of MPI routinely acessible to a widest community of users vast majority of which are not professional software developers, therefore BeatBox’s MPI implementation aims to stay opaque to the user.

A number of successful computational cardiology applications simulating electrophysiology and/or biomechanics are available, e.g. CARP [9], CHASTE [10], Continuity [11], CMISS [12], Myokit [13], CVRG Galaxy [14]; further reviews and benchmark comparisons can found in [15] and [16]. Most of the available software is taylored for solution of limited particular aspects of cardiac simulation. A typical set-up allowed by electrophysiology simulation software is a certain number of electrical stimuli applied at certain times in certain ways, and the user is allowed to vary the number and parameters of these stimuli. Anything more complicated would require re-coding at low level. As we show on particular examples in this paper, the capabilities of BeatBox workflow scripts are much wider. Another important feature is that BeatBox finite difference implementation allows straightforward incorporation of Cartesian DT-MRI and/or micro-CT data into the cardiac electrophysiology simulations. Majority of the popular cardiac simulation software, including [9–12], use finite element discretization. Conversion of anatomical data into finite element meshes is a separate step, requiring specialized tools, and can be a research task in itself [17].

We believe that the main achievement of BeatBox modular and scripting approach is that it allows, on one hand, to maintain user flexibility for a large variety of simulation tasks, while on the other hand, to relieve the user from necessity of going into the code low level neither for changing the simulation protocol, nor for parallelisation detail. As the scripts contain no data specific to their use in parallel, the same scripts can run equivalently in both sequential and parallel modes (with the exception of run-time visualization). In the subsequent sections, we outline the mathematical problems, numerical methods and programming approaches characterising BeatBox.

### Cardiac tissue models

Computer simulation of cardiac muscle requires a mathematical model, describing the relevant biophysical and electrophysiological processes. The *bidomain* model considers intracellular and extracellular spaces in the syncytium of cardiac myocytes. Those two domains are separated from each other by cellular membranes, the conductivity through which is controlled by ionic channels. This situation is described by a system of partial (PDE) and ordinary (ODE) differential equations of the form:

$$\begin{array}{c} C_{m}\frac{\partial V}{\partial t}=-I_{\text{ion}}\left(V,g\right)+\frac{1}{\chi }\nabla \cdot {\hat{\sigma }}_{i}\nabla \Phi_{i},\\ \nabla \cdot \left({\hat{\sigma }}_{i}+{\hat{\sigma }}_{e}\right)\nabla \Phi_{i}=\nabla \cdot {\hat{\sigma }}_{e}\nabla V-I_{\text{ext}}\\ \frac{\partial g}{\partial t}=f\left(g,V,\vec{r}\right), \end{array}$$ (1)

where *V* is the transmembrane voltage, Φ_*i*_ is the intracellular electrostatic potential (so Φ_*e*_ = Φ_*i*_ − *V* is the extracellular potential), ${\hat{\sigma }}_{i}$ and ${\hat{\sigma }}_{e}$ are the anisotropic conductance tensors of the intra- and extracellular domains respectively, *C*_*m*_ is the specific capacitance of the membrane and *χ* is the average surface to volume ratio of the cells. The transmembrane ionic currents *I*_ion_ are controlled by gating variables and ionic concentrations, represented by the vector **g**. The kinetic rates are expressed in terms of the vector-function **f**. The term *I*_ext_ designates the external elecric current, say from experimental or defibrillation electrodes. In the system (1), the first equation is parabolic, the second is elliptic and the third effectively is a system of ODEs at every point of the tissue characterised by its location $\vec{r}$. If the intracellular conductances are proportional, i.e. ${\hat{\sigma }}_{e}=\nu {\hat{\sigma }}_{i}$ for a scalar *ν*, then Φ_*i*_, Φ_*e*_ and *V* are proportional to each other, and the system (1) simplifies to a *monodomain* model:

$$\begin{array}{c} C_{m}\frac{\partial V}{\partial t}=-I_{\text{ion}}\left(V,g\right)+\frac{1}{\chi }\nabla \cdot {\hat{\sigma }}_{\text{eff}}\nabla V-I_{\text{eff}}\left(\vec{r},t\right),\\ \frac{\partial g}{\partial t}=f\left(g,V,\vec{r}\right), \end{array}$$ (2)

where ${\hat{\sigma }}_{\text{eff}}=\frac{\nu }{1+\nu }{\hat{\sigma }}_{i}$, $I_{\text{eff}}=\frac{1}{\chi (1+\nu )}I_{\text{ext}}$. System (2) belongs to the class of *reaction-diffusion* systems, used for modelling of a large variety of natural and artificial nonlinear dissipative systems [18].

Computationally, the bidomain description is dramatically more challenging than the monodomain, as the elliptic equation has to be solved at every time step (see e.g. [19]). Practice shows that unless an external electric field is involved, the bidomain models give results that differ only slightly from corresponding appropriately chosen monodomain models [5, 20–23], which, together with the fact that experimental data on the intra- and extracellular conductivity tensors are scarse, means that in practice the monodomain simulations are used more widely.

The complexity of cardiac electrophysiology simulations further increases as it spans multiple physical scales to include greater detail at the cellular level, such as cell signalling and metabolism, and greater integration with the surrounding biological systems, such as electromechanical coupling and vascular fluid dynamics [6, 24–28]. In this context, it is not surprising that the timely completion of simulations relies on modern high performance computing hardware.

Use of HPC facilities, although essential, is severely limited by specialized software development skills required, so a separation of the low-level coding from the processes of formulating and solving research problems is highly desirable. The BeatBox project seeks to overcome these difficulties by providing a computational environment that could serve as a unifying paradigm for all *in silico* cardiac electrophysiology research, and for research in similar phenomena involving reaction-diffusion systems outside the cardiology domain.

## Design, computational algorithms, and implementation

### Logical structure and user interface

**Fig 2:**
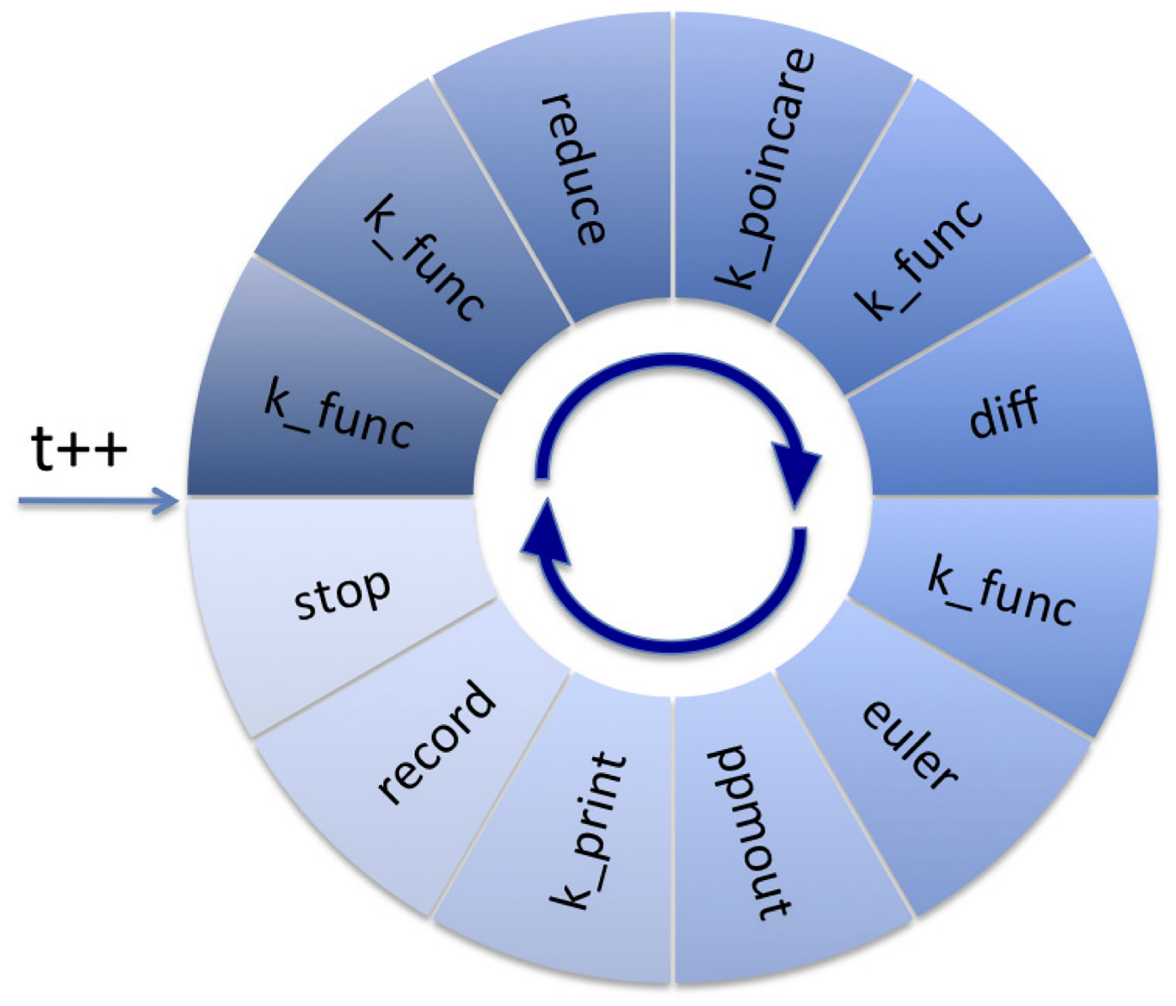
BeatBox “Ring of devices”. The ring of devices set up by sample.bbs script (see Listing 2 in the Appendix).

The fundamental paradigm used by BeatBox is to represent a simulation as a ring of “devices”, i.e. individual modules that perform specific computational, input/output or control tasks. This ring is a metaphor of an iteration cycle; typically, one time step of calculations corresponds to one turn around the ring (see Fig 2). Module of each type can be used more than once in the ring, thus providing more than one device instance. This ring of devices is constructed at start-up, based on the instructions given in an input script. The BeatBox script parser places devices into the ring in the same order as they appear in the script. The script describes the sequence of devices used in a particular simulation and their parameters, using a domain-specfic scripting language with a flexible syntax that includes things like a built-in interpreter of arithmetic expression, recursive calls to other scripts, etc. This allows **complicated numerical experiments to be set-up without low-level re-coding**.

With some simplification, BeatBox computable data are of two kinds: the main bulk of the data is in a four-dimensional computational grid, of which three dimensions correspond to the spatial dimensions, and the fourth dimension enumerates *layers* allocated for the components of the reaction-diffusion system, Eqs (1) or (2), as well as for the output data produced by some of the devices (e.g. those computing the values of the diffusion term), or as a scratch memory area for devices that require this. When working with a complicated geometry, only a subset of the regular cuboid spatial 2D or 3D grid corresponding to the tissue points is involved. Every device typically works with only some layers of the grid. Apart from the computational grid, there are global variables, values of which may be used by some devices, and modified by others. From the MPI viewpoint, the main difference between the two types of data is that the computational grid is divided between threads, i.e. each thread has its own portion of the data, whereas the global variables are shared, i.e. each thread has the same set of global variables. The values of the global variables are identical in all threads; this is ensured either as they are produced as a result of identical computations in each thread, or, if a global variable is computed in one thread, its value is broadcast to other threads. An important feature is that any device instance in the ring is associated with a global variable that serves as the flag indicating whether this device instance should be executed at this particular time step iteration in the computation.

All MPI parallelization is done within individual devices, so conceptually, the device ring functions synchronously in all threads, with actual synchronising MPI_Barrier or MPI_Sendrecv calls done only when required by the computation flow. Some devices operate on a sub-grid of the 4D data grid (restricted “space”), so that the set of active threads may change from device to device, and from one instance of a device to the other; if a device requires exchange of data between threads, then each device instance creates its own MPI communicator.

The MPI implementation of the script parsing does not present any significant difficulties. The script file is read by all threads, and the threads allocate relevant subgrids of the four-dimensional computational grid, corresponding to their subdomains (see Section “Complex geometries and domain partitioning” for detail), to every device they create. Some care is required for diagnostic output, e.g. normally BeatBox echoes the parsed version of the script to the log file. For normal output, this function is delegated to one thread; for error messages, the thread that detected the error will report it, which in case of massive parallelism may result in a very large log file if the error occurs in many threads.

Two simple examples of BeatBox scripts are provided in the Appendix. One of them is “minimalist”, corresponding to a simulation protocol, that can be done by many other cardiac simulation softare. The second script is slightly more complicated, in order to illustrate the BeatBox specific features on an example of a simulation of the feedback-controlled resonant drift algorithm as described in [29], with Fig 2 illustrating the corresponding ring of the devices. This script involves the emulation of an electrode registering electrical activity of the cardiac muscle at a point, and of an external device which switches on a time-delay line when a signal from the registering electrode satisfies a certain condition, and issues a low-voltage defibrillating shock upon the expiration of the time delay. We stress here that implementation of this protocol in the majority of other cardiac simulation software would require modifications of the code at a low level.

We believe that the main features of BeatBox are the flexibility of its user interface, and the fact that any new computational features can always be added as a new device. At the same time, it is clear that its utility at present depends on specific computational capabilities. In the next sections, we describe the BeatBox components most likely to be required in a typical cardiac dynamics simulations, in their current state. In the main text of the paper we focus on principal utility features of BeatBox; for a more detailed description of the syntax and semantics of BeatBox scripts, the reader is referred to the Appendix, whereas a fully comprehensive description is, of course, to be found in the user manual [30] which is distributed with the software and is not part of this communication.

### Splitting the problem into parts

#### Computation of intermediate expressions

“Divide and conquer” is a popular and successful strategy for evolution-type problems. The idea is to split the right-hand sides of complex evolution equations to simpler components, implement solvers corresponding to each of these components, and then coordinate the work of the solvers, so together they solve the whole problem. The modular structure of a BeatBox job makes this approach particularly easy to implement. One way of doing so is by computing different parts of the right-hand side by different devices, and then allow the time-stepping device to use the results of these computations. We illustrate this by using a simple example, with reference to the script sample.bbs presented in the Listing 2 in the Appendix and illustrated in Fig 2. Ignoring the effect of an external electric field for now, the mathematical problem solved by this script is:

$$\begin{array}{c} \frac{\partial u}{\partial t}=\frac{1}{\epsilon }\left(u-u^{2}/3-v\right)+\nabla \hat{D}\nabla u,\\ \frac{\partial v}{\partial t}=\epsilon \left(u+\beta -\gamma v\right). \end{array}$$ (3)

The script implements a forward Euler timestep (see Section “Explicit solvers” below) for system (3), using two devices:

**Table:** 

diff v0 = [u] v1 = [i] Dpar = D Dtrans = D/4 hx = hx;
euler v0 = [u] v1 = [v] ht = ht ode = fhncub
par = {eps = eps bet = @[b] gam = gam Iu = @[i]};

According to the definitions of string macros in the script, Listing 2, the macro [u] in this fragment expands to 0, that is the very first layer of the grid allocated to the *u* field, [v] expands 1, standing for the second layer of the grid, allocated to the *v* field, and [i] expands to 2, which is the third layer of the grid, for the value of the diffusion term in Eq (3), $\nabla \cdot \hat{D}\nabla u=\chi^{-1}\nabla \cdot {\hat{\sigma }}_{\text{eff}}\nabla u$. So, the diff device computes an auxiliary variable

$${\left(I_{u}\right)}_{n}=\nabla \cdot \hat{D}\nabla u_{n}$$

where *u*_*n*_ stands for the *u* field at the current time step *n*, and stores it into layer [v] = 2 of the grid.

The euler device, with the parameter ode = fhncub, performs a forward Euler step for the cubic FitzHugh-Nagumo ODE system,

$$\begin{array}{c} \frac{\partial u}{\partial t}=\frac{1}{\epsilon }\left(u-u^{2}/3-v\right)+I_{u},\\ \frac{\partial v}{\partial t}=\epsilon \left(u+\beta -\gamma v\right)+I_{v}, \end{array}$$ (4)

in which parameters *ϵ* and *γ* are given by the values of the global variables eps and gam defined previously in the script (in the included parameter file <fhn.par>), the value of parameter *β* is taken from layer [b] which expands to 3 (parameter *β* is spatially dependent in this simulation, and layer 3 was pre-filled with values by the same k_func device that computed the initial conditions), the value of parameter *I*_*u*_ is taken from layer [i] which contains the values of the anisotropic diffusion term (*I*_*u*_)_*n*_, computed for this time step by the preceding diff device, and the value of parameter *I*_*v*_ = 0 by default. Overall, with *u*_*n*_(*x*, *y*) and *v*_*n*_(*x*, *y*) designating the fields *u* and *v* at the *n*-th time step, the pair of devices computes

$$\begin{array}{l} u_{n+1}=u_{n}+k\left[\frac{1}{\epsilon }\left(u_{n}-\frac{1}{3}u_{n}^{2}-v_{n}\right)+\nabla \cdot \hat{D}\nabla u_{n}\right],\\ v_{n+1}=v_{n}+k\epsilon \left(u_{n}+\beta \left(x,y\right)-\gamma v_{n}\right), \end{array}$$

where *k* is the time step, represented by the global variable ht in the BeatBox script.

#### Operator splitting

Operator splitting is another popular “divide and conquer” strategy [5, 31, 32]. Slightly simplifying, one can say that in this approach, the right-hand sides still are split into simpler parts, but now an evolution sub-step is done for each such part in turn, as if this part was the whole right-hand side. For example, computation of kinetics and diffusion in the right-hand side of Eq (3) can be split into the kinetics part and diffusion part, and then one device performs the diffusion substep, and another device performs the kinetics substep. So, the BeatBox script fragment from Section “Computation of intermediate expressions” can be modified as

**Table:** 

diffstep v0 = [u] v1 = [i] Dpar = D Dtrans = D/4 hx = hx ht = ht;
euler v0 = [u] v1 = [v] ht = ht ode = fhncub
par = {eps = eps bet = @[b] gam = gam};

where the device diffstep computes the diffusion term, and does a forward Euler step with it, as if this was the only term in the equation. Combined with the fact that now in the euler device the parameter Iu is not specified so it defaults to zero, the given fragment of the script implements the following computation scheme:

$$\begin{array}{l} u_{n+1/2}=u_{n}+k\left[\nabla \cdot \hat{D}\nabla u_{n}\right],\\ u_{n+1}=u_{n+1/2}+k\left[\frac{1}{\epsilon }\left(u_{n+1/2}-\frac{1}{3}u_{n+1/2}^{2}-v_{n}\right)\right],\\ v_{n+1}=v_{n}+k\epsilon \left(u_{n+1/2}+\beta \left(x,y\right)-\gamma v_{n}\right), \end{array}$$

Once again, this is just a simple example illustrating how the BeatBox paradigm naturally fits the idea of operator splitting. This of course applies first of all to the simplest (Lie) splitting; more sophisticated, higher-order operator splitting schemes could be implemented at the BeatBox script level, or on the device, i.e. a C-source code level.

From the MPI viewpoint, both methods of splitting problems into parts do not present any issues, since they are implemented on the level of interaction of devices involved, and any parallelization work is done within the devices.

### Kinetics solvers

#### Explicit solvers

Both the monodomain “reaction-diffusion” models of the form Eq (2) or the more complicated bidomain model (1) have equations with time derivatives. Solving those equations in BeatBox is done as if they were ordinary differential equations,

$$\begin{array}{c} \frac{dV}{dt}=-\frac{1}{C_{m}}\left(I_{\text{ion}}\left(V,g\right)-I_{\text{eff}}\left(\vec{r},t\right)\right)\\ \frac{dg}{dt}=f\left(g,V,\vec{r}\right), \end{array}$$ (5)

(depending on $\vec{r}$ as a parameter) either with the value of the diffusion term, computed by the corresponding diffusion device, appearing in the voltage equation, or within the operator-splitting paradigm, i.e. performing time sub-steps as if the model was restricted to the ODEs representing the reaction terms, leaving the space-dependent part of the model to be computed at alternative sub-steps.

The simplest and arguably most popular in practice solver for ODEs is the first-order explicit (time-forward) scheme known as the **forward Euler** scheme, which for a system of ODEs Eq (5) means:

$$\begin{array}{c} V_{n+1}=V_{n}-\frac{k}{C_{m}}\left(I_{\text{ion}}\left(V_{n},g_{n}\right)-I_{\text{eff}}\left(\vec{r},t_{n}\right)\right)\\ g_{n+1}=g_{n}+kf\left(g_{n},V_{n},\vec{r}\right), \end{array}$$ (6)

where *t*_*n*_ is the *n*-th value in the time grid, *k* = *t*_*n*+1_ − *t*_*n*_ is the time step, and $V_{n}=V\left(\vec{r},t_{n}\right)$, $g_{n}=g\left(\vec{r},t_{n}\right)$. This scheme is implemented in BeatBox in the euler device.

The Euler scheme’s well known disadvantages are its low accuracy due to only first-order approximation of the ODE, and, as any explicit scheme, only conditional stability (see e.g. [33]). The first disadvantage does not usually play a crucial role in cardiac dynamics studies as the proven accuracy of cardiac kinetics models themselves is not particularly high. There is, however, rk4 device in BeatBox, implementing **Runge-Kutta fourth-order scheme** for cases when accuracy is essential, and other standard explicit solvers may be easily implemented in a similar way. The stability consideration is more significant as it severely limits the maximal allowable time step *k* in stiff models, hence making simulations costly.

#### Exponential solvers

The standard solution to the stability problem is, of course, using implicit or semi-implicit schemes. The latter possibility is much more popular as fully implicit approaches for nonlinear equations are numerically challenging. Among the semi-implicit approaches available in cardiac dynamics, the exponential scheme for ionic gates, known as the **Rush-Larsen technique** [34], is very popular. The idea is based on the observation that in the models of ionic excitability, since the seminal work by Hodgkin and Huxley [35], an important role is played by equations of the form:

$$\frac{dy}{dt}=\alpha \left(V\right)\left(1-y\right)-\beta \left(V\right)y,$$ (7)

where the dynamic variable *y*, called the *gating variable*, possibly in conjunction with other gating variables, determines the permittivity of certain ionic currents. A convenient (even if not biophysically precise) interpretation is that a channel is open if all of the gates controlling that channel are open, and the variable *y* is the probability for that gate to be open. Hence *α* and *β* are transition probabilities per unit of time, of a closed gate to open, or for an open gate to close, respectively. In Eq (7) the transition probabilities depend on the current value of the transmembrane voltage *V*, as in the Hodgkin-Huxley model; in more modern models gating variables of some channels may depend on other dynamical variables, say the concentration of calcium ions. The importance of the gating variables is that equations of the type Eq (7) are often the stiffest in the whole cardiac excitation model. The Rush-Larsen scheme in its simplest form can be obtained by assuming that *V* does not change much during a time step, *t* ∈ [*t*_*n*_, *t*_*n*+1_], and replacing *V*(*t*) with the constant value *V*_*n*_ = *V*(*t*_*n*_) turns Eq (7) into a linear equation with constant coefficients, the solution of which can be written in a closed form, which gives:

$$y_{n+1}=A\left(V_{n}\right)+B\left(V_{n}\right)y_{n}$$ (8)

where

$$\begin{array}{c} A\left(V\right)=\exp\left(-(\alpha (V)+\beta (V))k\right),\\ B\left(V\right)=\frac{\alpha (V)}{\alpha (V)+\beta (V)}\left[1-A(V)\right]. \end{array}$$ (9)

As far as Eq (7) is concerned, this scheme is unconditionally stable, and gives an exact answer if *V*(*t*) = *const*, i.e. its first-order accuracy depends exclusively on the speed of change of the transmembrane voltage *V*. This scheme is implemented in the BeatBox device rushlarsen. Naturally, this device requires a more detailed description of the excitable model than euler: the gating variables *y* and their transition rates *A*, *B* need to be explicitly identified for rushlarsen whereas euler only requires a definition of the functions computing the right-hand sides of the dynamic equations, i.e. *I*_ion_ and **g**. The standard Rush-Larsen scheme can be modified to improve its accuracy; e.g [36] proposed a predictor-corrector version which provides a second order accuracy. Implementation of this method would require a description of the cellular model in the same ionic format as used by rushlarsen device; however it is not yet implemented in the current version of BeatBox.

Some modern cardiac excitation models use a Markov chain description of the ionic channels. This description is based on the assumption that an ionic channel can be in a finite number of discrete states, and transitions between the states can happen with certain probability per unit of time, which may depend on control variables, such as transmembrane voltage *V* or calcium ion concentration *c*. The time evolution of the vector **u** of the probabilities of the channel to be in each particular state is described by the system of linear ODEs, known in particular as *Kolmogorov (forward) equations*, or the *master equation*

$$\frac{du}{dt}=M\left(V,c\right)u,$$ (10)

where **M** is the matrix of transition rates. The extension of the Rush-Larsen idea to this system was done in [37]. Assuming again that the control variables do not change much within a time step and replacing them with a constant, *V*(*t*) = *V*_*n*_ and *c*(*t*) = *c*_*n*_ for *t* ∈ [*t*_*n*_, *t*_*n*+1_], the system (10) is a system of homogeneous linear equations with constant coefficients and its exact solutions can be explicitly written. Assuming that **M** is diagonalizable, the resulting computational scheme can be written as:

$$u_{n+1}=T\left(V_{n},c_{n}\right)u_{n},$$ (11)

where

$$T\left(V,c\right)=e^{kM(V,c)}=S\left(V,c\right)\;e^{\Lambda (V,c)k}\;S{\left(V,c\right)}^{-1}$$ (12)

and **S** and **Λ** are respectively the matrix of eigenvectors and the diagonal matrix of eigenvalues of **M**. This **matrix Rush-Larsen** scheme is also implemented in the device rushlarsen mentioned earlier.

Finding eigenvalues and eigenvectors for the diagonalisation and computing exponentials are relatively time consuming operations. For that reason the rushlarsen device does a *tabulation*. That is, for the case when the coefficients *A*, *B* depend on *V* and matrices **T** depend only on one control variable, e.g. *V* (are *“univariate”*), their values are precomputed for a sufficiently fine grid of the control variable at the start time. Tabulation is, of course, a very popular time-saving device, and is widely used in cardiac simulations, see e.g. [5, 9]. In BeatBox, tabulation is done automatically not only for transition rates, but for all univariate functions described as such in the ionic format of a cell model. In the rhs format there is no syntactic means for such detail so tabulation cannot be done automatically, but can of course be implemented in the C code describing the model.

If matrix **M** depends on multiple control variables, e.g. both *V* and *c* (are *“multivariate”*), it can sometimes be presented as a sum of univariate matrices. Then rushlarsen uses Lie operator splitting and integrates each of the subsystems associated with each of the univariate matrices using the tabulated “matrix Rush-Larsen” separately. For some kinetics models, **M** can be presented as a sum of one or more univariate matrices and a remainder, which is multivariate but uniformly small. In that case the subsystem associated with the small remainder is done using the forward Euler method. Finally, if any such decomposition is not possible, “matrix Rush-Larsen” step still can be done, just without tabulation, but by doing the diagonalization “on the fly”, i.e. at the run time rather than start time. Although such computation is relatively costly, the benefit of larger time step may still outweigh the expense. The possibility of tabulating multivariate function theoretically exists but is not considered in BeatBox due to resource implications.

The diagonalization is done using appropriate routines from GSL [38]; the relevant subset of GSL is included in the BeatBox distribution for portability and the users convenience.

Other methods of extending Rush-Larsen idea to Markov chains have been proposed; e.g. the “uniformization method” [39] based on computing partial Taylor series of the matrix exponential for the suitably preconditioned matrix **M**. This method does not require finding eigenvectors, but the amount of computations depends on the required accuracy.

From the MPI viewpoint, all kinetic solvers work on individual grid points, so parallelization does not present any issues.

#### Cell models

The current version of BeatBox is provided with a library of cell models. The definitions of the models come in two different formats, called rhs and ionic in BeatBox language, for the two different classes of kinetic solvers described above.

The rhs format is used by the generic solvers such as Euler and Runge-Kutta. In this format, the corresponding C module defines a function that computes the vector of the time-derivatives of the dynamic variables, for a given vector of the current values of those variables.

A practical amendment to this idealized scheme came from the necessity to incorporate models which are not easily presentable as systems of ordinary differential equations. This includes the models where the description of intracellular calcium buffers is in terms of finite rather than differential equations, and also the models with the description of calcium-induced calcium release in terms of a delay with respect to the voltage upstroke inflexion point, as in the Luo-Rudy family of models. The descriptions of such models used in cardiac modelling practice is often in the form of a function that performs the time-stepping, rather than defines the right-hand sides of the ODE system. Hence, the rhs format allows the model defining function to also directly modify the state vector, and correspondingly have the time step as one of the parameters. Currently, BeatBox has rhs definitions of the “conceptual” excitable models, such as FitzHugh-Nagumo [40–42], Barkley [43], and complex Ginzburg-Landau equation [44], and specifically cardiac models, such as Fenton-Karma [45], Luo-Rudy “LRd” [46], Courtemanche *et al.* 1998 [47], ten Tuschher *et al.* 2004 [48] and ten Tuschher-Panfilov 2006 [49].

The ionic format is suitable for solvers explicitly exploiting the specific structure of cardiac and neural excitation models, currently implemented in rushlarsen. This solver handles both the classical Rush-Larsen scheme, and its matrix modification described above. The difference from the rhs format is that the vector of dynamic variables is split into a part that corresponds to gating variables, Markov chain variables, and “other”, i.e. non-gating variables. Correspondingly, a module definining an ionic model is expected to export separate functions computing the transition rates for the gating and Markov variables, and ODE right-hand sides for non-gating variables. The current version of BeatBox has ionic definitions of the following models: Beeler-Reuter [50], Courtemanche *et al.* 1998 [47], ten Tuschher-Panfilov 2006 [49] and Hodgkin-Huxley [35].

Both rhs and ionic libraries of cell models can be extended by adding new models in the appropriate format. Instructions for that, with examples, are provided in the BeatBox documentation [30]. This includes an example of a semi-automatic conversion of a model from the CELLML format into a BeatBox rhs module. Making such conversion completely automatic is entirely feasible and is one of the planned directions of BeatBox development. Conversion to ionic format is more complicated as it requires syntactic distinction of gate and Markov chain variables and their transition rates from other variables, currently not available in the CELLML standard.

### Monodomain diffusion and boundary conditions

We focus here on the device diff which is the main device implementing the diffusion term in the monodomain diffusion, which mathematically can be described as:

$$Lu=\sum_{j,k=1}^{3}\frac{\partial }{\partial x_{j}}\left(D_{jk}\left(\vec{r}\right)\;\frac{\partial u}{\partial x_{j}}\right)$$ (13)

with the naturally associated non-flux boundary conditions,

$$\sum_{j,k=1}^{3}n_{k}D_{jk}\left(\vec{r}\right)\;\frac{\partial u}{\partial x_{j}}=0$$ (14)

where $\vec{n}=(n_{k})$ is the normal to the boundary Γ of the domain D, i.e. excitable tissue. Currently this is implemented for the transversely isotropic case, i.e. when the diffusion tensor $\hat{D}={\left(C_{m}\chi \right)}^{-1}{\hat{\sigma }}_{\text{eff}}=(D_{jk})$ has only two different eigenvalues: the bigger, simple eigenvalue *D*_∥_ corresponding to the direction along the tissue fibers, and the smaller, double eigenvalue *D*_⊥_, corresponding to the directions across the fibres, as this is the most popular case in modelling anisotropic cardiac tissue (the modification for the general orthotropic case is straightforward though). In this case,

$$D_{jk}=D_{⊥}\delta_{jk}+\left(D_{∥}-D_{⊥}\right)f_{j}f_{k},$$ (15)

where $\vec{f}=(f_{k})$ is the unit vector of the fiber direction. The simple finite-difference approximation of Eqs (13) and (14) in diff device is along the lines described, e.g. in [51]. In detail, we have

$${\left(Lu\right)}_{p}=\sum_{q\in {\left\{0,\pm 1\right\}}^{3}}W_{q}^{p}u_{p+q},$$ (16)

where $p\in Z^{3}$ is the 3D index of a grid node with position vector ${\vec{r}}_{p}$, $u_{p}=u\left({\vec{r}}_{p}\right)$ is the value of the field *u* at the grid node **p**, ${\left(Lu\right)}_{p}$ is the value of the diffusion operator approximation at that point, **q** ∈ {0, ±1}^3^ is the grid node index increment, and the weights $W_{q}^{p}$ are defined by the following expressions:

$$W_{q}^{p}={\bar{W}}_{q}^{p}+{\tilde{W}}_{q}^{p},$$ (17)

$${\bar{W}}_{q}^{p}=\frac{\psi_{p+q}}{h^{2}}\left\{\begin{array}{cc} D_{jj}^{p}, & q=\pm q_{j},\\ \frac{1}{2}D_{jk}^{p}, & q=\pm (q_{j}+q_{k}),\;j\neq k,\\ -\frac{1}{2}D_{jk}^{p}, & q=\pm (q_{j}-q_{k}),\;j\neq k,\\ 0, & q=\pm q_{1}\pm q_{2}\pm q_{3}, \end{array}\right.,$$ (18)

$${\tilde{W}}_{q}^{p}=\frac{\psi_{p+q}\psi_{p-q}}{4h^{2}}\left\{\begin{array}{cc} \pm \sum_{j=1}^{3}\left(D_{jk}^{p+q_{j}}-D_{jk}^{p-q_{j}}\right), & q=\pm q_{k},\\ 0, & \text{otherwise}, \end{array}\right.$$ (19)

$$W_{\left(0,0,0\right)}^{p}=-\sum_{q\neq (0,0,0)}W_{q}^{p},$$ (20)

where *j*, *k* ∈ (1, 2, 3), *ψ*_**p**_ is the grid indicator function of the domain D, that is, *ψ*_**p**_ = 1 if ${\vec{r}}_{p}\in D$ and *ψ*_**p**_ = 0 otherwise, $D_{jk}^{p}=D_{jk}({\vec{r}}_{p})$, **q**_1_ = (1, 0, 0), **q**_2_ = (0, 1, 0), **q**_3_ = (0, 0, 1), and *h* is the space discretization step.

The above discretization is probably the simplest possible approach; there are alternatives available, see for example [52], however these require extra information about D beyond the grid function *ψ*_**p**_. We assess the approximation properties of the simple scheme described above by solving the following initial-boundary value problem for the diffusion equation:

$$\frac{\partial u}{\partial t}=\frac{\partial^{2}u}{\partial x^{2}}+\frac{\partial^{2}u}{\partial y^{2}},\;\left(x,y\right)\in D,\;t\in \left[0,T\right];$$ (21)

$$u=J_{0}\left(\gamma \sqrt{{\left(x-x_{0}\right)}^{2}+{\left(y-y_{0}\right)}^{2}}\right),\;\left(x,y\right)\in D,\;t=0;$$ (22)

$$\frac{\partial u}{\partial \vec{n}}=0,\;\left(x,y\right)\in \Gamma ,\;t\in R_{+};$$ (23)

$$D=\left\{\left(x,y\right)|{\left(x-x_{0}\right)}^{2}+{\left(y-y_{0}\right)}^{2}\leq 1\right\},$$ (24)

$$\Gamma =\partial D=\left\{\left(x,y\right)|{\left(x-x_{0}\right)}^{2}+{\left(y-y_{0}\right)}^{2}=1\right\}.$$ (25)

Here J_0_(⋅) is Bessel function of the first order of index 0, and *γ* ≈ 3.8317… is the first positive root of its derivative, $J_{0}^{\prime }\left(\gamma \right)=0$. This problem has an exact solution:

$$u=J_{0}\left(\gamma \sqrt{{\left(x-x_{0}\right)}^{2}+{\left(y-y_{0}\right)}^{2}}\right)\;e^{-\gamma^{2}t}.$$ (26)

**Fig 3:**
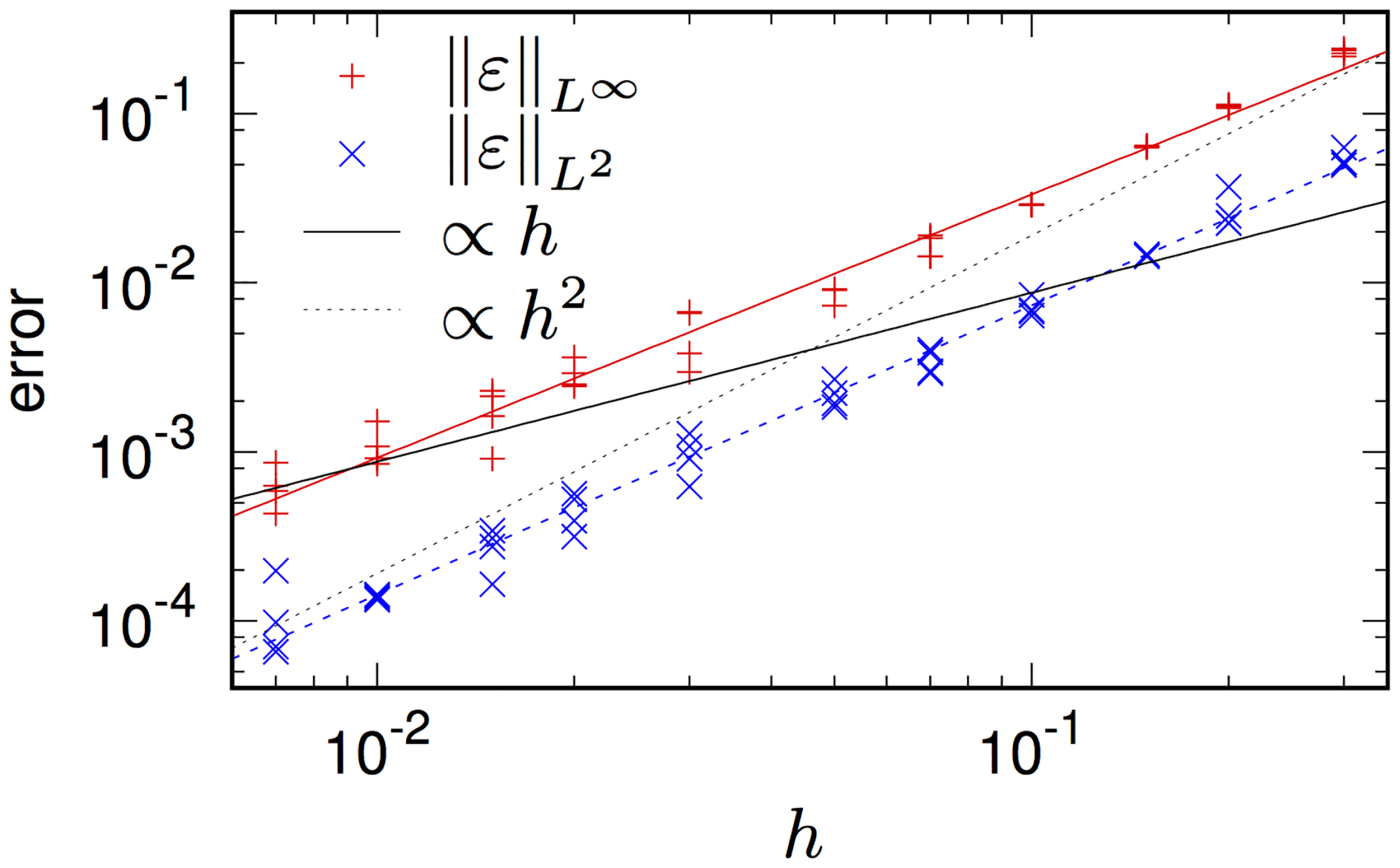
Numerical convergence of the solution of the problem Eqs (21)–(25). Slope lines are with slopes 1, 2 and best fits with slopes 1.564 for *L*^∞^, and 1.719 for *L*^2^.

Fig 3 illustrates the numerical convergence of the solution of the problems (21)–(25) by BeatBox using diff device to the exact solution provided by Eq (26), for *T* = 0.2. The timestepping is by using a forward Euler scheme with a time step of *k* = *h*^2^/80. The error, i.e. the difference $\epsilon \left(\vec{r},t\right)=u{}^{♯}\left(\vec{r},t\right)-u\left(\vec{r},t\right)$ between the exact solution $u(\vec{r},t)$ and its approximation obtained numerically, $u{}^{♯}\left(\vec{r},t\right)$, is characterized by two norms,

$${∥\epsilon ∥}_{L^{\infty }}=\max_{t\in [0,T]}\max_{\vec{r}\in D}\left|\epsilon (\vec{r},t)\right|,$$ (27)

$${∥\epsilon ∥}_{L^{2}}={\left(\frac{1}{T\mu \;\left(D\right)}\int_{0}^{T}\int_{D}{\left|\epsilon (\vec{r},t)\right|}^{2}\;d^{2}\vec{r}\;dt\right)}^{1/2}.$$ (28)

where μ(D) stands for the area of D, and all the integrals are calculated by the trapezoidal rule. Each *h* is represented by four points on each graph, corresponding to four simulations, with different position of the centre of the circle (*x*_0_, *y*_0_) with respect to the grid $\left({\vec{r}}_{p}\right)$, namely, $(\left(x_{0},y_{0}\right)-{\vec{r}}_{p})/h=\left(0,0\right)$, (0.2, 0.2), (0.2, 0.6) and (0.6, 0.6); this is to eliminate any possible effects related to special arrangement of the problem with respect to the grid. We can see that the convergence is worse than *h*^2^, but better than *h*^1^. The *L*^2^ norm of the error converges faster than *L*^∞^ norm, which is an indication that the main source of error is localized—this is, of course, to be expected, as the boundary conditions, in a sense, approximate the curvilinear boundary Γ with pieces of straight lines parallel to the *x* and *y* axes, thus typically making an error O(h). We stress that in cases where the realistic tissue geometry is available as a set of points with the same resolution as the computational grid, the knowledge of any curvilinear boundary is in any case unavailable, so any loss of accuracy associated with it, or, equivalently, any notional gain of accuracy that would be associated with using a curvilinear boundary instead, would be purely theoretical.

From the MPI viewpoint, the work of the diff device and other similar devices requires special care, since computation of the Laplacian of a dynamical field at a point requires knowledge of the field in neighbouring points, and some of the neighbouring points may be allocated to different threads. Hence, some message passing (exchange of interfacial buffers) is required for its work. This issue is discussed in detail in Section “Complex geometries and domain partitioning”.

### Bidomain diffusion

Computations using the bidomain tissue description Eq (1) differ primarily by the presence of the equation:

$$\nabla \cdot \left({\hat{\sigma }}_{i}+{\hat{\sigma }}_{e}\right)\nabla \Phi_{i}=\nabla \cdot {\hat{\sigma }}_{e}\nabla V-I_{\text{ext}},$$ (29)

which is elliptic with respect to both Φ_*i*_ and *V*. We have implemented a solver for elliptic equations in the elliptic device. This uses Full Multigrid iterations with vertex-centered restriction/prolongation operators with bi/tri-linear interpolation, and a (multicoloured) Gauss-Seidel or a Jacobi smoother.

The linear system to which the solver applies naturally occurs through discretization of the diffusion operator in the same way as described in the previous subsection. For solving the bidomain model (1) using operator splitting, the elliptic device can be used to solve the elliptic equation with respect to Φ_*i*_, leaving the parabolic diffusion equation for timestepping *V* using the diff device and timestepping *V* and **g** according to the reaction kinetics via an ODE solver, such as euler device.

It is straightforward to see that the solution to the problem Eq (29) with respect to Φ_*i*_ with non-flux boundary conditions is unique only up to an additive constant. One way to fix this constant is to use a nonlocal condition; e.g. [20] uses $\int \Phi_{e}\;d^{3}\vec{r}=0$. In BeatBox in this case condition Φ_*i*_(*x*_pin_, *y*_pin_, *z*_pin_) = *u*_pin_ is used instead, defined by parameters xpin, ypin, zpin and upin of the device elliptic. Since this condition is local, it does not create any issues with parallelization.

The MPI aspect of the elliptic device is similar to that of diff device, in that the Gauss-Seidel iterations involve neighbouring grid points which may be allocated to different threads, and this is considered in detail in Section “Complex geometries and domain partitioning”. The specifics of the elliptic device is that it implements an iterative algorithm, and buffer exchange is required at every iteration.

We illustrate this computation scheme on an example of a bidomain problem with an exact solution. We consider a bidomain system (1) with a one-component “cell model”, dim **g** = 0, corresponding to the Zeldovich-Frank-Kamenetsky [53] also known as Nagumo equation [54] and Schlögl model [55]:

$$\frac{\partial V}{\partial t}=V\left(V-\alpha \right)\left(1-V\right)+\left(D_{∥}^{i}\partial_{x}^{2}+D_{⊥}^{i}\partial_{y}^{2}\right)\Phi_{i},$$ (30)

$$\left[\left(D_{∥}^{e}+D_{∥}^{i}\right)\partial_{x}^{2}+\left(D_{⊥}^{e}+D_{⊥}^{i}\right)\partial_{y}^{2}\right]\Phi_{i}=\left(D_{∥}^{e}\partial_{x}^{2}+D_{⊥}^{e}\partial_{y}^{2}\right)V.$$ (31)

If posed on the whole plane, $\left(x,y\right)\in R^{2}$, this system has a family of exact solutions in the form of plane waves,

$$V^{*}={\left\{1+\exp\left[\left(x\cos\theta +y\sin\theta -s-ct\right)/\sqrt{2D_{*}}\right]\right\}}^{-1},$$ (32)

$$\Phi_{i}^{*}=KV^{*},$$ (33)

where the angle of the wave propagation, *θ*, and its initial phase, *s*, are arbitrary constants, and the other parameters of the solution are defined by $c=\sqrt{2D_{*}}(\frac{1}{2}-\alpha )$, $D_{*}=D_{*}^{i}D_{*}^{e}/\left(D_{*}^{i}+D_{*}^{e}\right)$, $K=D_{*}^{e}/\left(D_{*}^{i}+D_{*}^{e}\right)$, $D_{*}^{i}=D_{∥}^{i}\cos^{2}\theta +D_{⊥}^{i}\sin^{2}\theta$, $D_{*}^{e}=D_{∥}^{e}\cos^{2}\theta +D_{⊥}^{e}\sin^{2}\theta$.

For testing BeatBox as a bidomain solver, we consider the problem for the system Eqs (30) and (31) in a square domain of size *L*, $D={\left[0,L\right]}^{2}$, for a time interval *t* ∈ [0, *T*], with the initial and (non-homogeneous Dirichlet) boundary conditions set in terms of Eqs (32) and (33) as

$$V\left(x,y,0\right)=V^{*},\;\Phi_{i}\left(x,y,0\right)=\Phi_{i}^{*},\;\left(x,y\right)\in D;$$ (34)

$$V\left(x,y,t\right)=V^{*},\;\Phi_{i}\left(x,y,t\right)=\Phi_{i}^{*},\;\left(x,y\right)\in \Gamma ,t\in \left(0,T\right),$$ (35)

Γ=∂D={0,L}×[0,L]∪[0,L]×{0,L}. (36)

To implement solution of the problem Eqs (30)–(36) in a BeatBox script, we split it into substeps:

$$\begin{array}{ll} \text{dif}f(1) & :\;S_{1}=\nabla \cdot {\hat{D}}^{e}\nabla V,\\ \text{elliptic} & :\;\nabla \cdot \left({\hat{D}}^{e}+{\hat{D}}^{i}\right)\nabla \Phi_{i}=S_{1},\\ \text{diff}(2) & :\;S_{2}=\nabla \cdot {\hat{D}}^{i}\nabla \Phi_{i},\\ \text{euler} & :\;V_{t}=V\left(V-\alpha \right)\left(1-V\right)+S_{2} \end{array}$$

The resulting fragment of BeatBox script looks like this:

**Table:** 

def str domain x0 = xil x1 = xir y0 = yil y1 = yir;
// source term in the elliptic equation
diff [domain] v0 = [u] v1 = [s]
Dpar = Dex Dtrans = Dey hx = hx;
// solving the elliptic equation
elliptic [domain] v0 = [s] v1 = [p]
Dpar = Dex+Dix Dtrans = Dey+Diy hx = hx
tolerance = tol delta = 0.5 upper level = 3
vcycles = 20 preiter = 1 postiter = 2 maxiter = 1e6;
// source term in the parabolic equation
diff [domain] v0 = [p] v1 = [s]
Dpar = Dix Dtrans = Diy hx = hx;
// timestep of the parabolic equation
euler [domain] v0 = [u] v1 = [u]
ode = zfk ht = ht par = {alpha = alpha Iu = @[s]};

In this fragment, the first diff device computes the right-hand side of the elliptic Eq (31), $S_{1}=\nabla \cdot {\hat{D}}^{e}\nabla V$, and deposits the auxiliary variable *S*_1_ into the layer [s]; then elliptic device solves the elliptic eq (31) for Φ_*i*_, using the provided fine-tuning algorithm parameters, and puts the result into the layer [p]. The second diff device computes $S_{2}=\nabla \cdot {\hat{D}}^{i}\nabla \Phi_{i}$ and puts the result *S*_2_ into the layer [s] (which is therefore “recycled”), and the euler device does the time step of the cell model. The interior of the domain D is mapped to the subgrid [domain] with the grid *x*-coordinate from xil to xir and *y*-coordinate from yil to yir. The non-homogeneous, non-stationary Dirichlet boundary condition (35) were implemented by a k_func device, computing the boundary values for *V* and Φ_*i*_ for the grid nodes surrounding this subgrid [domain], i.e. those with grid coordinates xil-1,xir+1,yil-1,yir+1 (this part of the script is not shown).

**Fig 4:**
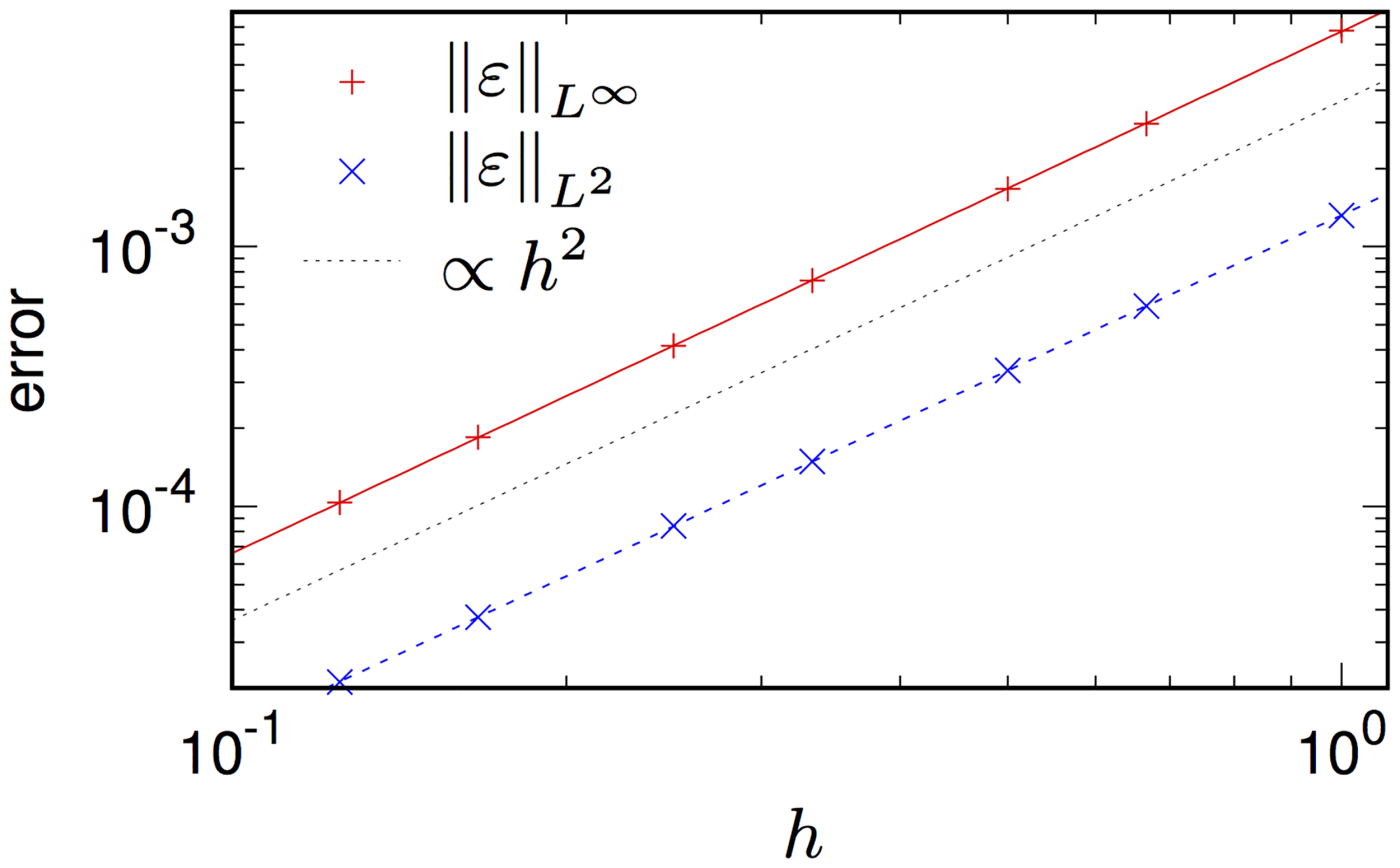
Numerical convergence of the solution of the problem Eqs (30)–(36). Slope lines: slope 2 (black) and best fits with slopes 2.009 for *L*^∞^, and 1.9889 for *L*^2^.

The accuracy of the computational scheme is illustrated in Fig 4. We take *L* = 10, *T* = 40, *α* = 0.13, $D_{∥}^{i}=2$, $D_{⊥}^{i}=0.2$, $D_{∥}^{e}=8$, $D_{⊥}^{e}=2$ and *s* = −5. The time step $k=3h^{2}/(16D_{∥}^{e})$ is varied together with the space step *h* with the coefficient $3/(16D_{∥}^{e})$ chosen from the considerations of numerical stability [51], resulting in quadratic convergence of the algorithm, as should be expected.

### Complex geometries and domain partitioning

#### Format of BeatBox geometry files

Complex geometries for BeatBox simulations are defined by files in BeatBox’s “.bbg” format. A .bbg file is an ASCII text file, each line in which describes a point in a regular mesh. Each line contains comma-separated values, in the following format:

**Table:** 

x, y, z, status, fibre_x, fibre_y, fibre_z

Here x, y, z are integer Cartesian coordinates of the point, status is a flag, a nonzero-value of which shows that this point is in the tissue, whereas a zero status designates a point in the void, and fibre_x, fibre_y, fibre_z are *x*-, *y*- and *z*-components of the fibre orientation vector at that point. To reduce the size of .bbg files, only tissue points, i.e. points with nonzero status need to be specified. BeatBox will ignore the fibre orientation vectors of void points in any case.

#### Anatomically realistic geometries

To use DT-MRI anatomical data in BeatBox simulations, such data should be converted into the .bbg format. The current version of BeatBox makes use only of the primary eigenvector of the diffusion tensor, which is why only one direction vector is used in the format. Once DT-MRI data on tissue points locations together with the corresponding fiber orientations are compiled into a .bbg anatomy file, it can be called from a BeatBox simulation script, see *e.g.* the statement

**Table:** 

state geometry = ffr.bbg anisotropy = 1 vmax = 4;

in sample.bbs script in Listing 2 in the Appendix.

#### Domain partitioning

**Fig 5:**
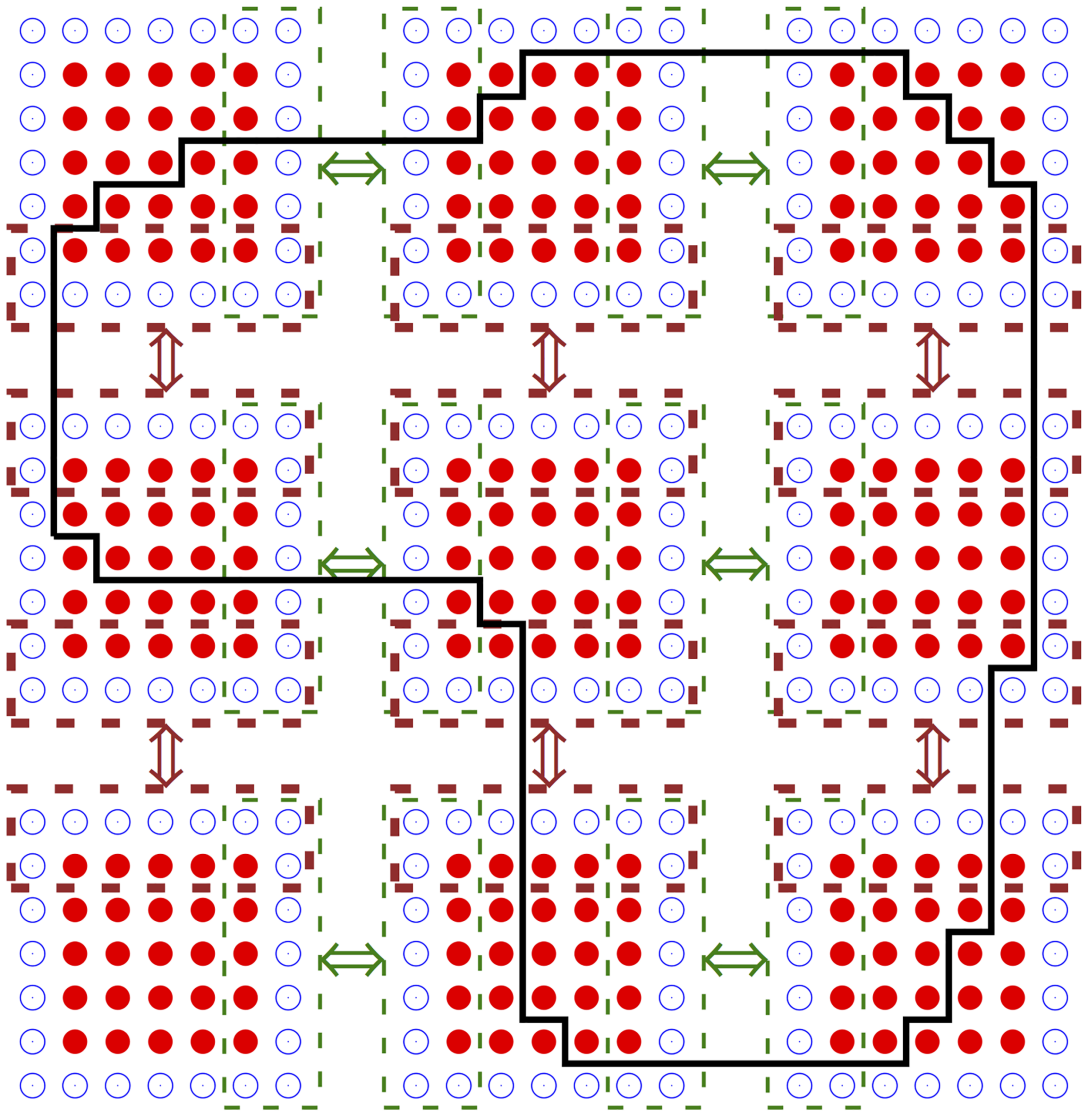
Schematic of the domain partitioning in MPI implementation of BeatBox. Solid circles represent nodes on which actual computations are done, empty circles are the “halo” points, the rectangles denote the exchange buffers and the solid black line represents the boundary of an irregular computational domain (excitable tissue).

Sharing work between processes in the MPI version of BeatBox is done by splitting the computational grid into **subdomains**, such that computation in each subdomain is done by a process. The method of domain decomposition is illustrated in Fig 5, for a 2D domain. Each of the *x*, *y* and *z* dimensions of the grid is separated by a certain number of equal subintervals (approximately equal, when the grid size is not divisible by the number of subintervals). The number of subintervals in different dimensions do not have to be the same. In the example shown in Fig 5, the *x* and *y* dimensions are split to have 3 subintervals each; the *z* dimension is not split. The grid nodes, in which computations are done, are represented in Fig 5 by solid circles (“bullets”).

The continuity of computations across subdomains necessary for devices involving the diffusion operator is achieved by using message passing with **exchange buffers**. The depth of the exchange buffer in each direction is one grid point. This imposes a limitation on the stencils that can be used by diffusion-like devices, such as a 9-point stencil for 2D and up to a 27-point stencil for 3D. In Fig 5, the hollow circles represent the fictitious grid nodes which are images of corresponding nodes from neighbouring subdomains, and the dashed lines designate the whole buffers, including the nodes to be sent and nodes to be received. The buffer exchange should be effected twice (forwards and backwards) for each dimension, i.e. four exchanges in 2D simulations and six exchanges in 3D simulations. If the buffer exchanges are done in the correct order, then this will ensure correct exchange of node values in the diagonal directions as well (**magic corners**, see [8] for details).

In bidomain computations, the buffer exchanges can be done either before each iteration or more seldom; in the latter case the result is different from the sequential run, but inasmuch as both MPI and sequential results are close to the actual solution, the difference between the two should be negligible. Similar consideration applies to the use of the Gauss-Seidel smoother, which of course will also give different results if applied in each subdomain separately.

When working with complex (non-cuboidal) domains, the BeatBox approach is to inscribe the domain into the smallest cuboid, and then proceed as before, with the difference that computations are only done at those grid nodes that belong to that domain, and the one outside domain remain idle. This is also illustrated schematically in Fig 5, where the boundary of the irregular domain is drawn by a closed bold solid black line. This creates a challenge to the performance: with high-degree parallelization and complicated geometry of the irregular domain, the load imbalance between processes can become significant; in particular, a large number of partitions will contain no points of the domain (in Fig 5, there is one such partition, in the bottom left corner). This problem is well known and there are efficient tools for solving them for structured as well as unstructured grids, see e.g. [56, 57]. In the current version of BeatBox, however, only the crudest optimization method is used: the partitions that are completely idle are not allocated to processes, which considerably limits the expected slow-down because of the uneven load (roughly speaking, at worst twice on average).

### Input/Output and visualization

Finally, we briefly mention some input/output options currently available in BeatBox. Each of these options is implemented in the corresponding device. This includes:

- Full precision binary input and output of a specified subset grid, by the devices load and dump respectively. In the MPI mode, these devices operate in parallel, using MPI_File_read_all and MPI_File_write_all calls respectively. All threads get collective access to the file using MPI_File_set_view calls at the start-up time, taking into account (computing the intersection of) both the 4D subset of the grid data allocated to the device, and the subdomain allocated for the particular thread.
- Discretized “fixed-point” (1 byte per value) output of three selected layers of a subset of the grid, by the device ppmout. MPI implementation of ppmout device is very similar to that of the dump device: use of MPI_File_write_all calls for writing after arranging collective access via MPI_File_set_view calls at the start-up time. The main difference is that ppmout device outputs only one byte per value instead of eight for dump. Therefore ppmout precision is typically not sufficient for the ppmout output data to be used as control points or initial conditions for subsequent BeatBox runs, though sufficient enough for most visualization purposes.
- Plain text outputs of a defined subset of the grid by the device record, and a list of expressions involving global variables, by the device k_print. The MPI aspects of the record and k_print devices are very different. The record device is similar to the dump and ppmout devices: it uses MPI_File_set_view and MPI_File_write_all for parallel output of a certain 4D subset of the grid in a fixed-lengh ASCII format, so the position of each text record in the output file can be precisely calculated. The k_print device outputs a specified list of values which may be expressions depending on global variables, which by definition are equally known to all threads. Hence the output is done only by one dedicated thread. All the MPI work needed here is selection of the communicating thread.

Some computational devices also have i/o options. For instance, device k_func, which performs computations by formulas specified in the BeatBox script, can also read data from of a plain text file; such data are interpreted as a tabulated univariate vector-function and is often used to create initial conditions by the phase-distribution method [58]. This read in from a file is done via sequential calls, which in the MPI mode may create some delay, but since this is done only once at the start-up time, we do not consider this a significant issue.

Another example is device singz, which finds phase singularities in *z*-cross-sections of the grid. These are defined as intersections of isolines of two fields allocated to selected layers of the grid. Coordinates of the intersection points within grid cells are defined using linear interpolation of the pieces of the isolines. In addition to assigning the coordinates of the singularity points to global variables, it can also output those to a plain text file and/or visualize. The MPI implementation of the singz device is slightly more complicated than in other devices. The singularity points can be found in any threads, and their number is not known *a priori*. Hence the coordinates of these points are passed, by MPI_Send to one dedicated “coordinating” thread, which collates reports from all participating threads obtained via MPI_Recv, and then assigns statistics of the found singularity points (their number, and means and standard deviations of their coordinates) and outputs, if requested, their coordinates to a file using sequential access. Naturally, the participating threads have to submit an empty report even if no singular points are found, as otherwise the coordinating thread has no means of knowing what messages to expect. This potentially creates an unnecessary delay compared to the sequential version; however in practice this is not noticeable since this device is usually called only during a small fraction of computation steps. Many devices have an optional debug parameter for printing plain text messages about details of their work. The MPI versions of these options depend on whether the device operates with grid data or global variables, and is based on the same principles as described above.

Regarding run-time visualization, the sequential version of BeatBox has a number of devices for 0D, 1D, and 2D visualization via X11 protocol if available (devices k_draw, k_plot, k_paint). 3D output typically requires much more tuning in order to be effective. Theoretically, one possibility is to do the tuning in the interactive mode while the computations are stopped, as it is done e.g. in EZSCROLL, see [59]; this, however, would go against one of the principles of BeatBox, that all details of the run are specified in the input script, so that any simulation is reproducible. Instead, currently the 3D visualization is done by post-processing of the output data, most often for the data produced by ppmout.

## Results

### Parallel scaling performance

**Fig 6:**
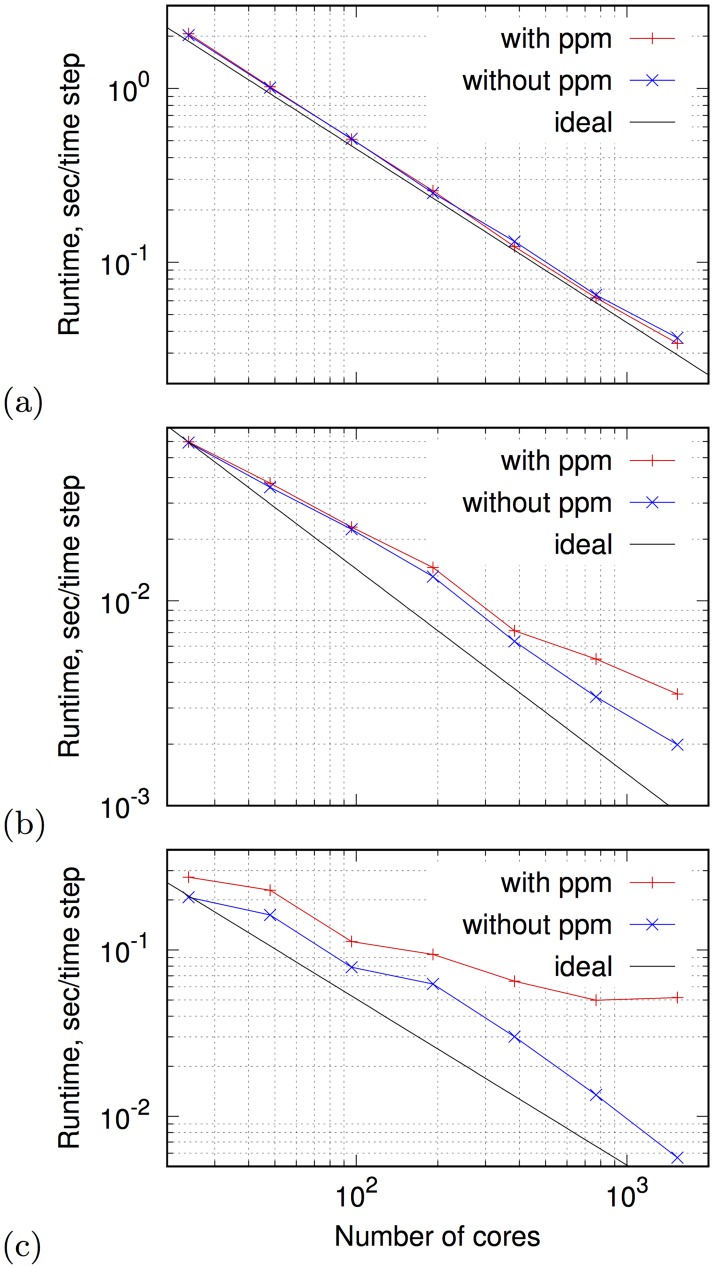
Log-log plots: The wall clock time per one time step in the simulation job, vs the number of cores. (a) Full box; (b) Rabbit ventricle geometry, (c) Human atrium geometry. In all plots, “with ppm” stands for performance including file output via ppmout device, “without ppm” stands for pure computations, and “ideal” is the perfect-scaling extrapolation of the performance achieved on the smallest number of cores.

Fig 6 illustrates the computation time taken by the MPI version of BeatBox on ARCHER (UK national supercomputing facility, http://www.archer.ac.uk/) as a function of the number of processes, for three series of test jobs, presenting different challenges from the parallelization viewpoint. In all series, the jobs simulated the monodomain model (2) with Courtemanche et al. 1998 model of human atrial cells [47], with dim **g** = 23, and anisotropic diffusion (19-point stencil), but with different geometries. Fig 6(a) is for a cubic grid of 300 × 300 × 300 points. Fig 6(b) is for the rabbit ventricles geometry, described in [60], which is inscribed in a cuboid grid 119 × 109 × 131, containing 470324 tissue points, which is about 27.7% of the total number of points in the grid. Fig 6(c) is for the human atrial geometry [61], inscribed into a cuboid grid 237 × 271 × 300, containing 1602787 tissue points, about 8.3% of the total. The human atrium geometry tests were using the crude optimization of partitioning, i.e. the subdomains that do not contain tissue points are not allocated to processes; the rabbit ventricle jobs are done without such optimization. In all job series, the simulation was with an initial condition of a scroll wave with a filament along the *y*-axis, using the procedure described e.g. in [62]. Simulation series for all three geometries were run with and without output via ppmout device, which outputs up to three selected layers of the grid, discretized to the 0…255 scale (3D extension of the ppm format of Netpbm, [63]), using parallel output (see Section “Input/output and visualization” for detail). The corresponding curves in the graphs are marked as “without ppm” and “with ppm” respectively. Such outputs were done once in every 200 timesteps for the full box and human atrium geometries, and once in every 1000 timesteps for the rabbit atrium geometry. The full box and human atrium jobs also did plain text, sequential output via k_print device (see Section “Input/output and visualization” for detail) of the activity at a single point once in every 20 time steps. To test the expenses associated with sophisticated control, the full box and human atrium jobs implemented feedback-controlled stimulation, similar to that implemented in the sample.bbs script described in the Appendix. To exclude the effects of the time taken by the start-up operations, we computed the time per step by running jobs identical in all respect except the number of time steps, and then considering the difference. ARCHER has 24 cores per node, and the numbers of processes in the test jobs are power-of-two multiples of 24. The “ideal” lines are drawn based on the result of the “without-ppm” job on 24 processors.

We observe that the effect of feedback control and plain text outputs on the parallel performance is relatively small, and the main slow-down at high parallelization happens due to uneven load of the processes. The parallel scaling is, as expected, best for the full box: without ppmout it is close to ideal for up to 1536 processes, while the curves for the complicated geometries deviate from ideal noticeably. Human atrium geometry is proportionally “much thinner” than the rabbit ventricle geometry, and the deviation from ideal is more pronounced in Fig 6(c) than in Fig 6(b). However, the detrimental effect of the uneven load is limited: notice that the “without ppm” curve in Fig 6(c) is almost parallel to the ideal in the interval of 192–1536 cores, and the slow down is only slightly more than by a factor of two. Another significant factor is the bulk output. In the human atrium geometry, such outputs were 10 times more frequent, and their effects is more pronounced overall and starts increasing at smaller parallelizations. Notice that the relative effect of the bulk output is much less in the full box: an obvious explanation is that the ppmout format always outputs the full enclosing grid while computations are only done in the tissue nodes, hence the output/computation ratio is about 12 times bigger for human atrium than in the full box.

Fig 6(a) shows that on a standard test of full 3D box computation BeatBox parallel performance is in accordance with expectations and satisfactory. The maximal efficient parallelization is of course problem-dependent, as illustrated by Fig 6(b) and 6(c). Fig 6(b) presents results of simulations of a small rabbit heart with less then 10^6^ grid tissue points, so at a larger number of cores, the inter-process communication expences take over the computation scaling. Fig 6(c) represents results of realistic simulations of a complex and “thin” human atrium, with less than 2 × 10^6^ grid tissue points. This illustrates the fact that parallel performance scaling depends on the ratio of inter-process communication costs to computations costs within one process, and for the cardiac modelling applications, this depends on the tissue geometry. We have verified that this ratio, and resulting perfromance, also depend on the excitation kinetic model: scaling is better if the kinetic model is computationally more complicated (these results are not shown). Also, for the simulations with complex and “thin” geometries, a significant improvement may be achieved by optimizing bulk outputs.

For the avoidance of doubt, we stress again that the performance results presented in Fig 6 are per time step of simulation, and exclude the time spent on any start-up operations, such as parsing the BeatBox script, reading the geometry file if used, domain partitioning if in MPI mode, tabulating the complicated functions if using ionic models, etc. This is done deliberately as these one-off operations typically take only few seconds at most altogether, and for realistic simulations are negligible.

### Examples of use in recent and current research

BeatBox or its predecessors has been used to produce simulation results presented in dozens of publications, e.g. [58, 62, 64–70]. In this section, we mention a handful of recent and representative studies, illustrating the key features of this software.

**Fig 7:**
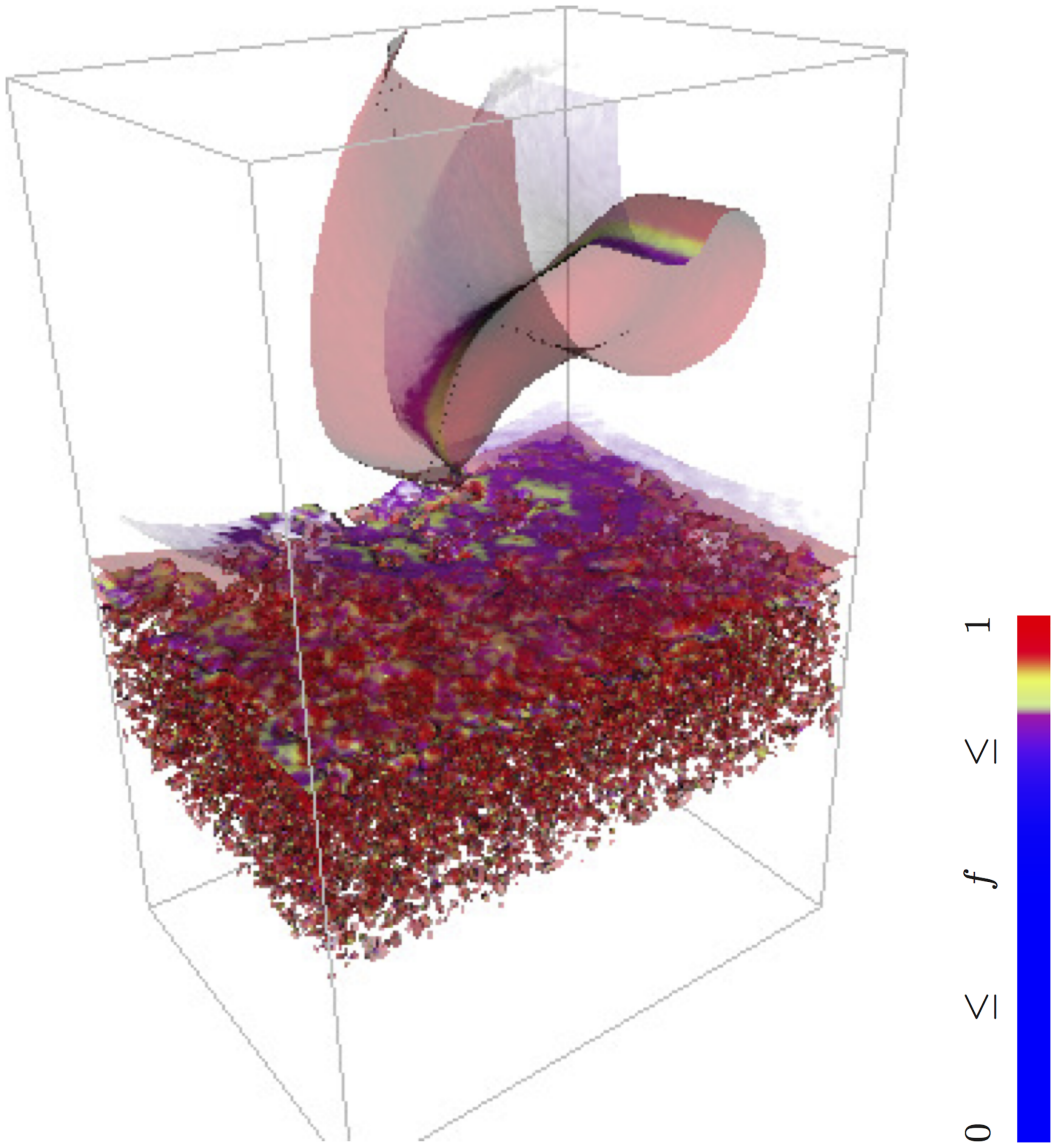
Scroll wave generation from ischaemic border zone. Generation of a scroll wave out of microscopic re-entries in excitable medium with random, space- and time-dependent distribution of parameters, modelling movement of ischaemic border zone during reperfusion [69]; Beeler-Reuter [50] kinetics.

Fig 7 illustrates a complicated simulation set-up, which we believe would not be possible in other software currently available without re-coding at low-level requiring assistance from the developers. This figure is an example from [69] which modelled arrhythmogenic mechanisms of the boundary layer between ischaemic and recovered cardiac tissue, moving due to reperfusion. The model assumed that a certain “excitability” parameter (decrease in permeability of the inward potassium rectifier current $i_{K_{1}}$) varied in space and time due to two factors: firstly, space-only random distribution due to properties of individual cells; secondly, deterministic smooth transition between low excitability of the ischaemic tissue and high excitability of the recovered tissue, changing in time due to reperfusion. On top of that, the isotropic diffusion coefficient also varied along a similar transition between low diffusivity of the ischaemic tissue and high diffusivity of the recovered tissue, of a profile different from, but moving synchronously with, the profile of the excitability parameter. Fig 7 shows isosurfaces of the transmembrane voltage field, *V* = −35mV, painted according to the value of calcium current gating variable *f*, as shown by the colourbar. In the snapshot shown, the upper half of the box is a recovered, well connected, well excitable medium, which supports a macroscopic scroll wave. The layer below it consists of ischaemic boundary cells that are less connected, so some of the cells (those where $i_{K_{1}}$ suppressed to a level below a certain threshold) are in the self-oscillatory regime leading to a micro-scale turbulent excitation pattern. The lowest layer consists of ischaemic cells with suppressed excitability so they are not electrically active. As the parametric profiles slowly move downwards, the solution represents the process of recovery from ischaemia, which produces a reperfusion arrhythmia as a result. All these spatio-temporal variations in parameters have been set up not by writing special C code for it, but at the BeatBox script level using k_func device. Further detail can be found in [69].

**Fig 8:**
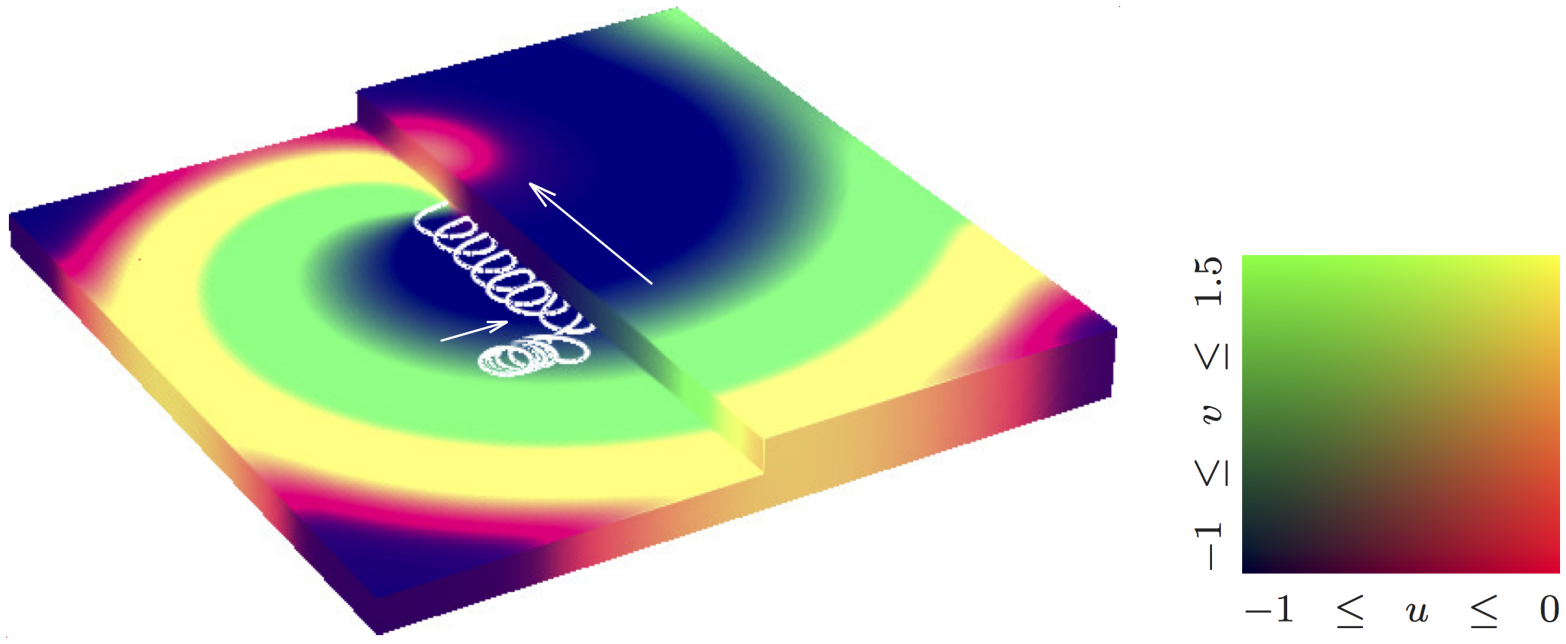
Drift along a thickness step. Drift of scroll wave along a thickness step [67], FitzHugh-Nagumo kinetics.

**Fig 9:**
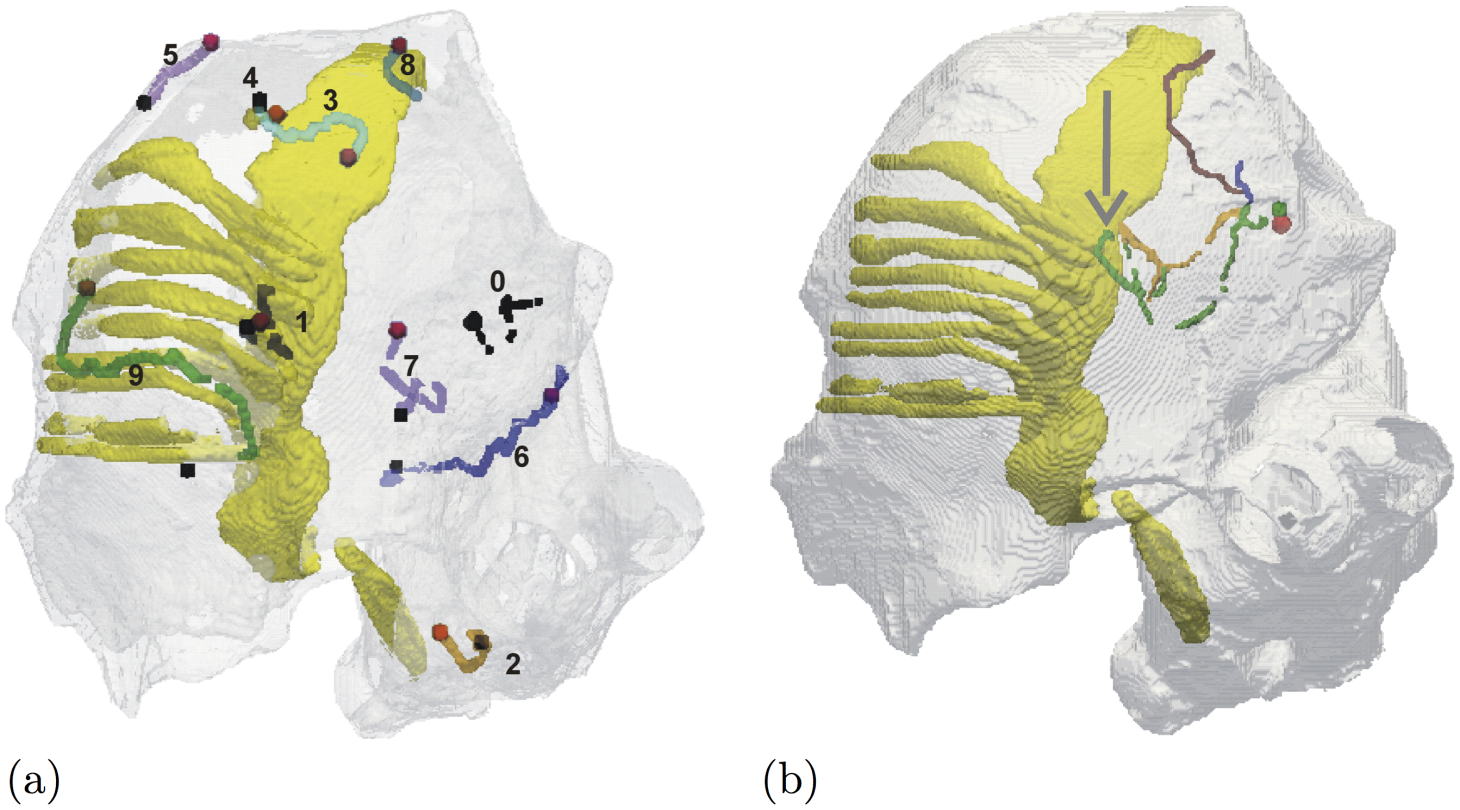
Drift in a realistic human atrium geometry. Drift of scroll wave in a realistic human atrium geometry [61], Courtemanche et al. [47] kinetics. (a) Trajectories of spontaneous drift, caused purely by the anatomy features [62]; (b) Trajectories of resonant drift, caused by feedback-controlled electrical stimulation [70].

**Fig 10:**
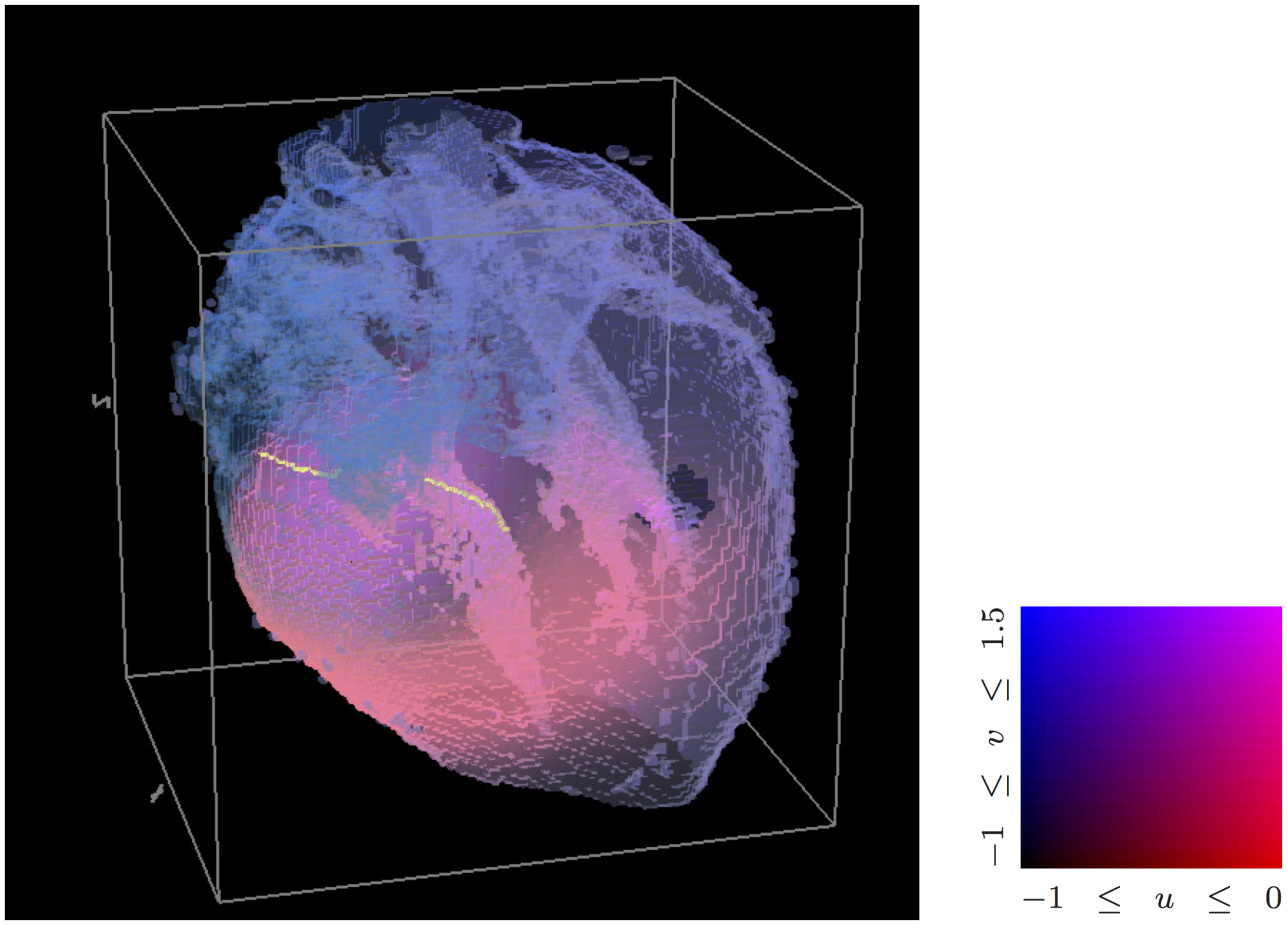
Scroll waves of excitation in a DT-MRI based model of human foetal heart. A snapshot of excitation pattern with scroll wave filaments in human foetal heart anatomy [71], FitzHugh-Nagumo kinetics. The surface of the heart is shown semitransparent, colour-coded depending on the values of the *u* and *v* variable as shown in the colourbox on the right. The yellow lines are the scroll filaments inside the heart. The human foetal heart DT-MRI data sets used in the BeatBox simulation presented here were provided by E. Pervolaraki et al. [71]. The simulation shown is part of the ongoing project on cardiac re-entry dynamics in DT-MRI based model of human foetal heart. The full paper by R.A. Anderson, F.C. Wen, A.V. Holden, E. Pervolaraki, and I.V. Biktasheva is in preparation.

Figs 8, 9 and 10 illustrate simulations in non-cuboid domains. Fig 8 shows a surface view of a simulation in an artificially defined domain, used to quantitatively test predictions of an asymptotic theory about the drift of a scroll wave in a thin layer due to sharp variations of thickness. This simulation uses two-component FitzHugh-Nagumo kinetics. Shown is the surface view at a selected moment of time, colour coding represents states of the activator variable (red colour component) and inhibitor variable (green colour component) on dark-blue background, as shown by the “colourbox” to the right of the main picture; the white line shows the trajectory of the tip of the spiral wave seen at the upper surface of the domain for a period of time preceding the presented view. In this series of simulations, precise initial positioning of the scroll wave was required, which was achieved with the help of the “phase distribution” method implemented in BeatBox’s k_func device.

Fig 9 illustrates simulations in an anatomically realistic model of human atrium, on a regular cuboidal MRI-type grid (although the actual grid origin was different, see [61]). Fig 9(a) illustrates the anatomy-induced drift [62]. Shown are a number of trajectories of tips of spiral waves appearing on the surface of the atrium nearest to the viewer; the yellow background indicates prominent anatomical features (the pectinate muscles and the terminal crest). To make the visualization clearer, the trajectories are represented by lines connecting tip positions separated by exactly one period of rotation (“stroboscopic view”); shown are several trajectories starting at different initial positions and made within equal time intervals. Different trajectories are shown by different colours and enumerated. The beginning of each trajectory is shown by a red point, and the end of the trajectory is shown by a black point.

Fig 9(b) illustrates further the BeatBox’s capability for complicated simulation protocols. This panel displays results of simulations in the same model as those shown in Fig 9(a), but now the initial position for the scroll wave is chosen far from any sharp features so that the anatomy-induced drift is not pronounced, and instead, the scroll wave is subject to low-voltage pulses of external electric field, *I*_eff_ = *I*_eff_(*t*). The delivery of the pulses is controlled by a feedback protocol similar to that illustrated by the sample script Listing 2, namely:

- There is a “registration electrode”, at a point on the surface of the atrium that is the most distant from the viewer, position of which is indicated by the grey arrow.
- The signal from the registration electrode is monitored for the moment of arrival of an excitation wave, defined as the moment when the transmembrane voltage crosses a certain threshold value upwards.
- From the moment of the front arrival to the registration electrode, a certain waiting interval (delay) is observed.
- On expiration of the delay interval, a pulse of *I*_eff_(*t*) of a certain duration and certain amplitude is applied.

In Fig 9(b) we see three different trajectories starting at the same point: they differ in the value of the delay interval between registration of the front arrival and delivery of the electric pulse. This stimulation protocol is aimed at achieving low-voltage defibrillation; the presented simulation illustrates the possibility of moving the location of a re-entrant arrhythmia by electrical stimuli of a magnitude much smaller than required for the classical single-shock defibrillation, in anatomically realistic setting, in different directions by adjusting the details of the feedback loop. This protocol is implemented fully at the BeatBox script level, and we believe this would not be possible in any other software without re-coding at low level.

Both panels of Fig 9 show tip trajectories starting at purposefully selected points; a specific challenge in this case was that for this particular study, it was important to have initial conditions that have only one phase singularity within the tissue, while the opposite one (necessary for topological reason) was about the big opening corresponding to the atrio-ventricular border. Again, the initial positioning of the scroll waves was done using the “phase-distribution” method, implemented with the help of the k_func device at the BeatBox script level. Also, both series of simulations used “stroboscopic” output, when the output data files were created in synchrony with a front of simulated excitation wave registered at a certain point of the medium; this also was implemented entirely at the BeatBox script level.

Fig 10 shows a volume view of a scroll wave in a human foetal heart geometry, obtained by DT-MRI [71]. Shown is the surface of the heart, semi-transparent and colour-coded depending on the values of the dynamic variables *u* and *v* of the FitzHugh-Nagumo kinetics Eq (3) chosen here for illustrative purposes. The red component represents the *u* value, the blue component represents the *v* value, and the resulted colour-coding is summarised in the “colourbox” to the right of the main picture. The yellow lines traversing the ventricular wall are the scroll filaments, defined as intersection of a *u* = const surface with a *v* = const surface.

The visualization in all cases was done by post-processing of the simulation data. For Fig 7, we used Iris Explorer [72]. Both panels of Fig 9 were generated with ParaView [73]. Figs 8 and 10 were produced by an in-house visualizer, based on the graphical part of Barkley and Dowle’s EZSCROLL [59, 74], which is in turn based on the Marching Cubes algorithm [75, 76].

## Conclusion

The leading idea underlying BeatBox development is robustness, portability, flexibility and user-friendliness in the first place, connected with efficiency as an important but secondary consideration. In the present form, BeatBox can be exploited in sequential and parallel (MPI) modes, with run-time and/or post-processing visualization, on any unix-like platform from laptops to supercomputing facilities. The modular structure of BeatBox effectively decouples the user interface, which at present is a scripting language used to construct the ring of devices, from the implementation of the computationally intensive stages in individual devices. The current computational capabilities can be and will be further expanded in accordance with the needs of wider usership without changing the backbone ideology.

As far as MPI features are concerned, the straightforward approach to parallelization via domain decomposition, yields acceptable results. As the maximal efficient parallelization is problem-dependent, small to medium scale anatomically realistic simulations become inefficient for number of threads beyond about 1000. As higher-resolution DT-MRI anatomical data become available and/or more detailed kinetic models are used, the adequate parallelization should be expected to increase. However, BeatBox already offers an important possibility of MPI utilisation of in-vivo MRI human heart anatomical data for real time simulations on multi-core desktop workstations for e.g. individualised ablation strategies, thus further broadening the MPI end users community.

Apart from the size of the problem, another important limiting factor is the uneven load of the parallel threads for “thin” complex geometries of the computational domains, and output, which determines possible direction of further development. The uneven load can be addressed by a more careful fine-tuning of domain decomposition to specifics of particular domain geometry, which to some extent may be achieved without violating the main principles of the domain decomposition, by allowing uneven partitioning along the coordinate axes.

The slow down in cases of extensive output is a problem which is not specific for BeatBox; however, some improvement in some cases may be achieved by making any input-output operations exclusive to one or more designated threads specializing on this and relieved from computational load as such.

As the current BeatBox solvers use finite difference, regular grid ideology, incorporation of DT-MRI regular cartesian grid anatomy models into BeatBox simulations is straightforward, as illustrated by Figs 9 and 10, without a meshalizer step required for finite element/finite volume solvers. However, architecturally there is no fundamental problems in extending BeatBox functionality to the finite element approximation as long as regular mesh of finite elements is used that can be mapped to a rectangular array. Extension to irregular meshes would require more substantive changes, however the main idea of the ring of devices may be useful there as well. The same concerns the “discrete multidomain” model of in [77, 78], which describes cardiac tissue on the microscopic level. Although one could attempt to embed this description into a regular grid, the most efficient implementation would require very different data structures.

Other relatively straightforward developments consistent with BeatBox paradigm to be implemented in the foreseeable future, include:

- Generalization of diff and elliptic devices for the orthotropic case.
- Partitioning of the grid to domains described by different models. This can be used e.g. to model whole heart or its parts consisting of different tissues, surrounding bath or torso etc.
- Fully automatic conversion of CELLML cellular models into the rhs format.
- If and when the syntax of CELLML is enriched so as to explicitly identify gating and Markov-chain variables and their dynamics, fully automatic conversion of those into the ionic format.
- Run-time 3D graphics (currently there is only 2D run-time graphics, and 3D visualization is done by post-processing).

## Availability

BeatBox is free software available to download from the BeatBox home page [79]. The source code is distributed under version 3 (or later) of the GNU general public licence. The BeatBox software is also available in the Supporting Information file S1_code.zip. BeatBox is designed to be used in Unix-like operating systems, in non-interactive mode (started directly from the command line or by a shell script), with or without run-time graphics. The parallel version requires MPICH or OpenMPI, but the sequential version can be compiled and run without those. For the run-time graphics, X11 is required, including its GL extension for some devices, but the computational part can be compiled and run without those. Installation is done through the standard configure—make—make install command sequence, assuming that the environment includes bash, make and a C compiler. Modifications to BeatBox, such as adding new modules, would require the autoconf toolset. There are no other dependencies. Detailed installation instructions in HTML format are provided in the documentation supplied with the distribution [30, 79], also in S1 Code. The Matrix Rush-Larsen part of the rushlarsen device uses an eigenvalue solver from GSL library, but all relevant bits from GSL and its dependency CBLAS are included within the BeatBox distribution, so the user need not worry about installing those separately, nor about the version compatibility.

### Appendix: Examples of BeatBox scripts

#### Script 1: ez.bbs

Listing 1 provides a “minimalist” example of a BeatBox script. It approximately emulates the functionality of Barkley’s EZSPIRAL [80] (except tip finding and recording, saving the final state, and starting from a previously saved state). Namely, it performs a simulation of the Barkley model [43] on a 2D grid consisting of 100 × 100 internal points; one extra row of points in each direction is added to implement the boundary conditions. The initial conditions are specified using “instant cross-field” protocol:

$$u=\{\begin{array}{ll} 1, & y>y_{*},\\ 0, & \text{otherwise}, \end{array}\;v=\{\begin{array}{ll} 0.4, & x>x_{*},\\ 0, & \text{otherwise}, \end{array}$$

where (x_*_, y_*_) is the centre of the box. Every 10 time steps, it plots the solution in an OpenGL window (using the colour-coding similar to that of Fig 8), and outputs the dynamic variables into a text file.

**Listing 1:** BeatBox script ez.bbs. A simple BeatBox script.

/* Box of 100x100 internal points, 3 layers */
state xmax = 102 ymax = 102 vmax = 3;
/* Schedule control flags */
def real begin; // true only at the beginning
def real out; // true when graphic and text outputs are due
def real end; // true when all done
/* The schedule: this k_func computes only global variables, at each t */
k_func nowhere = 1 pgm = {begin = eq(t,0);out = eq(mod(t,10),0);end = ge(t,1000)};
/* Init. cond.: this k_func computes only local field values, at t = 0 only */
k_func when = begin pgm = {u0 = gt(y,50); u1 = 0.4*lt(x,50)};
/* Graphic output of u and v fields distribution */
k_paintgl when = out width = 300 height = 300 nabs = 100 nord = 100
pgm = {red = u(abs,ord,0,0); grn = u(abs,ord,0,1)/0.8; blu = 0};
/* Text output of a point record */
record when = out x0 = 10 x1 = 10 y0 = 20 y1 = 20 file = “history.dat”;
/* Terminate when all work done */
stop when = end;
/* Diffusion substep for layer 0, layer 2 reserved for Laplacian */
diffstep v0 = 0 v1 = 2 ht = 0.02 hx = 0.4 D = 1;
/* Reaction substep for layers 0:1; Barkley’s variation of FitzHugh-Nagumo kinetics */
euler v0 = 0 v1 = 1 ht = 0.02 ode = fhnbkl par = {a = 0.8 b = 0.01 eps = 0.02};
end;

The main features of the syntax may be seen from the script itself which is intended to be self-explanatory, but nevertheless:

- Comments in the script can be in C style, within /*…*/ or in C++ style, between // and the end of line.
- The script is a sequence of sentences, each concluding with a semicolon, ‘;’.
- Sentences starting with the keyword def declare global variables.
- The sentence starting with the keyword state allocates the computational grid.
- The script finishes with a sentence “end;”.
- Other sentences describe instances of devices comprising the ring. The first keyword in each sentence is the device type; other words describe the parameters determining the specifics of the work of this particular instance of the device.

The particular sentences in the script have the following functions:

- state sentence, preceding any devices, defines and allocates the computational grid. In this case the space domain is a 2D box: the *z*-dimension is not specified so defaults to zmax = 1. The parameter vmax = 3 means there will be three layers in the grid, numbered 0, 1 and 2. As we shall see, layer 0 is reserved for the *u* field, layer 1 for the *v* field, and layer 2 is used for computing and storing the diffusion term.
- k_func, the first device in the script, computes, depending on the current value of the loop counter t, the “flag” global variables that control which of the other devices will or will not work at the current time step iteration. As this device changes values of global variables, it is not allowed to change local field values, hence nowhere = 1 parameter. This instance of k_func works at the beginning of every time iteration, and as a result, variable begin will take the value of 1 at the very first iteration and 0 otherwise; variable out will take value 1 only when the loop counter t is divisible by 10, i.e. at every 10-th iteration, and variable end will become one as soon as the counter t exceeds 1000.
- The second device in the script is another instance of k_func device. Now it computes not the global variables, but the values of the field variables at every point of the space grid, according to the given formula. According to the when = begin parameter, this device works only once, at the very first time step, and its function is to produce initial conditions for the simulation.
- k_paintgl is a graphic output device. It creates an X11 window of 300 × 300 pixels, and at every tenth timestep (according to the parameter when = out), paints using OpenGL a 100 × 100 raster, each element of which will be coloured according to the given formulas: the relative luminosity of the red component is equal to the value in layer 0 (corresponding to the *u* field), for the green component it is equal to the value in layer 1 (corresonding to the *v* field) divided by 0.8, and the blue component always is zero. Note that this colour-coding is similar to the the colour-coding used in Fig 8.
- record device opens for writing a text file history.dat, and at every tenths timestep (according to when = out), will print into the file the values of the grid nodes within the cuboid subdomain defined by the parameters x0 …v1, which makes exactly two values: layer 0 (*u*-field) and layer 1 (*v*-field) values at the point of the grid with integer coordinates (10, 20).
- stop is the device whose function is to interrupt the computations and terminate the program. Naturally this device must be present in the ring unless it is intended that the program run is to be interrupted by the operator. In the presented example, the device works simply when the global variable end takes a nonzero value, which happens after 1000 time steps.
- diffstep is the first of the devices which does “the actual computations” in the sense that it changes the the field variables in the layers of the computational grid according to the differential equations. As could be guessed from its name, it computes the sub-step due to the diffusion term. Specifically, it computes a value of the diffusion term, for the *u*-field stored in layer 0 of the computational grid, using the given values of the diffusion coefficient D and space discretization step hx, places the computed Laplacian into layer 2 reserved for this purpose, and then performs a forward Euler step for the *u*-field for the given value of the time step ht.
- euler is a computational device which performs the forward Euler step for the dynamic fields stored in layers 0 and 1 of the computational grid, with account of the given kinetic model.

#### Script 2: sample.bbs

Listing 2 presents the complete listing of a more non-trivial example of a BeatBox script, sample.bbs. This is the example represented by the “device ring” in Fig 2. Some new syntax features observed in the script include:

- Expression <fhn.par> means inclusion of an ASCII text file with name fhn.par, as part of the script, similar to #include <fhn.par> in C. On this occasion, the file fhn.par contains definitions of the global variables, which are intended to be model parameters shared between many related scripts.
- The declarations of the global variables in the def sentences may specify optional initial values, which are allowed to be defined by arithmetic expressions with previously defined or pre-defined variables.
- Declarations of global variables may appear not only in the very beginning, but throghout the script. The only restriction is that a variable has to be declared before it is used.
- Global variables of type str are string macros. Expansion of a string macro declared as “def str foo bar;” is done using syntax [foo] which will produce bar in place of expansion.
- Overall, the values of the model/simulation parameters are often specified by arithmetic or string expressions rather than literal values; moreover, string macro substitutions are used in the body of a device definition. For instance, since the string macro u is defined as 0, expression u[u] expands to u0, and since string macro 0 is predefined to the sciript name, sample, the expression file = [0].rec expands to file = sample.rec.
- Some of the devices in the script have parameter name. This allows to distinguish between different instances of the same device in the diagnostic messages in the simulation’s standard output and the log file.

**Listing 2:** BeatBox script sample.bbs. A more complicated BeatBox script.

<fhn.par> // model pars are read in from file fhn.par
def str u 0; // u field in 1st layer
def str v 1; // v field in 2nd layer
def str i 2; // diffusion term in 3rd layer
def str b 3; // spatially dependent parameter in 4th layer
def real grad [1]; // its gradient is 1st command-line parameter
// Integer and real stimulation parameters
def int xr 100; def int yr 100; def int zr 100; // reg electrode position, in space steps
def int dr 5; // reg elecrode size, in space steps
def real Amp 3.0; // pulse amplitude
def real Dur 0.1; // pulse duration
def real Del 6.0; // pulse delay
def real Tstart 100.0; // when to switch on the feedback
state geometry = ffr.bbg anisotropy = 1 // the file contains tissue geometry and fibres
vmax = 4; // 2 dyn vars + diffusive current + parameter
def real T;def real begin;def real out;def real end; // real vars control work of some devices
k func name = timing nowhere = 1 pgm = { // this function operates only global variables
T = t*ht; // t is integer time counter; T is real time
begin = eq(t,0); // 1.0 at the very beginning, otherwise 0.0
out = eq(mod(t,100),0); // 1.0 every 100 timesteps, otherwise 0.0
end = ge(T,100.0)}; // 1.0 after 100 ms, otherwise 0.0
// This function operates at every space point but only at the first time step
k func name = IC when = begin pgm = {
u[u] = ifle0(x-25,1.7,-1.7); u[v] = ifle0(y-25,0.7,-0.7) // Cross-field initial conditions
u[b] = bet+grad*(z-0.5*zmax)}; // vertical gradient of parameter
// The feedback
def real signal;def real front; def real Tfront;
reduce operation = max result = signal v0 = [u] v1 = [u] // signal = max of voltage field within given volume
x0 = xr xr1 = xr+dr-1 y0 = yr yr1 = yr+dr-1 z0 = zr zr1 = zr+dr-1; // the values are arithmeitc expressions
k poincare nowhere = 1 sign = 1 // remember T when signal crossed value umid upward
pgm = {front = signal-umid; Tfront = T};
k func name = feedback nowhere = 1 // force lasts Dur ms starting Del ms after crossing
pgm = {force = ht*Amp*ge(T,Tstart)*ge(T,Tfront+Del)*le(T,Tfront+Del+Dur)};
// The computation
diff v0 = [u] v1 = [i] Dpar = D Dtrans = D/4 hx = hx; // anisotropic diffusion
k func name = stim when = force pgm = {u[i] = u[i]+force}; // this applies everywhere, only when force is nonzero
euler v0 = [u] v1 = [v] ht = ht ode = fhncub // cubic FitzHugh-Nagumo kinetics
par = {eps = eps bet = @[b] gam = gam Iu = @[i]}; // varied beta and current as calculated before
// Output
ppmout when = out file = “[0]/%04d.ppm” mode = “w” // every 100 timesteps:/
r = [u] r0 = umin r1 = umax // value-discretized
g = [v] g0 = vmin g1 = vmax // output for subsequent
b = [i] b0 = 0 b1 = 255; // visualization
k print when = always file = stdout list = {T; force; signal}; // to monitor work of the feedback
record when = end file = [0].rec when = end v0 = 0 v1 = 1; // ascii dump of all field values in the end of run
stop when = end;
end;

The particular devices used in the script, in order of occurrence, have the following functions:

- state sentence defines a complex geometry, read from the file ffr.bbg. Further, the diffusion will be anisotropic (anisotropy = 1), with the fiber directions read from the same file, ffr.bbg.
- The first instance of k_func, with the name timing computes the “flag” global variables that control which of the other devices will or will not work at the current time iteration. Besides, it also computes the global variable T, which is to contain the model time *t*, as opposed to integer t which is the loop counter.
- The second instance of k_func, with the name IC computes the initial conditions. This time it computes not only *u* and *v* field allocated in layers [u] and [v], but also the values of layer [b], i.e. layer 3. The latter will contain not a dynamic variable, but values of the model kinetics parameter *b*, which in this simulation has a spatial gradient in the *z* direction.
- reduce is a device that computes the value of the global variable signal based on the current state of one or more of the fields represented in the layers of the computational grid; in this case it uses just the layer [u]. Here the reduce device emulates the work of a registration electrode, which measures the maximal value (parameter operation = max) of the “transmembrane voltage” in a particular small volume in the space grid, of the size dr×dr×dr, cornered at (xr,yr,zr). This measurement will be used as a feedback signal to control the electrical excitation in a putative low-voltage defibrillation protocol.
- k_poincare is a device that implements the idea of a Poincare cross-section from the dynamical systems theory. It operates only global variables, hence does not have any domain associated with it, thus nowhere = 1. Here the k_poincare device checks whether at the current iteration the signal, represented by variable signal measured by the previous reduce device, has crossed a given threshold value umid in the required direction, defined by sign = 1, which means upwards. If that has happened, then a certain flag (the global variable front) is “raised” (gets the values of 1), and the time, represented by T, when this happened is remembered in another global variable, Tfront.
- The next instance of device k_func, with the name feedback, works with global variables: it computes, using the given formula, the value of the variable force that defines the defibrillating electric field, depending on the time that has passed since the event registered by the k_poincare device at time Tfront, so that T is between Tfront+Del and Tfront+Del+Dur, where the variable Del is the delay of the stimulus compared to the front registration moment, and Dur is its duration.
- diff is a computational device, which computes the diffusion term, i.e. the value of the Laplacian of the field represented by layer [u] of the computational grid, and records the result into layer [i] of the grid. Since the geometry defined by the state sentence is anisotropic, this diff device requires two diffusion coefficients, Dtrans and Dpar for conductivity across and along the fibers respectively.
- The next instance of k_func device with the name stim is “local”, i.e. it works on the computational grid: computes the action of the defibrillating electrical field (computed by the previous “feedback” instance of k_func device) onto the excitable cells. The action is simply adding the previously computed force to the layer [i], which already contains the value of the diffusion term.
- euler here performs the time step for the dynamic fields in layers [u]…[v] represented in the computational grid, with the account of the given kinetic model fhncub, which is classical FitzHugh-Nagumo with cubic nonlinearity Eq (4). The new features here are the definitions of the “extra current parameter” Iu and of the parameter bet using symbol @. The meaning of this symbol is that the values of the parameter bet are taken from the layer [b], that is layer 3, and the values of the parameter Iu are taken from the layer [i] defined as 2. This is a typical simplified mono-domain description of the action of the external electrical current, which in this simulation is assumed to be purely time-dependent, i.e. applied uniformly throughout the tissue.
- ppmout output device works once in 100 steps (according to the computation of the out variable by the timing instance of k_func) and produces an output to a file in ppm format, where each byte represents a value of one element of the grid, from up to three selected layers of the grid, discretized to the 0…255 scale. This ppm image format could be converted to other popular and less space-consuming formats either by postprocessing or on-the-fly (not done in the current sample.bbs script). The name of the ppm output file contains the % symbol, the effect of which is that it is the format of a C sprintf call, the first field argument of which is the ordinal number of the device’s instant call. That is, this device will produce files with names sample/0000.ppm, sample/0001.ppm, sample/0002.ppm etc.
- k_print is a more straightforward output device: each time it is called (here, at the every time step), it adds to the output file a plain-text record of the values of the global variables involved in the feedback control of the defibrillating stimuli. It is similar to the record device in the ez.bbs above, except it prints global variables rather than grid node values.
- record is the last output device in this script. Its use in this script is different from that in ez.bbs, in that it prints the values of the field layers 0 and 1 in all internal points of the grid. This device works only at the last time step of the simulation, so that the output file can be used as an initial condition if continuation of the present simulation is required.
- stop is the last device in this script and its syntax and semantics is the same as in ez.bbs.

## Supporting information

S1 Code BeatBox software. Zip-file S1_code.zip containing distribution of the BeatBox, version beatbox-public-v1.7.982, including source code, configuration and makefiles, documentation, sample scripts etc.
(ZIP)
