# Supplementary material for: BeatBox—HPC simulation environment for biophysically and anatomically realistic cardiac electrophysiology
Source: PLoS One. 2017 May 3;12(5):e0172292. doi: 10.1371/journal.pone.0172292 (PMC5415003; doi:10.1371/journal.pone.0172292)
Supplement: S1 Code — Zip-file S1_code.zip containing distribution of the BeatBox, version beatbox-public-v1.7.982, including source code, configuration and makefiles, documentation, sample scripts etc. (ZIP) [file pone.0172292.s001.zip › beatbox-public-v1.7.982.zip!/beatbox-heart-beatbox-public-05c855e/data/scripts/sequential/TenTusscherPanfilov_modelS/ttp06/report.pdf]

Cardiac physiology simulator benchmark for BeatBox

V. N. Biktashev

Results of the benchmark proposed in "Verification of cardiac tissue electrophysiology simulators using an N-version benchmark" by S. A. Niederer et al. *Phil. Trans. R. Soc. A* 2011 **369**, 331-4351, 2011, as performed by BeatBox using benchmark.bbs in this directory. About the post-processing of the results, see Makefile.

For figure 2:

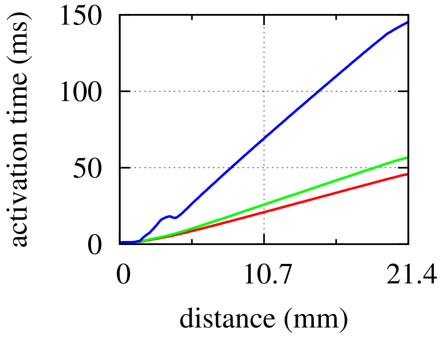

For figure 3 (colours selected arbitrary so do not match paper):

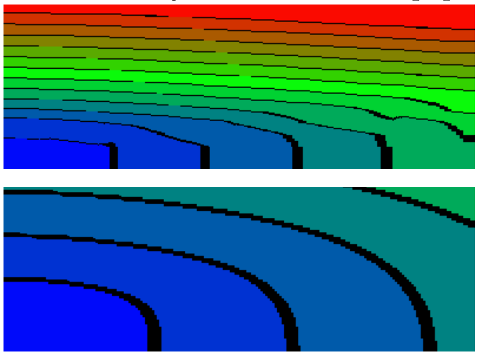

For the table in the Supplement (note that fig. 4 is only visualization of the same):

Δx, Δt	P_1	P_2	P_3	P_4	P_5	P_6	P_7	P_8	C
0.1 , 0.005	1.22382	32.9642	9.91335	34.3364	31.313	44.6487	32.9291	45.6801	20.9209
0.1 , 0.01	1.22721	33.3272	9.98596	34.7159	31.5546	45.1389	33.2029	46.1846	21.1429
0.2 , 0.005	1.22434	35.4602	14.0759	37.3672	42.583	55.4801	44.7658	56.677	25.242
0.2 , 0.01	1.22771	35.7915	14.1257	37.7231	42.7321	55.935	44.9592	57.1537	25.4606
0.2 , 0.05	1.25431	38.3961	14.5155	40.5161	43.9067	59.5074	46.47	60.8908	27.1759
0.5 , 0.005	1.22551	46.8866	46.4161	64.3489	133.489	143.851	135.663	145.399	69.1255
0.5 , 0.01	1.22887	47.0448	46.4865	64.6528	133.63	144.184	135.831	145.752	69.3295
0.5 , 0.05	1.25543	48.2774	47.0542	67.0577	134.755	146.829	137.152	148.555	70.9643

The results are similar with other methods using finite differences.
