# Supplementary material for: BeatBox—HPC simulation environment for biophysically and anatomically realistic cardiac electrophysiology
Source: PLoS One. 2017 May 3;12(5):e0172292. doi: 10.1371/journal.pone.0172292 (PMC5415003; doi:10.1371/journal.pone.0172292)

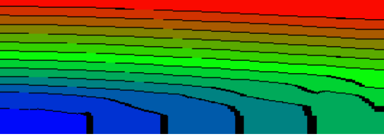

Supplement: S1 Code — Zip-file S1_code.zip containing distribution of the BeatBox, version beatbox-public-v1.7.982, including source code, configuration and makefiles, documentation, sample scripts etc. (ZIP) [file pone.0172292.s001.zip › beatbox-public-v1.7.982.zip!/beatbox-heart-beatbox-public-05c855e/data/scripts/sequential/TenTusscherPanfilov_modelS/ttp06/upper.pdf]
