# Supplementary material for: BeatBox—HPC simulation environment for biophysically and anatomically realistic cardiac electrophysiology
Source: PLoS One. 2017 May 3;12(5):e0172292. doi: 10.1371/journal.pone.0172292 (PMC5415003; doi:10.1371/journal.pone.0172292)
Supplement: S1 Code — Zip-file S1_code.zip containing distribution of the BeatBox, version beatbox-public-v1.7.982, including source code, configuration and makefiles, documentation, sample scripts etc. (ZIP) [file pone.0172292.s001.zip › beatbox-public-v1.7.982.zip!/beatbox-heart-beatbox-public-05c855e/doc/bbg_howto.html]

Beatbox Quick Start Guide


# Beatbox: Convert MRI Based Geometry Data Into Beatbox BBG Format

---


## Table of Contents

---


# Who should read this document

This part of the documentation is for users who wish to use their MRI
based cardiac anatomical geometry data in Beatbox for
simulations. This manual provides an easy to read and follow document
which you can follow to convert your geometries into a suitable format
for Beatbox. This document assumes you have some programming
experience, and provides an example C program that can be used to make
Beatbox geometry (BBG) files.

# BBG Geometry File Format

Beatbox uses a special file format to describe its geometries, called
the BBG file format. By representing a geometric mesh in a file,
users may exchange meshes as easily as they can do with simulation
scripts. The file format used has been designed to reduce redundancy,
and thus any conflict between redundant values. For example, rather
than requiring users, or geometry file authors, to specify the
dimensions of the mesh, the dimensions are calculated from the
coordinates of the points in the geometry file.

A Beatbox geometry file has uses a "`.bbg`" file
extension. This is a data file written using plain ASCII text where
each line in the file either has four numbers or seven numbers,
separated by commas as schematically shown below:

```
integer1, integer2, integer3, tissuetype, fibre1, fibre2, fibre3
```

The `integer1`, `integer2`,
and `integer3` values are integers for x, y and z
coordinates of a finite difference (FD)
node. The `tissuetype` describes its tissue type where: 0
signifies no tissue and a non-zero integer signifies that the FD node
has some tissue type. The `fibre1`, `fibre2`,
and `fibre3` consist of three real numberss representing
the fibre orientation x, y, and z components (or direction cosines)
repectively of the fibre direction at the node with
coordinate `(integer1, integer2, integer3)`. The
coordinates and fibre orientation information of tissue type 0 is not
mandatory in a bbg file.

In the following section, we discuss how to construct a bbg geometry
file, using realistic anatomical data.

# Converting MRI based Data into Beatbox BBG Format

Anatomical cardiac models based on MRI images are constructed
routinely for use in cardiac simulations. These analysed MRI data are
usually provided by various authors as multiple text files,
combinations of which describe the tissue types in the model, and
fibre orientation.

In this example, the following relevant files are given:

- `geometry.txt`: This file describes the geometry on the
  regular FD grid. Since the grid is regular, the x,y,z locations are
  not given explicitly.
- `eigenvector1_x.txt`: This file describes the x component
  of the fibre direction on the regular FD grid. Since the grid is
  regular, the x,y,z locations are not given explicitly.
- `eigenvector1_y.txt`: This file describes the x component
  of the fibre direction on the regular FD grid. Since the grid is
  regular, the x,y,z locations are not given explicitly.
- `eigenvector1_z.txt`: This file describes the x component
  of the fibre direction on the regular FD grid. Since the grid is
  regular, the x,y,z locations are not given explicitly.
- `geometry.vtk`: A file provided by original geometry
  authors where size of the 3D box containing the cardiac geometry is
  defined.

In a programming environment of your choice, first you need to open
these files for reading in the data. In the C programming language,
this can be done as follows:

```
/* Read fromthese files */
geometry = fopen("geometry.txt","r");
evecx = fopen("eigenvector1_x.txt","r");
evecy = fopen("eigenvector1_y.txt","r");
evecz = fopen("eigenvector1_z.txt","r");

/* Write to this file */
bbg = fopen("rabbit2012.bbg","w");
```

Once this has been done, the data need to be read in.

In the C programming language, consecutive values in a data file can
be read using the `fscanf` statement inside a loop. As the
number of entries in all the above files is the same, entries at the
same FD node location are physically located in the same location in
each of the data files. Therefore, they can be read in
simultaneously. Further, upon being read in, the data can be
immediately written into the BBG file. This part of the C program is
as follows:

```
/*
Loops for x, y, and z to read in from input files,
and to write to the .bbg file.
*/
for(z=1;z<=NUMZ;z++)
for(y=1;y<=NUMY;y++)
for(x=1;x<=NUMX;x++){
	fscanf(geometry,"%lf",&temptissue);
	tissue = (int)temptissue;
	fscanf(evecx,"%lf",&tempex); ex = tempex;
	fscanf(evecy,"%lf",&tempey); ey = tempey;
	fscanf(evecz,"%lf",&tempez); ez = tempez;
	modulus = ex*ex + ey*ey + ez*ez;

	if(tissue>0){
	/* Write to stdout to see the data on screen, and to see if the principle eigenvector is normalised */
	printf("%d %d %d %d %f %f %f %f\n",x,y,z,tissue,ex,ey,ez,modulus);
	/* Write the data to the bbg file in CSV format */
	fprintf(bbg,"%d, %d, %d, %d, %f, %f, %f\n",x,y,z,tissue,ex,ey,ez);
	}
}

fclose(geometry);
fclose(evecx);
fclose(evecy);
fclose(evecz);
fclose(bbg);
```

The first few lines of the bbg file will look like something similar
to:

```
49, 36, 4, 1, -0.038211, 0.914133, -0.403608
50, 36, 4, 1, 0.007899, 0.930515, -0.366167
51, 36, 4, 1, 0.049010, 0.925612, -0.375286
45, 37, 4, 1, -0.536122, 0.816387, -0.214674
46, 37, 4, 1, -0.537820, 0.774058, -0.334039
.
.
.
```

If you would like to model anisotropy, you need to set the ansiotropy
parameter to 1 in your BBS scripts. This only works when a geometric
mesh, like the one used above, is specified. When the anisotropy
feature is on, the behaviour and required parameters of some devices
may change.

# BBG Geometries Distributed With Beatbox

The Beatbox package incorporates, without gaurentee, a realistic small
animal and human anatomical geometries to allow new users to start
doing 3D simulations.

## ffr.bbg

This is a three-dimensional FD mesh describing rabbit ventricles,
measured by Vetter and McCulloch [1998] and discretised for finite
differences by Fenton et al. [2005]. The containing box is 117 × 107 ×
129 (1,614,951 points), with 470,324 tissue points. Tissue shape and
fibre directions are obtained by NMR. The spatial resolution is 0.1 mm
in each direction. A bbs script for using `ffr.bbg` with
FHN kinetics is as follows (`fhn_ffr.bbs`):

```
def real ht  0.001;
def real hx  0.1;
def real D   1.0; 
def real bet 0.71;
def real eps 0.30;
def real gam 0.50;
def str u 0;
def str v 1;
def str i 2; 
def real umin -2.0; def real umax 2.0;def real umid 0.0;
def real vmin -1.0; def real vmax 1.5;def real vmid 0.5;

def int neqn [i];

state
	geometry=ffr.bbg
	vmax=[i]+1
	normaliseVectors=1
	anisotropy=1
;

/* Declare global timing variables. In general: 
 * begin is for when the simulation begins, 
 * often, seldom, once, paceout are for output rates (see the k_func device), 
 * end is often used in the examples to set up the stopping criterion using stop device, 
 * stop is the variable used to indicate time for end of simulation not using the stop device. 
 */ 
def real begin;
def real end;

/* Assign values to the timing parameters using Beatbox functions for 
 * greater than (ge), less than (le), modulus (mod), etc. 
 */ 
k_func name=timing nowhere=1 pgm={
	begin = eq(t,0);
	end   = ge(t,100);
};

// Stimulus
/* Apply stimulus to tissue specified by x0,x1,y0,y1,z0,z1. If 
 * any of these are not specified, then the max or min values are presumed 
 */ 
k_func when=begin x0=18 x1=22 y0=18 y1=22 z0=18 z1=22 pgm={u[V]=Vmax;};

// The computation
/* Compute the Laplacian. */ 
diff v0=[V] v1=[i] Dpar=D Dtrans=D/4 hx=hx;
/* Computation of 1 time step update. 
 * In spatial simulations, the Laplacian is stored at Iu=@vmax. 
 */ 
euler v0=[V] v1=neqn-1 ht=ht ode=fhncubpar rest=0 par={ht=ht Iu=@[i]};

/* PPMOUT gives 2D/3D ppm file format output at a rate specified by when= parameter. */ 
ppmout 
	file="out/[0]_%04d.ppm" mode="w"
	r=[V]		r0=Vmin		r1=Vmax
	g=[xi] 		g0=0		g1=1
	b=[dvdt] 	b0=0		b1=1
;

// Record device outputs values of variables at the specified locations.
record
	x0=35 x1=35
	y0=35 y1=35
	z0=35 z1=35
	v0=[V] v1=neqn-1
	filehead="Fibre Orientation Test"
	file=out/[0].rec mode=append
;

/* Dump all state variables from all nodes into a binary file for future use. */ 
dump append=0 file=out/[0].dmp;

/* Stopping criterion. This is mandatory.*/ 
stop when=end;

end;
```

## rabbit2012.bbg

This is a three-dimensional FD mesh describing rabbit ventricles,
measured and discretised for finite differences by Gilbert et
al. [YEAR]. The containing box is 115 × 110 × 110. Tissue shape and
fibre directions obtained by DT-MRI. The spatial resolution is 0.1 mm
in each direction. To use `rabbit2012.bbg` data with FHN
kinetics, replace `ffr.bbg`
with `rabbit2012.bbg` in the script given in the previous
subsection.

## humanatrium.bbg

This is a three-dimensional FD mesh describing rabbit ventricles,
measured and discretised for finite differences by Seemann et
al. [2006]. Containing box is 235 × 269 × 298. Tissue shape and fibre
directions obtained by DT-MRI. The spatial resolution is 0.33 mm in
each direction. To use `humanatrium.bbg` data with FHN
kinetics, replace `ffr.bbg`
with `rabbit2012.bbg` in the script given in the previous
subsection. The space step should be revised to 0.33 mm. The full
script is as follows:

```
def real ht  0.001;
def real hx  0.33;
def real D   1.0; 
def real bet 0.71;
def real eps 0.30;
def real gam 0.50;
def str u 0;
def str v 1;
def str i 2; 
def real umin -2.0; def real umax 2.0;def real umid 0.0;
def real vmin -1.0; def real vmax 1.5;def real vmid 0.5;

def int neqn [i];

state
	geometry=humanatrium.bbg
	vmax=[i]+1
	normaliseVectors=1
	anisotropy=1
;

/* Declare global timing variables. In general: 
 * begin is for when the simulation begins, 
 * often, seldom, once, paceout are for output rates (see the k_func device), 
 * end is often used in the examples to set up the stopping criterion using stop device, 
 * stop is the variable used to indicate time for end of simulation not using the stop device. 
 */ 
def real begin;
def real end;

/* Assign values to the timing parameters using Beatbox functions for 
 * greater than (ge), less than (le), modulus (mod), etc. 
 */ 
k_func name=timing nowhere=1 pgm={
	begin = eq(t,0);
	end   = ge(t,100);
};

// Stimulus
/* Apply stimulus to tissue specified by x0,x1,y0,y1,z0,z1. If 
 * any of these are not specified, then the max or min values are presumed 
 */ 
k_func when=begin x0=18 x1=22 y0=18 y1=22 z0=18 z1=22 pgm={u[V]=Vmax;};

// The computation
/* Compute the Laplacian. */ 
diff v0=[V] v1=[i] Dpar=D Dtrans=D/4 hx=hx;
/* Computation of 1 time step update. 
 * In spatial simulations, the Laplacian is stored at Iu=@vmax. 
 */ 
euler v0=[V] v1=neqn-1 ht=ht ode=fhncubpar rest=0 par={ht=ht Iu=@[i]};

/* PPMOUT gives 2D/3D ppm file format output at a rate specified by when= parameter. */ 
ppmout 
	file="out/[0]_%04d.ppm" mode="w"
	r=[V]		r0=Vmin		r1=Vmax
	g=[xi] 		g0=0		g1=1
	b=[dvdt] 	b0=0		b1=1
;

// Record device outputs values of variables at the specified locations.
record
	x0=35 x1=35
	y0=35 y1=35
	z0=35 z1=35
	v0=[V] v1=neqn-1
	filehead="Fibre Orientation Test"
	file=out/[0].rec mode=append
;

/* Dump all state variables from all nodes into a binary file for future use. */ 
dump append=0 file=out/[0].dmp;

/* Stopping criterion. This is mandatory.*/ 
stop when=end;

end;
```


---

**Copyright © (2010-2013) Vadim Biktashev, Alexander
Karpov,Irina Biktasheva, Ross McFarlane;  
Copyright © (2012-2013) Sanjay Kharche.  
This file is part of the Beatbox package.** 
Sanjay.Kharche@liverpool.ac.uk

Last modified: February 22 2013
