# Supplementary material for: BeatBox—HPC simulation environment for biophysically and anatomically realistic cardiac electrophysiology
Source: PLoS One. 2017 May 3;12(5):e0172292. doi: 10.1371/journal.pone.0172292 (PMC5415003; doi:10.1371/journal.pone.0172292)
Supplement: S1 Code — Zip-file S1_code.zip containing distribution of the BeatBox, version beatbox-public-v1.7.982, including source code, configuration and makefiles, documentation, sample scripts etc. (ZIP) [file pone.0172292.s001.zip › beatbox-public-v1.7.982.zip!/beatbox-heart-beatbox-public-05c855e/doc/beatbox.html]

Beatbox Documentation


# The Beatbox Cardiac Simulation Software: A User's Guide

---


## Table of Contents

---


# Introduction

Beatbox is a program that could be compiled for sequential or parallel
(MPI) use. Currently it has a small collection of cell models,
including FitzHugh-Nagumo, Luo-Rudy I, Courtemanche et al. human
atrial (this list will be expanding) and solves reaction diffusion
equations with these cell models to simulate wave propagation in
idealised and realistic cardiac tissue models. Currently the solvers
use finite differencing with regular spatial grid and forward Euler in
time with or without operator splitting. Some semi-implicit solvers
are now in test exploitation and bidomain solvers are under
development.

Figure 1: Conceptual view of a ring of devices.

A special feature of Beatbox is its flexibility in setting various
experimental protocols, without the need to recompile the package. A
simulation is set up by constructing an input script that spatial
model (1D, 2D, box, or geometry) details, and "devices", which perform
computations, input/output, and control functions. The simulation
script is typically called `filename.bbs`, where the
conventional extension `bbs` stands for "beatbox script",
and `filename` can be anything you like. The "devices" are
chained together in a ring, with an integer counter `t`,
usually corresponding to the time steps in the simulation. The concept
of the ring of devices is illustrated in Figure 1.
In this example,

`k_func`
:   (the first instance) works as a control device that defines when
    other devices will be active: the second `k_func` at the
    start of the computations, `ppmout` every so often, and
    `stop` in the end.

`k_func`
:   (the second instance) works as a computational device defining the
    initial conditions,

`diff`
:   is a computational device that performs diffusion substep in the
    operator-splitting time step,

`euler`
:   ... performs reaction substep in the operator-splitting time step,

`ppmout`
:   is an output device that writes rounded-up simulation results to the
    disk for subsequent visualization,

`stop`
:   is a control device that terminates the run.

Each device has a set of parameters, specific for it, e.g. the list of
parameters for `k_func` is different from that for
`diff` (but there are some "universal" and some "typical"
parameters, such as their control variables or the mesh domain on
which the device operates). A simulation run can use more than one
instance of the given device, each of which may appear anywhere in the
ring; in this example `k_func` has two instances appearing
one after the other. All instances of the same device will have the same list of
parameters, but the values of parameters are set completely indepently
in different instances.

The function of the input script is to define the computational grid
and to describe all the devices for the given run together with their
parameters.

A simulation will thus typically involve:

1. The input script being read and the simulation initialised:
   - The simulation grid is created with the specified dimensions, and if
     realistic geometry is used, then the relevant subset of the
     simulation grid and the diffusion tensor field are defined,
   - Devices are added to the ring of devices with the specified parameters.
2. The simulation is run:
   - Each device in the ring is called in turn to perform its part of the
     simulation task, provided it is commanded to be active at this step by
     the control device(s),
   - Repeat until the stop condition is met, or an error occurs.
3. The simulation is terminated.

In this distribution, there are some example scripts provided, which
illustrate the format of the input scripts and use of the devices.
A more formal description of the bbs script language and of the devices
available follow below. A certain stage of Beatbox development
is reflected in
Ross McFarlane, High-Performance Computing for Computational Biology
of the Heart, PhD Dissertation, October 2010,
see Chapters 2 and 3 in particular.

# Setting-up Beatbox

## Prerequisites

The developers tried to make Beatbox self-contained and reduce the
number of dependencies to an absolute minimum. You will require:

- Certain `autotools`, notably `autoconf`
  and `automake`, to automatically configure the package
  for your computer. Pre-configured and pre-compiled versions will be
  provided in due course, when demand is established.
- A C compiler, ideally gcc.
- A suitable MPI library, preferably
  mpich2
  if you want to run in parallel.
- The standard X11 libraries if you want to have run-time
  visualization (currently only available in sequential mode).
- Some of the examples use utilities from
  netpbm
  both for run-time image conversion and for post-processing.

## Getting Beatbox

The recommended way to obtain BeatBox is to download the most recent
official release from the BeatBox home page,

- http://empslocal.ex.ac.uk/people/staff/vnb262/software/BeatBox/

An alternative which may be more appropriate for beta-testers, is to
obtain the most recent version of BeatBox code from its SVN
repository, say by issuing the following commands:

```
mkdir ~/beatbox
cd ~/beatbox
svn co --username=anonymous --password=beatbox https://beatbox-trac.epcc.ed.ac.uk/svn/trunk/ ./
```

Note that this will be a read-only copy of the repository, i.e. you
will not be able to check any modifications back in. If you would like
to become a developer please get in touch with the project team.

## Installation instructions

### Compiling and installing Beatbox

Assuming that all the components mentioned above are available and in
place, the following sequence of commannds

```
autoreconf -fi
export CC=mpicc
./configure CFLAGS="-g -O0" --prefix=$HOME
make
make install
```

will compile two versions of the program, `Beatbox`
(parallel) and `Beatbox_SEQ` (sequential), and install them
in `$HOME/bin/`. A shell script
`bbx_compile_local.sh` is provided in the root distribution
directory, to facilitate the
installation.

You may wish to change the install location specified in this file:
current setting `--prefix=$HOME` means that the binaries
will be installed in `$HOME/bin/`. This is reasonable in
the assumption that that directory exists and is in your
`$PATH`; modify it as appropriate if you prefer to keep
your binaries elsewhere.

The options `CFLAGS="-g -O0"` mean that the code will be
prepared for debugging and not optimized; for "production runs" this
can be omitted and replaced by `CFLAGS="-O3"`.

If the script, `configure` or `Makefile` cannot
find the include and library files, the paths may have to be
explicitly incorporated into `src/Makefile.am` and then go
through the whole procedure starting from the `autoreconf
-fi` step.

### Compiling and installing Beatbox on HECToR

HECToR (www.hector.ac.uk) is the
national UK supercomputer service. HECToR is a Cray XE6 based
system. In order to install on HECToR, we need to cross compile for
back end nodes and make correct settings,
so the process is a little bit different
(script `bbx_compile_hector.sh`):

```
# Create the configure and install scripts
autoreconf -fi

# Load the PGI compiler module
module load pgi

# Tell the configure command what compiler we need to use.
export CC=cc

# Need to explicitly tell the Cray system what X libraries to use
export LIBS="-lm -lX11 -lxcb -lxcb-xlib -ldl -lXau"

# Run configure - note that it will install in $HOME/bin
./configure --prefix=$HOME

# Now make the code
make

# Install the code to the directories specified by the prefix above.
make install
```

Again, change the
`prefix` flag if you would like it to install
elsewhere.

## Running Beatbox

### General Instructions

The sequential version of the program may be run by using:

```
   Beatbox_SEQ [<options>] [--] <input_script> [<arguments> | <options> | -- ]
```

That is: the executable name (with the full path if necessary),
followed by the input
script and the arguments, if any, with options interpspersed anywhere after
the executable name. Similar to many Unix programs, options are those
words that start with a minus '`-`', but a double minus
'`--`' signals an end of all options so a word starting
with `-` after that is interpreted as input script name or
argument.

Correspondingly, assuming that you have mpich2,
the parallel version on a local computer may be run using

```
   mpirun -np <num_procs> Beatbox [<options>] [--] <input_script> [<arguments> | <options> | -- ]
```

The parsing of the input script and the simulations are commented by
printing messages to the standard output and duplicating them into a
log file.

The possible options are:

- `-append`: append to the log file. The default is to
  overwrite it
- `-debug <filename>` : this produces a debug
  file. If `<filename>` is  `stdout` 
  or  `stderr`, the debugging information is printed to
  standard output or standard error rather than a disk file of that
  name. The default is no debug printouts.- `-log <filename>` : this set the name for the log
    file. The default is the name of the input script, stripped of the
    extension `.bbs` if there is one, and appended
    extension `.log`.
  - `-mute`: no default output to stdout. The default is
    to copy a (possibly shorter) version of the log file information to
    the standard output.
  - `-nograph`: do not use run-time graphics. The default
    is to use it if there is at least one device that has something to
    show. This is only effective with the sequential version as the
    run-time graphics are not yet implemented for the parallel
    version.
  - `-profile`: measure timing for each device in the ring
    and output it in the end. The default is not to measure and not to
    output that information.
  - `-verbose`: this makes messages in the log file more
    verbose. The default brief output does not include e.g. the default
    values of the device parameters.
  - `-decomp [1|2|<NX>x<NY>x<NZ>]` (in
    parallel mode only) : the choice of decomposition algorithm, namely
    1 due to RMF, 2 due to SRK and
    `<NX>x<NY>x<NZ>]` an explicit formula,
    where `NX,NY,NZ` are numbers of partitionings along the
    x, y and z axes respectively. Note that the explicit formula be
    given as one word consisting of exactly three positive integer
    numbers separated by two letters 'x'. In full-box simulations, the
    total number of subdomains `NX*NY*NZ` should not exceed
    the number of processes allocated for the job by the `-np
    <num_procs>` option of the `mpirun`
    command. If using tissue geometry (see below) then the
    number of subdomains can be more than the number of processes, as
    long as the number of processes is sufficient to cover all non-empty
    subdomains, i.e. subdomains containing any tissue points. Default is
    1, for automatic partitioning by RMF algorithm. Currently, neither
    of the automatic partitioning algorithms takes into account whether
    any subdomains are empty, so efficient use of "thin" geometries at
    high-degree parallelization requires the explicit decomposition formula.

CAVEAT:
The options `-debug`, `-log` and `-decomp`
gobble the next word for the name of the debug or log file; failure to
appreciate that is a common error leading to weird behaviour. This should
be addressed one day, perhaps by making them like
`-debug=<filename>` instead.

The arguments can be used in the input script, where they appear
as pre-defined string macros, see below. The parsing is usual for
the unix shells, that is each word makes a separate argument, except
when quotes are used.

### Beatbox on HECToR

This subsection gives a brief description on how to conduct
simulations on HECToR or a
similar HPC facility. Before starting any simulations, a
certain familiarity with HECToR architecture, compilers, modules, and
general terms of usage can be gained from
the HECToR
User Guide.

On HECToR compilation is done through a set of compilation wrappers,
`cc` will always correspond to the actual compilation
suite being used. The default version is for the Cray
compilers. Other compiler suites may be used (by loading and unloading
the appropriate modules), but will still use the same wrapper. The
compilation is done for the work nodes - thus we are cross compiling.

HECToR manages production as well as (parallel and serial) debugging
runs by using the
PBSpro queuing system. A PBS script consists of a set of PBS
commands, and other generic shell commands. A sample PBS script that
can be used to run Beatbox jobs is shown below. The commands are
explained in the comments (text following a `##` on any
line) - a line that starts with `#PBS` denotes a PBS
command. The following script was used to run the 3D atrium simulation
and is called `Beatboxrun64.sh`. Its contents are:

```
#!/bin/bash --login

## A PBS command starts with #PBS.
## Start by specifying a name for the job.

#PBS -N Beatbox64

## Specify number of cores requested.

#PBS -l mppwidth=64

## Hector is composed of "nodes", each of which consist of 
## two 16 cores processors - thus one has 32 cores available in a node.
## The number of cores per node controls memory available to the job.
## Use of all cores in a node (assuming mppwidth is more than 32) 
## optimises the use AU charged to your account. 

#PBS -l mppnppn=32

## This is the amount of time requested for the simulation.

#PBS -l walltime=3:00:00

## A project code has to be provided, otherwise the job is usually
## not accepted by a queue.

#PBS -A e203

## Assume this PBS script lies in the same directory as the executable
## file. Note to be seen by the back end nodes this must lie in one of
## the work directories. Change directories to where the job was launched.
## You must make sure that you copied the Beatbox executable, the 
## humanAtrium.bbs and humanAtrium.bbg scripts to this directory for this
## to work.
cd $PBS_O_WORKDIR

## Run the job
## n is the total number of processes
## N is the number of processes per node
## Launch the parallel job using aprun. The stdout is called 
## Beatbox64.o_job_number, and stderr is called Beatbox64.e_job_number
aprun -n 64 -N 32 ./Beatbox humanAtrium_start_crn.bbs
```

Before submitting any job using such a submission script, it may be
worthwhile to run a check on the script using the HECToR provided
`checkScript`:

```
   checkScript Beatbox64.sh
```

This will indicate if there are any errors in the script so you can
fix these before you submit it to the PBS system. The production job
is then submitted to a parallel queue using:

```
  qsub Beatbox64.sh
```

Further options are also available, and can be seen in the user
guide. Similarly to production, parallel debugging jobs must also be
submitted to the queue with the following command added:

```
#PBS -v DISPLAY
```

and the appropriate debugger binary name preceding
the `aprun`. For example, the `TotalView`
parallel debugger can be invoked by:

```
  totalview aprun -a -b -a xt -n 64 -N 32 /work/.../myprog.x
```

The detailed submission script for debugging jobs can be found on the
relevant HECToR pages.

The status of submitted jobs can be checked using the command:

```
  qstat -u your_hector_user_name
```

and the job is deleted using:

```
  qdel job_number
```

# Beatbox Scripting Guide

A Beatbox simulation is defined by the user in the form of an input
script ("Beatbox script" or "bbs script"). A script is a plain text
file that specifies the computational grid, the devices that are to be
used to do the calculations and input/output, and their parameters.
Any script should be able to run on any of Beatbox’s supported
hardware platforms; if a sequential-only device is used in a script
submitted to parallel execution, the warning message will be output
but the script will run nonetheless.

This section introduces Beatbox scripts by first describing the
scripting language, before discussing common applications. Unlike
interpreted scripting languages such as PHP or Python, Beatbox scripts
are not run during the simulation. The script is read once to build
the simulation, after which the script code does no longer define the
flow of execution, so can be modified or removed with no consequences
for the current run.

Similar to other programming languages, Beatbox scripts allow the user
to define variables and macros, include external code and make calls
to the operating system. The Beatbox scripting language also provides
a small library of arithmetic and logical functions. The user may also
improve the legibility of their code, or disable portions of the code
using comments. Each of these features is discussed below.

## For the impatient

If you prefer to learn by example rather than go through formal
definitions, you may wish to try and jump ahead to
a simple working example and then only if and when
necessary go back to check out those formal definitions, or go forward
to further, more sophisticated examples.

## The Data

Any Beatbox run operates with two sorts of data. One is the
computational grid, which is effectively a four-dimensional array of
real numbers (of the precision specified at compile time), which is
the object of operation of the computational devices. The four
dimensions are the three spatial dimensions `x,y,z` and the
"component" dimension `v`. Slices in the component
dimension are called "layers".

Associated with the computational grid
could be the geometry array (if complex geometry option is on), which
has the same shape as the main grid, and determines which points in it
belong to the computational domain and which are "void", and (if
anisotropy option is on) contains information about fiber directions.

The other are the arithmetic variables and string macros. The
arithmetic variables can be integer or real (with the precision
defined at compile time), their names following C convention. The
input script interpreter contains a built-in interpreter of arithmetic
experession, so parameters of a device can be specified as arithmetic
expressions (possibly depending on arithmetic variables) rather than
specific values. The script is interpreted from top to bottom, so any
arithmetic variable will exist and have a valid value of it was
defined and assigned the value above the point at which it is
used. The exceptions are the pre-defined variables, such
as integer `t` containing the counter of the device ring
loop, and real `pi` containing the number pi.

The string macros have the form of a string of characters
between `[` and `]`.
The strings of characers making the names of the macros also have to
obey the C rules. The exception is string macros
`[1]`, `[2]`, `[3]` ..., which cannot
be defined within the script, and which contain the values of the
command line arguments (see above). There some more pre-defined string
macros, e.g. names of colours used in graphical devices. The values
of ordinary string macros are assigned at the moment of their
definition. Whenever they are used in the script, their values are
simply substituted in place of `[...]` and the result is
interpreted as if it was part of the script all along. A string macro
may appear e.g. in the expression defining the value of an arithmetic
variable, which is one way to convert string macro to an arithmetic
expression.

In the parallel version, the computational grid (and the geometry
array) are split between the thread: if a particular value belongs to
one subdomain it is not accessible to another subdomain (with the
exception of halo points, see below). On the contrary, any arithmetic
variable or string macro is available in all threads; however
depending on its use, they may have different values in different
threads.

Need to doublecheck the actual syntatic restrictions
on variable and string macro names and bring the code and this
manual in correspondence with each other.

## The Beatbox Scripting Language

After preprocessing (see below), a Beatbox script consists of a series
of *commands*. A command is a sequence of characters (being
part of one line of script or spanning across several lines),
beginning with a recognised keyword and ending with a semicolon
'`;`'. The following code excerpt shows examples of three commands:

```
def int sideLength 26; state xmax=sideLength, ymax=sideLength, zmax=sideLength, vmax=3;
euler
v1=[iext]
ode=lrd
par={ht=ht IV=@24}
;
```

This script defines `sideLength` which is the used initialise the
state of the system before the `euler` device is called.

The types of command in a Beatbox script are listed below. Each
command describes an action to be taken by Beatbox, with the keyword
at the beginning of the command being the verb.

- `rem` - A ‘remark’. Marks code as a comment. This is
  being processed by the interpreter as usual (so e.g. it should no
  contain undefined string macros), but otherwise makes no
  consequences for the simulation.
- `if` - Allows a script command to be conditionally
  read. The keyword is to be followed by an arithmetic expression
  followed by a command with its own keyword. If the arithmetic
  expression returns a nonzerp value, the command is interpreted,
  otherwise it is ignored. This does not affect the flow of execution
  as the simulation is run, but operates much like conditional
  compilation.
- `def` - Defines a variable or macro.
- `state` - Sets the dimensions of the computational grid.
- `screen` - Sets parameter for on-screen display for the
  run-time graphics.
- A device name - Adds an instance of the named device, with the
  parameters as described in the body of the command, to the ring of
  devices. Some of the recognized device names are discussed below; the
  full list is in `devlist.h`.
- `end` - Marks the end of the script. Anything after
  such statement is ignored.

## Preprocessing

As each line of the script is parsed, Beatbox preprocesses the code in
a manner similar to the C Preprocessor [see Kernighan and Ritchie,
1988, chap. 4]. The Beatbox preprocessor handles four tasks:

1. skipping comments,
2. including other Beatbox scripts,
3. calling system commands and
4. expanding string macros.

Each of these are discussed below.

### Comments

Comments can be added to Beatbox scripts in five ways:

1. A C style comment:

   ```
   /*
    Multi-line C style comment
   */
   ```
2. A C++ style comment:

   ```
   // Single-line C++ style comment
   ```
3. A `rem` statement:

   ```
   rem ’Remark’ command-style comment, 
       which can go over serveral lines but 
       must end with a semicolon;
   ```
4. Anything added after an `end` command,

   ```
   ...
   end;
   Anything here is now ignored by the Beatbox preprocessor so
   it could be used to comment code..
   ```
5. Anything within the body of any command which is not recognized as a
   valid `name=value` pair.

   ```
   Comment = As Beatbox does not know what the comment is this could be 
   treated as a comment (probably not good practice though);
   ```

Text in a comment is ignored by the parser, except undefined macros
will cause fatal errors within a body of a `rem` or another command.

### Including Other Beatbox Scripts

Where the relative path to a Beatbox script file is enclosed in in
angle brackets (< >), the content of the referenced file will be
read and inserted at that location. Unlike in the C programming
language, the name of the file in angle brackets is not preceded by an
`#include` command. This can be used to maintain
consistency across a number of simulations, or to reduce code
redundancy. Beatbox replaces a filename in angle brackets with the
file’s entire contents. For example, given a script
called `useful.bbs`:

Listing G.1: Script stored in a `useful.bbs` script file.

```
// Here is a lot of useful code...
def real apar=1;
def real bpar=2;
```

and a script that includes `useful.bbs` as follows:

Listing G.2: Another script that
includes `useful.bbs`.

```
// This is my own script
<useful.bbs>
// Here’s some more of my own code.
def real cpar=3;
```

The result, after preprocessing, is as shown below:

Listing G.3: Effective resultant script, after `useful.bbs`
has been included and comments eliminated.

```
def real apar=1;
def real bpar=2;
def real cpar=3;
```

The filename in the angular brackets may contain string macros which
are expanded in the usual way before the angular brackets operator is
applied.

### Calling System Commands

Beatbox replaces code in backticks (`` `...` ``) with the
result of that code when run as a system command, via
a `system()` call (`stdlib.h`). For example:

```
   `date '+%Y%m%d-%H:%M:%S'`
```

will be replaced with the result of the UNIX date command:

```
   20120710-09:30:55
```

### Expanding String Macros

String macros allow the user to define reusable strings that can be
pasted throughout a script. String macros are distinct from variables
in that they are expanded once, prior to the script being interpreted
and cannot therefore be assigned values other than when they are
defined. A string macro is defined as follows:

```
def str <macro name> <value>;
```

where `<macro name>` is a string using letters,
numbers, underscores (\_) or hyphens (-) and `<value>`
is any string not including a semicolon.

Need to doublecheck the actual syntatic restrictions
on variable and string macro names and bring the code and this
manual in correspondence with each other.

After a macro is defined, its name, wrapped in square brackets ( [ ] ) is
associated with its value. When Beatbox input script parser finds a
macro’s name in square brackets, it replaces them with the value.

In the following excerpt, the variable `hat` is assigned
the value `porkpie`. The variable `headware` is
assigned the value `hat`.

```
def str snack porkpie; // Assigned string ’porkpie’.
def str hat [snack];   // Assigned string ’porkpie’.
def str headware hat;  // Assigned string ’hat’, not value of hat macro.
```

A macro can expand to anything that could be typed in the script. For
example, a string macro can be used in place of
an `int`, `long` or `real`:

```
def str ninetynine 99;       // Assigned string value ’99’.
def int number [ninetynine]; // Assigned integer value 99.
```

A number variable cannot, however, be used to define a macro:

```
def int number 99;         // Assigned integer value 99.
def str ninetynine number; // Assigned string value ’number’.
```

It is possible to assign several lines of code (excluding semicolons)
to a string macro, so this:

```
def str instruction ppmout
when=out file="ppm/%04d.ppm" mode="w"
r=[u] r0=umin r1=umax
g=[v] g0=vmin g1=vmax
b=[i] b0=0 b1=255;
[instruction];
```

is equivalent to:

```
ppmout 
when=out file="ppm/%04d.ppm" mode="w"
r=[u] r0=umin r1=umax
g=[v] g0=vmin g1=vmax
b=[i] b0=0 b1=255;
```

## Defining Arithmetic Variables with the def Command

Arithmetic variables defined in the script are distinct from the C
language variables used in Beatbox’s implementation. For clarity,
variables defined in the script, using `def` may be
referred to as "k-variables".

A k-variable is defined using the `def` command, like this:

```
def <type> <name> [=] <value>;
```

where `<type>` is one of:

- `int` (integer number),
- `long` (integer number),
- `real` (real number),
- `float` (real number),
- `double`: (real number),

so k-variables and arithmetic expressions only have two data types,
real and integer, and their actual precision (`int`
or `long int`, `float` or `double`)
is determined at the compile time by the settings
in `k_.h`; typically
`long` and `double` respectively.

A `<variable name>` can consist of letters,
numbers, underscores (\_) or hyphens (-).

Variables defined in this way are accessible in the script
during the input script parsing, as well as at run-time by some
devices, e.g. `k_func`.

The `<value>` (optional) is an
expression that may be evaluated to the correct type and is assigned
as the initial value of the variable just defined. Expressions are
discussed in greater detail below. If no initial value is given, the
variable is assigned the initial value of 0 or 0.0 as appropriate.

Beatbox stores the names of macros wrapped in their square
brackets (`[...]`), therefore it is possible for a script to define
macros and variables with the same name.

## Predefined k-variables and string macros

Some variables and macros exist without being defined in the input
script. All of them are read-only, i.e. cannot be modified in the
input script. These are:

- `[0]` - string macro containing the name of the
  beatbox script, without the extension `.bbs`.
- `[1], [2], ...` - string macros containing the command line
  arguments passed to the script.
- `t` - integer, the counter of the device ring loops.
- `inf` - "infinity", a very big real number.
- `xmax,ymax,zmax,vmax` - integer, the computational
  grid sizes as defined by the `state` command, even if
  implicitly from the geometry.
- `Graph` - integer, 0 if `-nograph`
  command line option was given or the parallel version of the program
  is run, and 1 otherwise.
- `graphon` - integer, nonzero if graphical output has
  actually been initiated (e.g. connection to the X server successful).
- `XMAX,YMAX`  - integer, sizes of the graphical screen
  when run-time graphics is on, in pixels, as specified
  by `screen` command.
- `WINX,WINY` - integer, the coordinates of the
  graphical window on the screen, as specified by `screen`
  command.
- `BLACK,
  BLUE,
  GREEN,
  CYAN,
  RED,
  MAGENTA,
  BROWN,
  LIGHTGRAY,
  DARKGRAY,
  LIGHTBLUE,
  LIGHTGREEN,
  LIGHTCYAN,
  LIGHTRED,
  LIGHTMAGENTA,
  YELLOW,
  WHITE`  - integer codes of the standard VGA colours to be used in the
  run-time graphics.
- `pi` - the real number pi=3.1415926...
- `always` - the real 1.0.
- `never` - the real 0.0.

Apart from these, some devices have their own predefined variables
with the scope restricted to the body of that device.

## Expressions

Any numerical value in a Beatbox script, be it initial value in a
variable definition or the value of a device parameter, may be
specified as a mathematical expression. An expression may be a literal
value, such as 5.0, or the result of some computation, such
as `6*5` or `count/2`.

Beatbox expressions can use the standard infix arithmetic operators:
addition (+), subtraction (-), multiplication (\*), division (/) and
exponentiation (\*\* or ^), as well unary plus and minus. For more
complex operations Beatbox provides a collection of functions:

- `atan2(a, b)` - Returns the arctangent of `a/b`,
  using the signs of the arguments to compute the quadrant of the return
  value.
- `hypot(a, b)` - Returns the square root of the sum of the
  squares of `a` and `b`.
- `tanh(a)` - Returns the hyperbolic tangent
  of `a`.
- `erf(a)` - Returns the Gauss error function
  of `a`.
- `if(test, then, else)` - Returns `then`
  if `test` evaluates to true, `else`
  otherwise.
- `ifne0(test, then, else)` - Returns `then`
  if `test` is not equal to 0, `else`
  otherwise.
- `ifeq0(test, then, else)` - Returns `then`
  if `test` is equal to 0, `else` otherwise.
- `ifgt0(test, then, else)` - Returns `then`
  if `test` is strictly greater than 0, `else`
  otherwise.
- `ifge0(test, then, else)` - Returns `then`
  if `test` is greater than or equal to 0, `else`
  otherwise.
- `iflt0(test, then, else)` - Returns `then`
  if `test` is strictly less than 0, `else`
  otherwise.
- `ifle0(test, then, else)` - Returns `then`
  if `test` is less than or equal to 0, `else`
  otherwise.
- `ifsign(test, neg, zero, pos)` - Returns `neg`
  if `test` is strictly less than 0, `zero` if
  test is equal to 0, `pos` if `test` is strictly
  greater than 0.
- `gt(a, b)` - Returns 1.0 if `a` is strictly
  greater than `b`.
- `ge(a, b)` - Returns 1.0 if `a` is greater than
  or equal to `b`.
- `lt(a, b)` - Returns 1.0 if `a` is strictly less
  than `b`.
- `le(a, b)` - Returns 1.0 if `a` is less than or
  equal to `b`.
- `eq(a, b)` - Returns 1.0 if `a`
  and `b` are equal.
- `ne(a, b)` - Returns 1.0 if `a`
  and `b` are not equal.
- `mod(a, b)` - Returns the remainder of the integer division
  of `a` by `b`.
- `max(a, b)` - Returns the greater of `a`
  or `b`.
- `min(a, b)` - Returns the lesser of `a`
  or `b`.
- `crop(test, min, max)` - Returns `min`
  if `test` is strictly less
  than `min`. Returns `max` if `test`
  is strictly less than `max`. Returns
  `test` otherwise.
- `rnd(a, b)` - Returns a random real number greater than or
  equal to `a` and strictly less than `b`,
  uniformly distributed in between (sequential version only).
- `gauss(a, b)` - Returns a random real number normally
  distributed with mean `a` and standard
  deviation `b`, Box-Muller 1958 transformation (sequential version only).
- `u(x,y,z,v)` - Returns the value of dynamic
  variable `v` at point `(x,y,z)` in the mesh. If
  non-integer values are given for `x` or `y`, the
  value is linearly interpolated from surrounding `z`
  points. Non-integer values for `v` are rounded down (sequential version only).

The last three functions are not thread-safe or would be too
communicationally expensive to implement in the parallel mode, so are
only allowed in the sequential mode.

The arithmetic operators distinguish between real and integer numbers,
so `5/2` produces 2 while `5.0/2.0` gives
2.5. The usual type casting rules apply, e.g. sum of a real and an
integer is a real, etc. All functions take real arguments and return
real values. Calling a function with a wrong number of arguments is an
error. The built-in "k-compiler" of arithmetic expressions is not
optimizing, and any intellectual work is expected to be done by the
user. E.g. calling function with an integer argument,
say `0`, will generate the conversion of the
integer `0` into the real `0.0` in the compiled
code; where as putting `0.0` as the argument will not
require that so will generate a slightly shorter and more efficient
"k-executable".

## Briefly about Device-Defining Commands

The simplified syntax of a device-defining command is

```
<device> [<name>=<value> [...]] ;
```

The `<name>=<value>` pairs define the device
parameters and are separated from each other by spaces
(including `'\t'`, `'\b'`, `'\r'`
and `'\n'` characters). Each device has its own set of
parameters, whose names will be checked for in the body of the
command. The pairs
`<name>=<value>` with parameter names not
known to the given device, or something that is not
a `<name>=<value>` pair, are silently
ignored. If there is more than
one `<name>=<value>` pair with the same
name, only the first occurrence matters and the rest is/are
ignored. If no `<name>=<value>` pair
corresponding to a parameter is found, the
default value is assigned, or an error message is output and parsing
terminates if there is no default value for this parameter.

The parameters could be arithmetic, string, or blocks.

For the arithmetic parameters, which could be any valid arithmetic
C type, the `<value>` is taken as the string of
characters after the `=` sign until the first blank
character, is intepreted as an arithmetic expression, it is evaluated
to the appropriate k-type (integer or real), cast to the required C
type, and assigned to the parameter. A parameter in a device may have
a default value and maximal and minimal allowed values. If a
calculated value goes beyond the prescribed limits, and error
message is printed and parsing is terminated.

For the string parameters, the `value` (subject to
preprocessing) is intepreted as the literal value of the parameter. A
string containing blank spaces or one of the special characters
`\n`,
`\b`,
`\f`,
`\t`,
`\r`,
`\a`
and
`\0`
can be used as a value by enclosing it in double
quotes `"..."`. Any other character within the double
quotes, preceded by the backslash `\` (including the double
quote and the backslash itself) evaluates to that character.
String parameters may have default values, but obviosuly not minimal
or maximal values.

The block parameters expect the values in the form of text enclosed by
curly brackets, `{...}`. Such block text may span several
lines of the script and may contain contain its own set of
`<name>=<value>` pairs (what we shall call a
`codeblock`) or list of k-expressions
(`listblock`). There are no defaults for block parameters,
i.e. they are always compulsory.

## Defining the Computational Grid with the state Command

### General description

The first functional task of a script is to define the mesh. The
mesh is stored as a four-dimensional array, corresponding to a
three-dimensional, regular Cartesian mesh with an array of dynamic
variables at each point. Dynamic variables hold space-dependent
values, local to each point in the mesh. The majority of dynamic
variables are commonly employed to hold variables of the cell
model. The dimensions of the mesh and number of dynamic variables
(i.e. the size of the four-dimensional array) are set using
the `state` command.

The syntax of the `state` command is the same as that of a
device-defining command, only it does not add any devices into the
device ring.

The required set of parameters depends on whether geometry file is
used, which is determined by the string
parameter `geometry`. If this parameter is assigned to a
non-empty string, then it is understood to be the name of the
geometry file. This case is described in more detail below.

If parameter `geometry` is absent or is assigned to an
empty string, no geometry file is read, the computations are done in a
parallelepiped, and its sizes `xmax`, `ymax`
(compulsory) and `zmax` (optional, default=1) will be
checked for. Parameter `vmax` is still required.

The integer coordinates (index) `x` runs from 0 through to
`xmax-1` (so the name `xmax` is a bit of a
misnomer, but stuck for historical reasons).
If `zmax≥3`, the calculations will be three-dimensional
(3D). Otherwise, if `ymax≥3`, the calculations will be
two-dimensional (2D). Otherwise, if `xmax≥3`, the
calculations will be one-dimensional (1D). Otherwise, it is
zero-dimensional case (0D), that is, no spatial extent (ODE model). At
least three points in a particular direction are needed to make that
direction "extended", because in that case the marginal values of the
coordinates need to be kept free, for technical reasons related to
implementation of boundary conditions. The cases of
`xmax=2` etc are syntactically allowed but hardly ever used
in practice.

In any case, parameter `vmax` is accepted. It is
optional and defaults to 2 (a tribute to the FitzHugh-Nagumo
model). It defines the number of dynamic variables per node of the
computational grid, i.e. the number of computational layers. Note
that this number may and ofter is larger that the number of
equations in the model, as extra layers may be required as working
arrays for computational purposes (more detail below).

The mesh defined in the example below has 300
points along the x-axis and 400 points along the
y-axis. Since `zmax` is equal to 1, there is one point
along the z-axis, making the medium flat on the xy-plane. The
variable `vmax` specifies the number of dynamic variables
stored at every point in the mesh.

Listing G.4: Defining a two-dimensional mesh with
the `state` command.

```
state xmax=300 ymax=400 zmax=1 vmax=3;
```

Beatbox assumes that the simulation medium will follow a hierarchy of
axes; x before y before z, i.e. one-dimensional simulations must use
the x-axis and two-dimensional simulations must use the x and y axes.

Any Beatbox input script must contain exactly one `state`
command, and it should precede any device-defining commands.

### Anatomically Realistic Tissue Geometry

If you would like to use an anatomically realistic mesh for your
simulation, you can specify it in the geometry parameter of
the `state` module. For
example, `geometry=ffr.bbg` will select
the `ffr.bbg` geometry file.

When using a geometry file, there is no need to specify the dimensions
of the mesh, in fact Beatbox will complain if you do. You do still
need to define `vmax`, however, to suit your chosen RHS
module and any other dynamic variables your simulation needs. Beatbox
will also complain if the fibre directions specified in your geometry
file are not unit vectors. If you like, Beatbox will normalise the
fibre directions for you, if you set the `normaliseVectors`
parameter to 1. If you would like to model anisotropy, set the
`ansiotropy` parameter to 1. This only works when a
geometry is specified. When the `anisotropy=1`, the
behaviour and required parameters of some devices may change. In
particular, the `diff` device, performing the diffusion
substep, will require two diffusion coefficients, `Dpar`
and `Dtrans` for the diffusivity along and across the
fibres, instead of the isotropic `D`.

### Geometry File Format

The Beatbox geometry file will typically have the
extension `.bbg` and is a plain text file, each line of
which describes a point belonging to the tissue. Any such line will
have four numbers or seven numbers: integers for x, y and z
coordinates of a node and its tissue type, and three reals for the
fibre orientation data. The de-facto limits of the x-, y- and
z-components of all points in the file will be used to define the
circumscribed box. The file does not have to describe every point in
the box; the points that are not mentioned will simply be assumed to
belong to the void. A zero tissue type designates the void, so such
lines can be omitted without any loss of information. The three reals
of the fibre orientation data for any nonzero tissue type will be the
directional cosines of the fibres. If no true fibre data are available
in the source of the geometry, and/or no anisotropy is used in
simulations, then any triple can be put as the fibre orientation data,
say 1,0,0.

## More about Device-Definining Commands

Following the `state` declaration, a script can add to the
ring of devices by invoking or ‘calling’ Beatbox devices. A device
call takes the form of the device name, followed by parameters as
key-value pairs, separated by spaces. The excerpt below shows
the `euler` device being called with some parameters.

```
euler v1=[iext] ht=ht ode=lrd pars={IV=@24} name=Geoff;
```

Each device call will add an instance of the device to the ring of
devices. It is possible to instantiate the same device,
e.g. `euler`, multiple times and the parameters of each
instance will remain independent. The different instances can be
distinguished from each other (say, in the debugging or profiling
listings) by the values of their optional `name` parameter,
"Geoff" in the above example.

### Assigning Device Parameters

Device parameters are assigned following the device name as key-value
pairs with the syntax `<key>=<value>`. Since spaces separate
device parameters, an `str` parameter value that includes
spaces, should be enclosed in quotes (" "). Expressions assigned
to `int`, `long` and `real`
parameters will be reduced to literal values when read. An example is
shown in Listing G.5.

Some devices may request parameters formatted as a codestring or a
block.

- A codestring is provided in the same way as a string parameter, with
  the exception that the string must be a valid Beatbox script
  expression. The expression should compile to the variable type
  specified in the device’s documentation. Codestrings allow devices to
  evaluate an expression as the simulation is running, rather than
  reduce it to a literal value.
- A block is a string enclosed in braces ({ }). The contents of a
  block will be passed literally to the device, without intervention
  from the script interpreter. This allows a block to contain characters
  such as ';' or '"' that could not be included in a string parameter. Some
  devices, notably `k_func`, use a block to accept script
  statements. For instance, `euler` uses a block to pass
  parameters assignments to its RHS module.

Example uses of a codestring and a block are shown below.

Listing G.5 shows the assignment of a parameter,
`bar` to an imaginary `foo` device. Although the value of
`hat` may change throughout the simulation, the value of
the `bar` parameter will be set only once, when the device
is called. In this case, `bar` will be assigned the value
40.

Listing G.5: Assigning a device parameter.

```
def real hat 10.0;
foo bar = min(hat ,60)*4;
```

Listing G.6 illustrate usage of block parameters
in real devices `k_func` and
`euler`.
device.

Listing G.6: Assigning block parameters to the `k_func`
and `euler` devices.

```
k_func nowhere=1 pgm={
    stim = eq(t,stimTime);
};
def int neqn 24;
def str iext neqn;
euler v0=0 v1=neqn-1 ht=ht ode=crn par={ht=ht, IV=@[iext]};
```

In the `k_func`, the block parameter `pgm` is
assigned the string enclosed between the `{...}` brackets.
This will be compiled and result in a k-executable, which will be
stored among other internal parameters of this instance
of `k_func` device for future use. Then at each time step
(but only once per thread, as defined by the `nowhere=1`
parameter), this k-executable will be evaluated using the then
current, run-time values of k-variables `t`
and `stimTime`, and the result will be assigned to the
k-variable `stim`.

In `euler`, parameter `ode`, which is a string
defining the kinetic model used by the forward Euler timestepper, is
assigned `crn` which stands for the
Courtemanche-Ramirez-Nattel 1998 human atrial model. It uses layers
from 0 through to `neqn-1`=23 so
k-variable `neqn` must be equal to the number of dynamic
variables in the model (24), at the parsing time, otherwise program
will stop with an error message. Parameter `ht`, which
designates the time step of the forward Euler scheme, will be assigned
to the current (parse-time) value of the k-variable `ht`.
Parameter `par` will be assigned the whole contents within
the `{...}` brackets, and passed for further parse-time
processing by the ode-specific parser of the `euler`
device. On this occasion, the CRN model requires the time
step `ht` again, since it calculates new values of some of
the variables rather than calculating their time derivatives. Note
that using a different value of `ht` parameter
in `euler` device and in its `crn` kinetic model
would be syntactically correct, but would probably not do what you
want. This kinetic model also uses parameter `IV` which
corresponds to inter-cellular current. Its value is given
as `@[iext]`. In this example, string
macro `[iext]` expands to 24, the value of
`IV` will be different for every point, and it will be the
value taken from the layer 24 at that point (more about the layer
substitution below).

So the difference between the parse-time and run-time execution of
k-code within block parameters is not syntactical, but
device-specific. In other words, the only way to find out how it will
be interpreted is to look into the description of the particular device.

### Generic Device Parameters

A set of generic parameters, listed below, is applicable to all device
types. All of the generic parameters listed below are optional - if no
value is given for them, a default will be provided by
Beatbox. Generic parameters and their defaults are described below.

`(str) name`
:   Given name for the device. If the script specifies two instances of a
    device, giving one of the devices a name will disambiguate the devices
    in any output messages. Also, some devices refer to another device in
    the simulation using its name parameter. The default
    for `name` is the device name.

`(real) when`
:   The device's condition. The value given for `when` must be
    a real k-variable. The device will be run only on timesteps (loops of
    the device ring) when the value of this variable is nonzero. A
    literal value cannot be used for this parameter (think of this
    parameter as a codestring, where the code is restricted to just one
    real variable; allowing here a generic k-expression would be more
    consistent but also more expensive). For devices that should run on
    every timestep, Beatbox provides the predefined read-only
    variable `always`, whose value is 1,
    and `when=always` is the default.

Space Parameters
:   These specify the points of the mesh on which the device will operate:

    - `(int) nowhere`: if nonzero, the device is not
      associated with any part of the computational grid, and its
      execution does not involve any loops through `x,y,z`
      coordinates on the grid. The other space parameters have effect only
      if `nowhere=0`, which is the default.
    - `(int)x0` Lower `x` bound.
      Allowed values from 0 to `xmax-1`,
      defaults
      to `1`.
    - `(int) x1` Upper `x` bound.
      Allowed values from `x0` to `xmax-1`,
      defaults
      to `xmax-2`.
    - `(int) y0` Lower `y` bound.
      Allowed values from 0 to `ymax-1`,
      defaults to `0` for one-dimensional calculations and
      to `1` otherwise.
    - `(int) y1` Upper `y` bound.
      Allowed values from `y0` to `ymax-1`,
      defaults
      to `ymax-1` for one-dimensional calculations and to
      `ymax-2` otherwise.
    - `(int) z0` Lower `z` bound.
      Allowed values from to `zmax-1`,
      defaults to `1` for three-dimensional calculations and
      to `0` otherwise.
    - `(int) z1` Upper `z` bound.
      Allowed values from `z0` to `zmax-1`,
      defaults
      to `zmax-2` for three-dimensional calculations and
      `zmax-1` otherwise.
    - `(int) v0` Lower `v` (layer number)
      bound.
      Allowed values from 0 to `vmax-1`,
      defaults to `0`.
    - `(int) v1` Upper `v` (layer number)
      bound.
      Allowed values from 0 to `vmax-1`,
      defaults to `v0`.

    In general, a device will operate on points from, e.g. `x0 ≤ x
    ≤ x1`. In some devices, the `v0,v1` parameters do
    not define a range of `v` coordinate, but identify separate
    layers each having its own function. For example,
    Beatbox’s `diff*` devices use `v0` to indicate
    the dynamic variable containing transmembrane potential or another
    diffusing field, and `v1` to indicate where the Laplacian
    of that field should be stored. For devices that have no effect on the
    computational grid, the `nowhere` parameter should be set to
    1 to indicate this. The operation of some devices, in particular
    `k_func` is strongly affected by the `nowhere`
    parameter.

Window Parameters
:   These specify the placement and colouring of
    the area associated with the device within the graphical screen when
    run-time graphics is on.

    - `int row0` Lower row.
    - `int row1` Upper row.
    - `int col0` Left column.
    - `int col1` Right column.
    - `int color` Colour.

### The k\_func Device

All the standard devices (with the exception of ad-hoc, experimental
ones) are described in detail in
the Beatbox Device Reference
below. However the `k_func` device is so important for
understanding of the Beatbox control flow that it some consideration
immediately. This device takes a codeblock parameter
`pgm` which is expected to be a "k-program", that is a set
of one or more "k-assignments", separated by semicolons. A
k-assignment is a string of the form:

```
<k-variable>=<k-expression>
```

The expressions are compiled at parse-time, but evaluated and assigned
at run-time using the then current values of k-variables.

The functioning of the `k_func` device somewhat differs
depending on the value of its `nowhere` parameter.
If `nowhere` is nonzero, then the "k-program" is executed
exactly once per per time step, and it can only use the usual "global"
k-variables both in the right-hand sides and in the left-hand sides of
the assignments. If, however, `nowhere=0` and the device
has space, then at every time step the k-program is executed at every
point of the space. In this case, the k-programs may use the device's
"local" real variables:

- `x,y,z` are numerically equal to the corresponding
  integer coordinates of the point at which the k-program is executed;
- `u0, u1, ... u<vmax-1>`  - variables containing the
  corresponding values of the computational grid at the point at which
  the k-program is executed.
- `phasep, phaseu, p0, p1, ... p<vmax-1>`  - variables
  used for the phase-distribution method (see the next section).
- `np, nv` - if `file` parameter is set, these
  will be the the number of lines and the number of columns in the table
  read from the `file`.

All the local variables can be used in the left-hand sides as well as
the right-hand sides. The resulting values of `u0, u1,
...`  variables are stored back into the computational grid. The
resulting values of all other local variables will be lost after the
device instance finishes its work at the given time step. In this
mode, `k_func` can be used to fill the computational grid
with values according to given formulas or other algorithms.

A common and very important application of `k_func` device
in the `nowhere` mode is in updating variables used for
devices’ `when` parameters. For example, to run another
device at timestep 200 only, the `k_func` device
called below will assign the value 1.0 to `stim` when the
current timestep, `t` equals `stimTime`, and the
value of 0.0 otherwise:

```
def real stimTime 200;
k_func nowhere=1 pgm={
  stim = eq(t,stimTime);
};
```

The following device call adds a line to the `pgm`
parameter to set k-variable `print` to 1.0 on timesteps 0,
50, 100, ...
and to 0.0 otherwise:

```
def real stimTime 200;
def real printInterval 50;
k_func nowhere=1 pgm={
  stim = eq(t,stimTime);
  print = ifeq0(mod(t,printInterval));
};
```

The example
below illustrates use of `k_func` with a space
(`nowhere=0` mode). It models stimulation of the excitable
medium by raising the transmebrane voltage (allocated in the layer
whose number is encoded by string macro `[V]`), in the left
half of the box. The value of the stimulus linearly depends on the
`y` coordinate and varies linearly between 0 at
`y=0` and and 1.5 at `y=ymax-1`:

```
def str V 0;
k_func when=stim x0=1 x1=(xmax-1.0)/2 pgm={
  u[V] = u[V] + 1.5*y/(ymax-1);
};
```

### Phase Distribution

Device `k_func` implements a method that is very specific
for cardiac, and generically excitable media simulations. This is a
"phase distribution" method, particularly suitable for creating
initial conditions. The method was described e.g. in
V.N. Biktashev et al., Phil. Trans. Roy. Soc. London, ser A 347: 611-630, 1994.
The idea of the method is that the user a
scalar field, the phase, defined up to an integer multiple of 2π,
and Beatbox then automatically fills the space of the
`k_func` device with corresponding values of the dynamic
variables, where what values of dynamic variables correspond to what
values of the phase is defined by a "profile", provided in an input
file. For this purpose, `k_func` takes parameter
`file`, which will be the name of a text file, containing
space-separated columns of real numbers. Each column corresponds to
values of one dynamic variable, and the number of rows represents the
whole circle [0,2π) of the phase.

Figure 2: Conceptual arrangement of file for use with phase distribution.

For example, the following data file
contains a 2×10 table of values:

Listing G.7: Sample `k_func` profile file.

```
2.378  5.111
5.768  8.860
5.609  7.777
1.493  6.545
5.609  7.777
1.347  7.346
4.256  7.423
4.167  6.245
1.234  7.987
3.245  5.222
```

Conceptually, the values are arranged in a circle, as shown in
Figure 2.

---

Figure 3: `k_func`
interpolation. The value of `phaseu` is interpolated
between `ip` and `ip1` using `p`
and `q`.

If parameter `file` is given and correponds to a valid
profile, phase distribution is initiated by assigning a value to local
variable `phaseu` in the `k_func` program. The
value assigned to `phaseu`, taken to be in radians,
indicates a point on the circle, and consequently the line of the
input file to be read. Since `phaseu` is a real number,
linear interpolation is used to calculate values from two neighbouring
rows of array, as illustrated in Figure 3. The
calculated value is then assigned to the corresponding layer
of `u`: the values interpolated from the first column of
the profile to
`u0`, from the second to `u1` etc until
the last column available on the profile. Note that if the number of
`u*` is more than the number of layers allocated to this
`k_func` instance by its `vmin,vmax` parameters,
then the trailing values will be lost.

An alternative version of the phase distribution method is with
assignment to `phasep` local variable instead of
`phaseu`. In this case, instead of calculating the values
of `u0, u1, ...` local variables, the same algorithm is
used to calculate the values of local variables `p0, p1,
...`. Those local variables can then be used later in the same
k-program to calculate the `u0,u1,...` variables. Such
indirect assignment may be required if the columns in the profile go
in a wrong order etc.

## Putting it all together: a simple example

We now consider a simple working example of a Beatbox script, minimal.bbs. It corresponds to Figure 1 above, and solves a standard
initial/boundary-value problem for the FitzHugh-Nagumo model,
as defined in A.T.Winfree, Chaos
1:305,1991:

∂*u*/∂*t*
 = 
ε^-1^(*u*-*u*^3^/3-*v*)
 + 
*D*∇^2^*u*,
 
∂*v*/∂*t*
 = ε(*u*+β-γ*v*),
 
(*x*,*y*,*z*)  ∈ Ω
 = [0,*L_x_*]×
[0,*L_y_*] ;
  
∂*u*/∂**n** =  0,
 
(*x*,*y*,*z*)  ∈ ∂Ω ;
  
*u*(*x*,*y*,0)={-1.7,   *x*≤*L_x_*;   1.7,  
otherwise},
  
*v*(*x*,*y*,0)={-0.7,   *y*≤*L_y_*;   0.7,  
otherwise}.

It may be executed, say, in the sequential mode by using the
following command:

```
Beatbox_SEQ minimal.bbs
```

File `minimal.bbs`.

```
state xmax=102 ymax=102 vmax=3;

/* device control variables */
def real begin;
def real output;
def real end;	

/* Schedule */
k_func name=schedule nowhere=1 pgm={
  begin =eq(t,0);
  output=eq(mod(t,100),0);
  end   =ge(t,2000);
};

/* Initial conditions */
k_func name=ic when=begin
  x0=1 x1=100 y0=1 y1=100 pgm={
  u0=-1.7+3.4*gt(x,50);
  u1=-0.7+1.4*gt(y,50);
};

/* Diffusion substep */
diff v0=0 v1=2 D=1.0 hx=0.5;

/* Reaction substep */
euler v0=0 v1=1 ht=0.03
  ode=fhncub par={
  eps=0.2
  bet=0.8
  gam=0.5
  Iu=@2
};

/* Output image files */
ppmout when=output
  file=%04d.ppm
  r=0 r0=-1.7 r1=1.7
  g=1 g0=-0.7 g1=0.7
  b=2 b0=0.0  b1=1.0;

stop when=end;

end;
```

The expected results of the run are described below, and for now we consider the script
itself, which is shown on the right.

The script starts with a `space` command, which the 2D
computational grid of 100×100 internal nodes (`zmax`
defaults to 1), and three layers,
they will have numbers 0,1,2. Layers 0 and 1 will be used for
variables *u* and *v*, and layer 2 will keep the values of
the diffusion term *D*∇^2^*u*.

The "device control variables" `begin,output,end` are
required to define which devices work when. They are defined by the
three `def` commands, their values are updated by the
first `k_func` device and used as values of
the `when` parameters of other devices.

The first `k_func` device does not have a
`when` parameter, so its value defaults to
`always` and it is executed at each step. It has
`nowhere=1` so its k-program is not iterated over any grid
points, but only executed once. The k-program assigns value 1 to
k-variables `begin` at the very first loop of the
device ring, when the loop counter `t` is zero, otherwise
`begin` is assigned zero. Variable
`output` will be 1 at every 100th step, and 0
otherwise. And variable `end` will be zero until the step
2000, from which on it will be assigned 1.

The second `k_func` device calculates the initial
conditions in accordance with the formula above. It only works during
the very first loop (`when=begin`), and iterates over all
internal points, as specified by `x0,x1,y0,y1`. Its
k-program assigns the values to the `u0` local k-variable
(which means layer 0 value, i.e. *u* variable of the model)
and `u1` local variable (layer 1, *v* variable),
according to the formulas for the initial conditions above. The
k-expressions depend on local k-variables `x,y` each of
which runs from 1 through to 100, and the ranges 1..50 and 51..100
represent two halves of the computational box.

Notice that the two `k_func` devices use the optional
`name` parameter. This is used to tell them from each other
in the output.

The `diff` device calculates the diffusion term using
values in layer zero (`v0=0`) and puts the results into
layer two (`v1=2`). It uses the diffusion coefficient value
of 1.0, and `hx=0.5` is the spatial discretization
step. The device applies every step (no `when` parameter),
at all the inner points of the grid (no space parameters, so the
defaults are used).

The `euler` device makes the forward Euler timestep.
It also applies at every step and at all the inner points of the
grid. This device uses layers 0 and 1, as `v0=0`,
`v1=1`. It uses time discretization step
`ht=0.03`, and the right-hand sides of the FitzHugh-Nagumo
model are selected by `ode=fhncub`. The parameters of the
model are defined by the name-value pairs within the codeblock parameter
`pgm`, namely ε=0.2, β=0.8, γ=0.5. The
parameter `Iu` stands for the extra term in the right-hand
side for the *u* variable, which is defined here, through the
layer substitution call `@2`, as the value of
layer 2 at the same point, i.e. the value of the diffusion term as
computed by the previous `diff` device.

The `ppmout` device make the results of calculations
usable. Every time it is active, i.e. at every 100th time
step, it outputs a file in
the `ppm`
format. The space for this device is not specified so it defaults to
all inner points.

The discretization of the floating point data from the computational
grid to the one-byte unsigned integers in the PPM file is defined by
the following parameters. Integer `r=0` says that the
red-component will be made from the values in layer 0, i.e. values of
the *u* variable. Its values below `r0=-1.7` will be
mapped to "0" bytes (zero intensity of the red component), the values
above `r1=1.7` will be mapped to the maximal "255" bytes
(maximal intensity of the red component), with a linear interpolation
of values in between. Similarly, the (g)reen component is made out of
*v* values in layer 1, and the (b)lue component out of values of
the diffusion term in layer 2. Thus the head of the excitation wave is
red, its back is yellow and the refractory tail is green. The blue
component, represents the Laplacian but shows only the positive part
of it (as `b0=0.0`). This shows as a dark blue
stripe ahead of the front of the excitation wave, and a cyan stripe
around its back (the images can be seen
below).

The names of the output files are encoded by the string parameter
`file`.
The value of this parameter is treated as a file mask, where resulting
file names will have the form of the four
digits representing the file's ordinal number, followed by
`.ppm`, so the filenames will be
`0000.ppm`,
`0001.ppm`,
...,
`0020.ppm`.

The last device is `stop`, which does what it says on
the tin. Its only one parameter `when=end` means,
according to the assignment for k-variable `end` above,
that this device would work at every timestep, starting from 2000 and
above. But it only works once as after that Beatbox terminates.

The script is terminated by the `end` command, which is
purely syntactical, to signal that the script file is complete and the
rest of it, if any, should be ignored.

A sequential run of this script may produce standard output like this:

```
525 $ Beatbox_SEQ minimal.bbs
Beatbox v1.0
------------------------------------------------------------------------------------
Sequential version compiled Aug 31 2012 19:10:55 $
Execution begin at Tue Sep 25 18:24:11 2012 $
Input file minimal.bbs without additional arguments $
with options: noappend nodebug noverbose graph noprofile logname=minimal.log
state   /* grid 102 x 102 x 1 x 3 */ $
k_func $
k_func $
diff $
euler $
ppmout $
stop $
end $
Ring of 6 devices created: (0)schedule (1)ic (2)diff (3)euler (4)ppmout (5)stop $
STOP AT 2000[0]                                                                

BEATBOX_0.1 finished at t=2000 by device 5 "stop"
Tue Sep 25 18:24:14 2012
======================================================
526 $
```

and the file `minimal.log` with a bit more detail, such as
values of the device parameters read, and k-variables assigned. First the standard output and
the log file show the commands being read in; so if an error happens
at parse time, it is clear which device has caused it. Then the whole
device ring is described again, now giving devices' ordinal numbers in
the ring, and using
their proper names where given. After that there would be output from
devices produced during their work, or debug information if it was
specified, but in this example the only message is from the
`stop` device. The final message is a signal of normal
termination: stopping by any other device would probably happen due to
a fatal run-time error. Wall-clock times are printed before and after
the execution, so we see that this run took about 3 second.

If `ppm` format is not convenient, the output files can be
converted to another using e.g. an appropriate `netpbm`
utility, say (in `bash`):

```
 for (( i=0 ; $i<=20 ; i=$i+1 )); do n=`printf %04d $i`; pnmtopng $n.ppm > $n.png; done
```

Here are the resulting figures:

|  |  |  |  |  |  |  |
| --- | --- | --- | --- | --- | --- | --- |
|  |  |  |  |  |  |  |
|  |  |  |  |  |  |  |
|  |  |  |  |  |  |  |

The example we have considered is "minimal" and this sort of task is
routinely done by any cardiac simulation packages. However, Beatbox's
flexibility allows you to do much more than that. To discover
Beatbox's capabilities, do look at further sample scripts provided.

# Some more advanced scripts

Here we describe a few sample scripts that illustrate some typical
uses of BeatBox and can be used as templates for your specific
tasks. These scripts are located under:

- `data/scripts/sequential`
  for scripts illustrating the fundamentals, which can or should be run
  in sequential mode, and
- `data/scripts/parallel_Hector`
  for more specialized script illustrating parallel work.

These scripts are designed to introduce BeatBox devices in an
informal, "how-to" way; a more formal description of devices is given
later in the Beatbox Device Reference.


## Sequential

### fhn0.bbs

This script generates action potentials using single cell with
FitzHugh-Nagumo kinetics. See the script below:

- fhn0.bbs

The APs are initiated repeatedly: a stimulating pulse is issued every
time that the two dynamic variables satisfy certain inequalities,
meaning the system comes back close enough to the resting state. The
solution corresponding to the n-th action potential, `n=4`,
is output to the file (you need to run the code for this file to be
present):

- fhn0.rec

This file is used in the next example. This example illustrates the
use of:

- `k_func` as a feedback control device: stimulating
  shock is defined as a function of current cell state;
- `sample` device to convert grid value into a
  global k-variable, which is required for the `k_func`;- `screen` command and `k_draw` device for
    run-time graphics output: draw phase trajectory of the system as the
    solution progresses;
  - `record` output device to write contents of the grid
    (in this case, just single cell) to a file.
  - `clock` output device that shows integer simulation
    time counter.

Inputs to this script:

- none

Output of this script:

- The output of this script are run time graphics if using run-time
  graphics, and a record file called `fhn0.rec`.

To run this script with run-time graphics, call it using:

```
Beatbox_SEQ fhn0.bbs
```

and without graphics, use:

```
Beatbox_SEQ fhn0.bbs -nograph
```

The same applies to all examples with run-time graphics.

### fhn1.bbs

This script simulates propagation of excitation pulses in a
one-dimensional cable with FitzHugh-Nagumo kinetics. See:

- fhn1.bbs

The pulses are initiated by non-stationary non-homogeneous Dirichlet
conditions on the left boundary, where u-variable is made
time-dependent according to the record in the `fhn0.rec`
(the file obtained in the previous example). The input is included in
the fhn1.bbs script code and need not be given at command line. It
outputs a point record during n-th front, `n=5`, into
file: `fhn1.rec`. This record file will be used in the
next example. This example illustrates use of

- angle brackets `⟨...⟩` to include
  contents of another file;
- backticks `` `...` `` to capture output of a child process
  (here just counting the number of lines in the given file);
- `poincare` and `k_poincare` devices to
  detect arrivals of wavefronts at selected points, which is then used
  both to control execution and to calculate the propagation speed;
- `k_plot` run-time graphics device: to plot spatial
  profiles of the dynamic fields at selected moments of time.
- `pause` device for suspending execution and keeping
  the last picture on the screen until the user presses enter in the
  terminal window.

Inputs to this script:

- `fhn0.rec` obtained by running `fhn0.bbs`.

Output of this script:

- The output of this script are run time graphics if using run-time
  graphics, and a record file called `fhn1.rec`.

To run this script with run-time graphics, call it using:

```
Beatbox_SEQ fhn1.bbs
```

and without graphics,

```
Beatbox_SEQ fhn1.bbs -nograph
```

### fhn2.bbs

This script simulates a spiral wave in a two-dimensional square sheet with
FitzHugh-Nagumo kinetics, and periodically outputs the
solution. See:

- fhn2.bbs

Essentially, this is a variant of the `minimal.bbs` script
discussed above. Some new things that this script illustrates:

- more extensive use of k-variables to save typing and ease
  modification;
- use of `k_func` as a computational device for
  creating intial conditions by phase distribution method, using data
  from file `fhn1.rec` obtained by the previous fhn1.bbs;- use of computational devices `d_dt` and
    `grad2d` to compute time derivative and absolute value of
    spatial gradient of a field;
  - use of run-time graphics device `k_paint` to
    crudely visualize distribution of dynamic fields in the plane using
    16-colour VGA palette;
  - use of `singz` device which detects spiral wave tips
    and is both computational and output;
  - use of `shell` output device to call arbitrary OS
    command from BeatBox (here to create an output directory for the
    image files);
  - use of `imgout` and `k_imgout` output
    devices to output image files;
  - use `pause` device for suspending execution and keeping
    the last picture on the screen for a fixed time interval.- use of the `d_dt` device for computing numerical derivative
      of a chosen variable.

Note that this script will create file `fhn2.trj`
containing the records of the detected spiral tips, and directory
`fhn2.dir` which will contain several `png`
image files created by `imgout` and `k_imgout`
devices.

Inputs to this script are:

- `fhn1.rec` (file obtained by
  running `fhn1.bbs`)

Outputs from this script are:

- on-screen graphics if used with graphics;
- spiral wave trajectory in ASCII file `fhn2.trj`;
- png images of frames of the 2D simulation;

Before running this script, make sure that a correct fhn1.rec has been
created by running fhn1.bbs. To run this bbs script with graphics,
use the following command:

```
Beatbox_SEQ fhn2.bbs
```

and to run without graphics, use the following command:

```
Beatbox_SEQ fhn2.bbs -nograph
```

### b02.bbs

This script simulates propagation and destruction of a critical
solution in the model of in a-driven excitation front described in:

> V.N. Biktashev, "Dissipation of the excitation
> wavefronts", Phys. Rev. Lett., 89(16): 168102, 2002,

and reproduces figure 5 from that paper. See:

- fig5.bbs

This script reproduces both panels of the figure, and it is placed in
a separate subdirectory in which some post-processing is done as well.
The new things that this script illustrates are:

- Use of command-line arguments to vary a simulation parameter
  and the name of the output file;
- Slightly more complicated k-expressions for piecewise
  definition of the initial conditions;
- Use of `k_print` device to write the output files in
  the `png` format.

The directory
../data/scripts/sequential/b02/
also contains `Makefile` which describes the
workflow leading to creation of the final product,
`fig5a.png` and `fig5b.png` files. For this to
work,
netpbm has to be installed on your
computer.

The following steps can be done to reproduce the workflow:

- Create a fresh empty directory, say

  ```
  mkdir ~/b02-test
  ```
- Copy the prerequisite files into that directory. Assuming your
  copy of Beatbox is under `~/beatbox-1.2/`, this can be
  done by the following commands:

  ```
  cd ~/beatbox-1.2/data/scripts/sequential/b02/
  cp Makefile fig5.bbs ~/b02-test/
  cd ~/b02-test/
  ```
- The following command will show the entire workflow without
  actually doing it:

  ```
  make --dry all
  ```

  Using the output of this command as a guide, you can execute the
  whole workflow step by step, by typing or copy/pasting its output to
  your command line. Note that due to the `pause` device
  in the script, you would need to press enter in the terminal window
  used to launch BeatBox.
- The following command will actually do all the work automatically:

  ```
  make all
  ```

  If all works correctly, you will find files
  `fig5a.png` and `fig5b.png` in the
  `~/b02-test/` directory. Compare them with the ones
  obtained by the BeatBox developers, which are back in
  `~/beatbox-1.2/data/script/sequential/b02/` directory.

Command line inputs to this script are:

- value of sigma (0.624 or 0.625)- name of output directory

The outputs from this script are:

- onscreen graphics if used with graphics;- pgm output files for visualisation

Before running this script, please read sec 4.1.4 of beatbox.html.
This script comes with a `Makefile` that will allow you to
do the simulation. To use this `Makefile` for the
simulations, type at the command line:

```
make all
```

Alternatively, to run this bbs script from command line with graphics,
use one of the following commands::

```
Beatbox_SEQ fig5.bbs 0.624 output_dir   or
Beatbox_SEQ fig5.bbs 0.625 output_dir
```

and to run without graphics, use the following command:

```
Beatbox_SEQ fig5.bbs 0.624 output_dir -nograph or
Beatbox_SEQ fig5.bbs 0.625 output_dir -nograph
```


## Parallel

### scroll.bbs

This script simulates a scroll waves in a cuboidal 3D domain with
Barkley kinetics and explicit Euler approximation of diffusion. It is
a short crude model of actual simulations used in H. Dierckx et al.,
``Buckling of scroll waves'' Phys. Rev. Lett., 109(17):174102, 2012
(that paper also used an alternating-direction semi-implicit
approximation of diffusion, but it is still to be documented). See:

- scroll.bbs

The script uses phase-distribution method to create initial
conditions in the form of a slightly twisted scroll wave, using a
pre-recorded file `bkl1.rec` in the same directory.
Further things to note in this script are:

- The first command-line argument is the name of the pre-recorded
  file used for the initial conditions.
- The second command-line argument is a number used as part of the
  name of the output directory. It will be 0 for the sequential run
  and will be a number of threads for parallel runs.
- The `diff` device is called twice as in that model both
  equations contain diffusion terms, and with different diffusion
  coefficients. Correspondingly, two extra layers `[iu]`
  and `[iv]` are used to keep the values of these terms
  instead of just the usual one.
- The `ppmout` device is used to output a
  "three-dimensional image". It has the usual PPM header, showing the
  x- and y-sizes of the 3D array it contains, as width and height
  respectively. However this header is followed not by a
  `nx×ny`
  array of byte triplets, but by `nz` such
  arrays, thus making the whole 3D `nx×ny×nz`
  array. Note that the "3D ppm" format used for that is NOT the
  standard multi-image ppm, which also can have `nz` arrays
  going one after the other, but there each of them has its own
  header. Our "3D ppm" format is not standard but it is understood by
  the `EZView` viewer (see below).
- The `singz` device outputs not the position of the tip of
  the spiral wave, as in 2D simulations discussed above, but a
  position of such a tip in every `z=const`
  cross-section. Since in this simulation the filament of the scroll
  wave goes monotonically in vertical direction, such collection of
  spiral tips effectively makes up a description of the filament of
  the scroll wave. This output was used for processing to measure
  "buckling" of a scroll wave in the above cited paper. The
  visualization of the scroll filament can be done independently by
  the visualizer, see below.

The directory
../data/scripts/parallel/Barkley/
also contains `Makefile` which describes the
workflow leading to creation of the final product,
`bkl1.rec` pre-recorded file used for the initial
conditions, and `report.dat` which contains the table of
run time in seconds vs number of processors used when this script was
tested by the developers on a MacPro workstation. As in
`b02.bbs` example above, you can use `make --dry
all` to see the workflow involved, or just `make all`
to do it all automatically.

The `report.dat` obtained by the developers for BeatBox
version 1.2.484 on a MacPro with 2 x 2.4 GHz 6-Core Intel Xeon
processor contained

```
1	 207.352903 
2	 105.242710 
4	 55.027358 
8	 31.047070 
16	 30.074329
```

which indicates that parallelization is effective only up to about 8
processes and further increase of number of processes does not offer
any speed-up. Presumably this is due to the relatively small size of
the box, so communication cost start dominating
after `np=8`.

**Exercise:** Increase the size of the box twice in each
direction and repeat the scaling test.

**Answer:** On the same computer as above, the
resulting `report.dat` contained:

TBD

Visualization of the resulting "3D ppm" files can be done using
`EZView`. To this end, the
../data/scripts/parallel/Barkley/
directory contains Perl script `view.pl`. As can be seen
from the `Makefile`, to view the result of simultion on 8
processors, you say:

```
make view-8
```

on the command line, etc. The Perl script creates an appropriate "task
file" in the relevant directory, and then calls `ezscroll`
binary which thus has to be already installed on your computer and
visible in your `$PATH`. See
http://empslocal.ex.ac.uk/people/staff/vnb262/software/EZView/
 for the EZView source code and instruction; this particular
scroll will work with version 1.1 (beware, different versions of
EZView use different format of task files).

Command line inputs:

- `bkl1.rec` (provided with this bbs script)
- number of MPI processors (integer value)

The outputs from this bbs script are:

- dump file called scroll.dmp (in out\_dir)
- sampled variables in history.dat (in out\_dir)
- 3D ppm files (in directory called ppm)
- scroll filament called singz.dat (in out\_dir)

Before running this script, make sure that the `bkl1.rec`
is in your working directory. You will also need the
provided `view.pl` (a Perl script) in your working
directory. To run this script in parallel and do the exercise, you
can edit the `scroll.bbs` file and then type:

```
make all
```

to do all the tasks in the simulation. To do the tasks individually,
see the `Makefile`.


# bbg (geometry) data and cell models

## bbg (geometry)

There is an extensive repository (and ever growing) of cardiac
anatomical models in Beatbox. These models are provided as ASCII text
files with an extension `.bbg` (e.g. `ffr.bbg`).
The way to include any of the following geometries into a simulation
is by means of a state device call in the bbs program as follows:

```
state file=name_of_your_geometry.bbg normaliseVectors=1 anisotropy=1 vmax=neqn+1;
```

The bbs program can then work out the bounding box xmax, ymax, and
zmax. If you have isotropic simulation, the normaliseVectors and
anisotropy parameters should not be included in the state call. A
brief description of the geometries, and asssociated bbs programs is
below. The space step is usually obtained from the authors of the
geometry, as is the species type (human, rabbit,...).

### ffr.bbg

This is a rabbit ventricle geometry. The space step in this geometry
is 0.1 mm. There is 1 tissue type in this geomtry. The bbs program
that can initiate a spiral wave and gives all other optimised
simulation paramters using LRD kinetics is:

- LRD\_ffr.bbs

The purpose of this script is to allow 3D simulation using the ffr.bbg
rabbit geometry. This bbs script also demonstrated the use of the
anisotropic diff device.

The inputs to this script are:

- `ffr.bbg` (this file should be in your working directory)

The outputs of this script are:

- 3D ppm files (in `sub-dir ppm/`)
- a record of all dynamical variables at given point location where
  the file is called `LRD_ffr.rec` in `out/`
- a dump file called `LRD_ffr.dmp`
  in `out/`

To run this simulation, make sure you have `ppm/`
and `out/` sub-directories. To run this simulation in
serial, use the command:

```
Beatbox_SEQ LRD_ffr.bbs -verbose
```

To run this in parallel using np number of processors, use the command:

```
mpirun -n np Beatbox LRD_ffr.bbs -verbose -profile
```

### ffr\_slice.bbg

This is a slice of the above rabbit ventricle geometry. The space step
in this geometry is 0.1 mm. There is 1 tissue type in this
geometry. The bbs program that can initiate a spiral wave and gives
all other optimised simulation paramters using LRD kineitcs is:

- LRD\_ffr\_slice.bbs

The purpose of this script is to allow 2D simulation using an uneven
geomtry. The inputs to this file are the geometry,
i.e. ffr\_slice.bbg. The 2D geometry has fibre orientation and this
file demonstrates the use of the anisotropic diff device. The output
from this script are 2D ppm files (in the sub-dir ppm/). To run this
simulation in serial, use the command:

```
Beatbox_SEQ LRD_ffr_slice.bbs -verbose
```

To run this in parallel using np number of processors, use the command:

```
mpirun -n np Beatbox LRD_ffr_slice.bbs -verbose -profile
```

## Cell models

There is an extensive repository (and every growing) of
electrophysiological and electro-mechanical cardiac cell models in
Beatbox. These cell models are provided as modules and are assigned a
name that can be used in the bbs programs. The cell model module is
accessed in a call to the ode solver, i.e. euler device. The euler
device's generic syntax is:

```
euler v0=0 v1=neqn-1 ht=ht ode=my_cell_model_name rest=100 par={
ht=ht;
Iu=@[iext];
par1=val1;
par2=val2;
};
```

where `neqn` is the number of ODEs in the cell model, and
par1 and par2 are cell model specific parameters that you may want to
modify (e.g. increase gCaL). Assuming that your diffusing variable is
voltage, the layer of voltage is explained in the sub-sections
below. See Beatbox Cell Model
Reference below for several cell model available in the package.

# Beatbox Developer's Guide

## Hello Beatbox!

Devices are the primary building block of a Beatbox simulation. A
simulation consists of two or more devices that are called in turn, at
most once for each simulation timestep. When called, the device can
perform a task, with optional access to the simulation
medium, `New`.

In this tutorial, we’ll build a device to output the text
“`Hello, Beatbox !`” in the simulation’s standard output
file. We won’t go into much detail in this section, rather we’ll just
look at the absolute minimum one has to do to build a working device.

### The Device Skeleton

The template below shows the a `.c` file containing the
basic outline of a minimal device.

```
#include <assert.h>
#include <stdio.h>
#include <stdlib.h>
#include <string.h>

#include "system.h"
#include "beatbox.h"
#include "device.h"
#include "state.h"
#include "bikt.h"
#include "qpp.h"

typedef struct {
int dummy;  // Add your own device -specific storage here.
} STR;

RUN_HEAD(myDevice)
/* Put code to perform the device ’s task here. */
RUN_TAIL(myDevice)

DESTROY_HEAD(myDevice)
/* Free any allocated resources here. */
DESTROY_TAIL(myDevice)

CREATE_HEAD(myDevice)
/* Add any initialisation code here. */
CREATE_TAIL(myDevice ,1)
```

There are four key parts to a device:

1. The first is the definition of the `STR` datatype. Every
   device in Beatbox defines `STR` to meet its specific
   storage requirements. Usually, `STR` is used to define a
   structure for any constants required by the device. For our
   “`Hello, Beatbox !`” device, we won’t need any persistent
   storage, so we’ll just use the minimal implementation that’s given in
   the template. We’ll see how to use `STR` in more detail in the
   Anatomy of a Device section.
2. The second part of interest is the function used to run our
   device. Code between the `RUN_HEAD`
   and `RUN_TAIL` macros defines what the device does. This is
   where whatever computation or output tasks to be performed by the
   device will happen. For our “`Hello, Beatbox!`” device,
   this is where we want to output our message. We’ll use
   the `MESSAGE` macro, defined in `beatbox.h`,
   which will print to both
   `stdout` and the simulation’s `.res` file.

   ```
   RUN_HEAD(hello)
   MESSAGE("Hello , Beatbox!\n");
   RUN_TAIL(hello)
   ```

   We’ll also have to change the name in the parentheses of
   the `RUN_HEAD` and `RUN_TAIL` macros
   from `myDevice` to `hello`. `hello`
   will be the name of our device, and we’ll need to use it wherever we
   refer to the device. Device names cannot use spaces and are, by
   convention, all lower-case, without punctuation. If you really need to
   separate words, use an underscore (\_).
3. The function defined by expanding the `DESTROY_HEAD`
   and `DESTROY_TAIL` macros is called only once, at the end
   of a simulation, and should be used to free any allocated
   resources. Since our device won’t allocate anything, we don’t have
   anything to free, so we can leave this function empty and just put the
   device name in the parentheses:

   ```
   DESTROY_HEAD(hello)
   /* Nothing to do here. */
   DESTROY_TAIL(hello)
   ```
4. The fourth and final part prepares the device to be run. The function
   defined by expanding the `CREATE_HEAD`
   and `CREATE_TAIL` macros will be called only once, at the
   start of the simulation and should be used to perform any
   initialisation tasks required by the device. This is discussed in much
   more detail in the Anatomy of a
   Device section. For our “`Hello, Beatbox!`” device, we
   needn’t add any code here, so just put the device name in the
   parentheses.

   ```
   CREATE_HEAD(hello)
   /* Nothing to do here. Quite boring , actually. */
   CREATE_TAIL(hello ,1)
   ```

   Although we’ve not put any code between the `HEAD`
   and `TAIL` macros, it’s important not to remove them
   altogether, as they are all referred to elsewhere in Beatbox. Also, be
   sure not to change the order of the macros,
   since `CREATE_TAIL` defines code dependent upon
   the `RUN_HEAD` and `DESTROY_HEAD` macros. We
   can now save our device file. By convention, the
   device’s `.c` file should have the same name as the device,
   so ours will be `hello.c`.

### Registering our Device

Now that we have a new device, we need to inform Beatbox that it’s
available for use in a simulation. We do this by listing our device’s
name in `devlist.h`. The name listed here must match
exactly (case matters) the name we used in our device code.

Open `devlist.h` and add the following line:

```
D(hello)
```

It’s good practice to add your device in alphabetical order. You’ll
see a few devices in there that are listed
as `S(somedevice)`. These devices will only work in
sequential mode, and will be disabled in parallel. They’re naughty
devices. Since we don’t want our new device to fall in with this rough
crowd, we’re going to use `D()`, ok?

### Building Beatbox with our Device

Beatbox is built using the GNU Autotools. To include our new device in
the build, we must add it to `Makefile.am`, which, like
your `hello.c` file, should be in the `src`
directory. `Makefile.am` contains a simple description of
what’s required to build the software. When we’re done changing it,
we’ll use `automake` to generate a
new `Makefile`, from which the code will actually be built.
Open `Makefile.am`. Scroll down the file, past the flags
and other stuff until you find the line:

```
common_sources = \
```

followed by a long list of `.c` and `.h`
files. In correct alphabetical order, add the following line to the
list:

```
hello.c\
```

We can now build Beatbox with our new device. First, we need to
generate a new `Makefile` by invoking `automake`
from the `beatbox` directory.

```
$ pwd
$ /Users/rossmcf/Working/beatbox
$ automake
```

With `automake`, no news is good news. We can now compile
our new version of Beatbox containing the `hello`
device. We’ll run the local `configure` script to set up
the build system for our machine, and then make to actually build the
code.

```
$ pwd
$ /Users/rossmcf/Working/beatbox
$ ./configure
... (Lots of stuff)
$ make
... (Lots more stuff , hopefully no errors.)
```

This will compile the code. Assuming all is well, you can now install
the binary wherever you want it.

```
$ make install
... (Even more stuff.)
```

Don’t be too worried about all the text whizzing by. If anything goes
amiss, the last line usually tells you all you need to know.

### Running our Device

To include our new device in a simulation, we use the device’s name in
the script. So, to print our “`Hello, Beatbox!`” at each
timestep, we include the following line, making sure to leave a space
between the device name and the semicolon: `hello ;` You
can try using the following simple script, let’s call
it `basic.bbs`, to test it:

```
// The medium
state xmax=1 ymax=1 zmax=1 vmax=4;

// Schedule and display parameters
def real begin;
def real end;
k_func nowhere=1 pgm={
begin =eq(t,0);
end =ge(t,20);
};

// Our new device!
hello ;

stop when=end;
end;
```

Running the script with the following command:

```
$ <mpirun commands> Beatbox basic.bbs <-options>
```

should produce something like:

```
Beatbox 
------------------------------------------------------------------------------------
BEATBOX_1.0 -- MPI version date Oct 26 2010 22:11:47 $
execution begin at Tue Oct 26 22:14:12 2010 $
Input file basic.bbs without additional arguments $
with options: noappend nodebug noverbose nograph noprofile resname=basic.res
state  Simulation medium (1 x 1 x 1)
Domain decomposed as (001,001,001).
0 processors of 1 remain idle.
$
k_func $
hello $
stop $
end of input file $
Loop of 3 devices created: (0)k_func (1)hello (2)stop $
Hello , Beatbox!
Hello , Beatbox!
Hello , Beatbox!
Hello , Beatbox!
Hello , Beatbox!
Hello , Beatbox!
Hello , Beatbox!
Hello , Beatbox!
Hello , Beatbox!
Hello , Beatbox!
Hello , Beatbox!
Hello , Beatbox!
Hello , Beatbox!
Hello , Beatbox!
Hello , Beatbox!
Hello , Beatbox!
Hello , Beatbox!
Hello , Beatbox!
Hello , Beatbox!
Hello , Beatbox!
Hello , Beatbox!
TIMING INFO
-------------------------
This run took 0.000266 seconds.
BEATBOX_0.1 finished at t=20 by device 2 "stop"
```

So, now that you’ve built your first device, you might want to make
one that actually does something. For that, you’ll need to read
Anatomy of a Device sectin.

## Anatomy of a Device

This section assumes that you’ve read Hello
Beatbox! section and have built a Beatbox device using the method
described therein. We’re now going to go a little more slowly, taking
the time to see how and why things work they way they do in
Beatbox. Much of the generic code required of a device is provided as
a collection of macros and type definitions in `device.h`,
which must be included by all devices.

### The Parameter Structure

For the persistent storage of data, devices must declare a parameter
structure using the `STR` datatype. `STR` is a
marker type that should be defined, using `typedef` , as
a `struct`. Typically, the `STR` struct has
fields for one or more user-specified parameters and any other
constants required by an instance of the device. A minimal
implementation, as shown in `hello.c`, above, should
define `STR` as a struct with at least one field.
Commonly, the parameter structure is used to hold values of parameters
read from the script. This process is described in
the Reading Parameters subsection.

### The Device Datatype

The `Device` datatype (`device.h`) represents an
instance of a device, as called in the user script.
One `Device` structure is allocated for each of the device
calls in the script, such that if the same type of device
(e.g. `k_func`) is called multiple times, there will be
multiple `Device` structures. For the most part,
the `Device structure` is only used ‘behind the
scenes’. One field of the `Device` structure that you will
see a lot, however, is the `Space` structure.

Your device’s space determines the points and dynamic variables of the
simulation medium on which it should operate. A device’s
implementation code should not interact directly with
the `Device` structure, so we won’t look at it any
longer. Forget you even saw it. Some minimal interaction with the
`Device` structure is provided in the function template
macros defined in `device.h`. Using these is the best way
to get the job done without wandering into any dangerous territory.

### Device Function Templates

As we saw in the Hello Beatbox! section,
your device needs to define three functions, referred to
as `Create`, `Run`
and `Destroy`. `device.h` defines three template
macros that will wrap around your function implementations. Using
these templates is strongly recommended since they ensure reliable
operation of devices, keep code concise and reduce redundancy. Each
template takes the form of a `HEAD` and `TAIL`
macro. These open and close the function respectively, and throw in
some standard functionality for good measure.

The head macros take at least one argument, the name of the
device. This is included into the function’s name when the macro is
expanded, so that, for example, the `euler` device will
have functions
called `create_euler()`, `run_euler()` and
`destroy_euler()`. Your chosen device name must be unique
and used consistently throughout all macros and
in `devlist.h`.

### CREATE\_HEAD and CREATE\_TAIL

You device’s `Create` function performs any initialisation
required for the device. The function is run once only, before the
simulation begins. The `CREATE_HEAD` macro declares and
allocates memory for the device’s parameter structure, `S`,
a local variable of type `STR`. You can use the body of the
function to assign values to the parameter structure.

The defined function will have the name
of `create_<name>`, where
`<name>` is the argument to
the`CREATE_HEAD` macro. To avoid name collisions, it is
strongly recommended that `<name>` correspond to the
name of the device.

The `create_<name>` function has two
parameters, `dev` and `w`. `dev` is a
pointer to the `Device` structure and is used by the
`CREATE_TAIL` macro to assign values to its
fields. `w` is a pointer to the parameter string taken from
the device call in the user script. This can be used in combination
with the functions defined in `qpp.h` to acquire values
from the user script. For more details on how to do this, see
the Reading Parameters subsection.

The `CREATE_TAIL` macro assigns the address
of `S` and pointers to functions
named `run_<name>` and
`destroy_<name>` to
the `par`, `p` and `d` fields of the
`Device` structure respectively.

Although the `Create` function has access to
the `Device` structure at this point, it is perilous to
tamper with the device structure, so just don’t.

### RUN\_HEAD and RUN\_TAIL

Your device’s `Run` function describes the task it will
perform. The function will be called `run_<name>`
where `<name>` is the argument to the `RUN_HEAD
macro`. The `Run` function is pointed to by the `p`
field of the `Device` structure, with
the `s`, `w` and `par` fields of the
same structure passed as arguments.

- `s` is the device’s `Space` structure, which is
  discussed in the Accessing New
  subsection.
- `w` is the device’s `Window`
  structure.
- `par` is the device’s parameter structure.

The `RUN_HEAD` macro contains code that
assigns `par` to a local variable, `S`, of type
`STR`. Fields of the parameter structure can then be
accessed using `S->fieldName`. Commonly,
the `Run` function begins by retrieving values from the
parameter structure to local variables. This can be done using
shortcut macros defined in `device.h`. We discuss this in
more detail in the Reading
Parameters subsection.

The `RUN_TAIL` macro closes the function, returning 1 to
indicate success. If the `Run` function of any device
returns, 0 Beatbox will stop the simulation immediately.

### DESTROY\_HEAD and DESTROY\_TAIL

In your device’s `Destroy` function, it should put its toys
away; it’s time for bed. Specifically, the `Destroy`
function should free any memory allocated for the device and close any
open files. If you use the provided template macros, there is no need
to free `par`/`S`. The function is pointed to by
the `d` field of the `Device` structure, with
the `s`, `w` and `par` fields of the
same structure passed as arguments.

- `s` is the device’s `Space` structure.
- `w` is the device’s `Window` structure.
- `par` is the device’s parameter structure.

The `DESTROY_HEAD` macro will again point local
variable `S` to your device’s parameter
structure. The `DESTROY_TAIL` macro frees `par`
and returns 1 to indicate success.

### Accessing New

Beatbox’s simulation medium is a four-dimensional data set - multiple
dynamic variables or layers at points in three-dimensional space.
Beatbox represents the simulation medium with a one-dimensional array
called `New`, defined in `state.h`. In addition
to exporting `New`, `state.h` provides a
collection of variables and macros with which to navigate it. These
are listed in Table I.1.

Table I.1: Public `state` Variables.

| Name | Description |
| --- | --- |
| `t` | The current time step. |
| `New` | The simulation medium. |
| `vmax` | Number of layers. |
| `xmax` | Number of points in the x-axis. |
| `ymax` | Number of points in the y-axis. |
| `zmax` | Number of points in the z-axis. |
| `vmax_xmax` | Number of variables in the v/x plane. |
| `vmax_xmax_ymax` | Number of variables in the v/x/y plane. |
| `DX` | The number of array locations between neighbouring points on the x-axis. (See Figure 2.8) |
| `DY` | The number of array locations between neighbouring points on the y-axis. (See Figure 2.8) |
| `DZ` | The number of array locations between neighbouring points on the z-axis. |
| `DV` | The number of array locations between neighbouring points on the v (variable) axis. |
| `dim` | The dimensionality of the mesh, from 0 (single cell) to 3. |
| `ONE` | 1 if the dimensionality is greater than or equal to 1. 0 otherwise. |
| `TWO` | 1 if the dimensionality is greater than or equal to 2. 0 otherwise. |
| `TRI` | 1 if the dimensionality is greater than or equal to 3. 0 otherwise. |

To access a point in `New`, use the `ind()`
function to obtain the index of the desired
location. `ind()` takes 4
coordinates, `{x,y,z,v}`. For a pointer, you will commonly
use `New+ind(x,y,z,v)`, and for an absolute
value `New[ind(x,y,z,v)]`.

When accessing `New`, your device MUST only alter values at
points within its space, i.e. `i0 <= i <= i1`. When
iterating over the medium, it is advised that the space structure’s
fields be used as loop bounds. Not doing so will produce inconsistent
behaviour for users.

```
RUN_HEAD(example)
int x, y, z;
real HUGE *u;
for(z=s.z0;z<=s.z1;z++){
    for(y=s.y0;y<=s.y1;y++){
        for(x=s.x0;x<=s.x1;x++){

            u=New+ind(x,y,z,s.v0);
            /* do something to u here */

        }
     }
}
RUN_TAIL(example)
```

Accessing points immediately `(x ± 1,y ± 1,z ± 1)` outside
of the device’s space is acceptable, provided such access is
read-only. If your device does reference neighbouring points in this
fashion, it MUST include the `DEVICE_REQUIRES_SYNC` macro
in its `Create` function. This will ensure that the device
continues to operate correctly in parallel.

Great care should also be exercised when writing to locations
in `New`. Since `New` may hold values relating
to more than one timestep, the mathematical procedure may be corrupted
if values required by other devices are overwritten. In general,
operations on `New` MUST be commutative — the device MUST
NOT assume a particular order of execution. In addition, devices that
are not explicitly parallelised MUST NOT assign values derived from
`New` to their parameter structure, or any other global
variable. This will stop terrible things from happening when the
device is run in parallel.

To keep users happy, layers accessed by your device should be
specified via the `Space` structure. In most
cases, `v0` and `v1` describe a range, but some
devices use them separately, e.g. `s.v0` as ‘source’
and `s.v1` as ‘destination’.

### Tissue Geometry

If your device is interested in accessing data about the underlying
tissue geometry, you’ll find it
in `Geom`. `Geom` is organised in much the same
way as `New`, and we use `geom_ind(x,y,z,v)` to
access it. The `v` variable can have one of four values:

- `GEOM_STATUS`,
- `GEOM_FIBRE_1`,
- `GEOM_FIBRE_2`,
- `GEOM_FIBRE_3`.

To test if a point is tissue, use `isTissue(x,y,z)`. The
same rules apply to `Geom` as to `New`, in
particular, stick to your `Space` structure and don’t
reference any further away than `( x ± 1,y ± 1,z ± 1)`.

### Reading Parameters

Commonly, a device will take some parameters from the user via the
script. We call this process ‘accepting’ a parameter. There are two
steps to this process:

1. Firstly, the parameter is read by the script
   preprocessor, and assigned to the device’s parameter structure.
2. Later, in order to use the parameter, the device will retrieve the
   value from the parameter structure. Both of these steps are greatly
   simplified by some handy macros defined in `qpp.h`.

### Parameter Accept Macros

Parameter accept macros provide a quick way to pull a parameter value
from the script and assign it to its namesake field in the device’s
parameter structure. The first thing to consider is the type of
parameter you’d like to accept. There are four common types:

- `Integers` Requires an `int` in the
  parameter structure.
- `Real Numbers` Requires a `double` in the
  parameter structure.
- `Strings` Requires a `char[]` array in the
  parameter structure.
- `Files` Requires two fields in the parameter structure;
  a `FILE *` with the same name as the parameter, and
  a `char[]` array of size `MAXPATH` with the name
  `<param>name`, where `<param>` is
  the name of the parameter.

The corresponding parameter accept macros are listed below. They each
have a default argument that, if given the corresponding null value,
will require that the user supplies a value, rather than merely making
it optional.

``` ACCEPTI(name,deflt,minv,maxv); ```
:   - `name` Name of the parameter whose value is to be read.
    - `deflt` Default value, in case no parameter
      called `name` is found. If `INONE`, a script
      parameter MUST be found.
    - `minv` Minimum allowed value. If `INONE`, no
      minimum is prescribed.
    - `maxv` Maximum allowed value. If `INONE`, no
      maximum is prescribed.

``` ACCEPTR(name,deflt,minv,maxv); ```
:   - `name` Name of the parameter whose value is to be read.
    - `deflt` Default value, in case no parameter called
      `name` is found. If `RNONE`, a script parameter
      MUST be found.
    - `minv` Minimum allowed value. If `RNONE`, no
      minimum is prescribed.
    - `maxv` Maximum allowed value. If `RNONE`, no
      maximum is prescribed.

``` ACCEPTS(name,deflt); ```
:   - `name` Name of the parameter whose value is to be
      read.
    - `deflt` Default value, in case no parameter called
      `name` is found. If `NULL`, a script parameter
      MUST be found.

``` ACCEPTF(name,mode,deflt); ```
:   - `name` Name of the parameter whose value is to be
      read.
    - `mode` Access mode in which the file is to be
      opened. Syntax is identical to a `fopen()` call:
      - `r` or `rb` Open existing le for reading.
      - `w` or `wb` Create le or wipe existing le
        before writing.
      - `a` or `ab` Append to end of existing le,
        creating if necessary.
      - `rt` or `rbt` or `rtb` Open
        existing le for updatereading and writing.
      - `wt` or `wbt` or `wtb` Create le
        or wipe existing le before updating.
      - `at` or `abt` or `atb` AppendOpen
        or create le for update, writing at end of le.
    - `deflt` Default filename, in case no parameter called
      `name` is found. If `NULL`, a filename MUST be
      found in the script.

### Parameter Shortcut Macros

To aid access to fields of the parameter
structure, `device.h` provides shortcut macros to declare
local variables and initialise them with values from their namesake
fields in the parameter structure.

- `CONST` is used to copy values from `S` to a
  local variable.
- `VAR` copies the address of a variable in `S` to
  a local pointer.
- `ARRAY` copies the address of the first element of an array
  in `S` to a local pointer.

### The Laws of Devices

Beatbox allows simulations to be run on distributed-memory parallel
machines using MPI. Many devices can be run in parallel without
special adaptation, but it’s difficult to tell exactly what will and
won’t work in parallel. The ‘Laws of Devices’ below describe how a
device should behave in order to ensure its safety in parallel. If
these rules aren’t followed, there is a very real chance that your
device will crash horribly in parallel, or worse, quietly produce
incorrect results. Please read and stick to them.

1. A device must only alter layers of `New` in its space,
   at points in its space.
2. All operations on `New` must be commutative — the order
   of operation cannot be assumed.
3. Aside from the permitted regions of `New`, a device may
   only alter the values of local variables or those in its parameter
   structure.
4. Assignments made to a device’s parameter structure must not be
   derived from data in `New`.
5. Assignments made to a device’s parameter structure must not be
   derived from data in its `Space` structure.
6. Assignments made to a device’s parameter structure must not be
   derived from local minima or maxima
   (e.g. `local_xmax`).
7. Assignments made to a device’s parameter structure must not be
   derived from a random number generator.
8. From any point in its space, a device may not reference points in
   `New` beyond `{x ± 1 ,y ± 1 ,z ± 1}`.
9. A device that references neighbouring points in `New`
   must put the `DEVICE_REQUIRES_SYNC` macro in
   its `Create` function.
10. From any point in its space, a device may not reference points in
    `Geom` beyond `{x ± 1 ,y ± 1 ,z ± 1}`.
11. Files accessed from a device must only be read.

If you’re planning on making a device that needs to do something
forbidden by the laws above, it will need to be explicitly
parallelised to run in parallel. Please consult your local MPI
expert. In the meantime, you can enable the device as sequential only,
by going to `devlist.h` and changing the `D`
before your device’s name to an `S`. It’s now one of the
naughty devices.

## Specification of Ionic Modules

### Overview

The ionic modules split the system of equation describing the kinetics
of the reaction system according to their type into:

- Hodgkin-Huxley type gating variables
  - dependent on membrane voltage (tabulated)
  - dependent on other variables (non-tabulated)
- Markov chain models
- "other" (non-gating) variables

Ionic modules are solved using ruslarsen device, which allows to
exploit specific properties of the specific types of variables by
using exponential integration method. In particular it allows using
Rush-Larsen method for gating variables and Matrix Rush-Larsen method
for Markov chain variables.

### Data Structure of Ionic Modules

The device for integration of ionic modules (rushlarsen device) assumes
the data structures as shown on the Figure 4. The top level structure
`STR` contains an element `I` which refers to an underlying structure
`ionic_str`. The structure `ionic_str` points to `channel_str` called
`channel` which contains Markov chains. Each Markov chain is divided
to a number of subchains of type `subchain_str` to which the
`channel_str` point through the element named `subchain`.

Figure 4: Data structures in rushlarsen device. Elements accepted from `BBScript` (red font); elements specifying underlying substructures (blue font, with arrow); new elements introduced for the implementation of Markov chain models (gray background).

Some elements of the rushlarsen data structure `STR` can be
provided through parameters of `BBScript` (in red
on Figure 4). The name of the parameter
in `BBScript`, which initialises an element of the
structure, is normaly identical to the variable name of the element
(except `p` initialised by `par`
parameter). Most of the parameters are not required
in `BBScript`, and when they are missing the element adopts
its default value.

The detailed information about the elements initialised from
`BBScript` can be found
in this Table. The other elements of
the data structures are used to participate on internal tasks of the
rushlarsen device such as hold intermediate results, look up tables,
or translation a `BBScript` variables in a different type.

### C-Preprocessing with Function-Like Macros

The function-like macros used in BeatBox are normaly in a format
similar to `_(<code1>,<code2>)`, where `<code1>`, and `<code2>` are
pieces of code. The function like macros are processed by `C`
preprocessor (cpp) according to the definition of a particular
macro. An example of such definition is:

```
#define _(variable,initial) var_##variable,
#import "<filename>.h"
#undef _
```

where the string `var_` is concatenated with the first argument called
`variable` (through cpp operator `##`). During the preprocessing stage
of the compilation the cpp processes the code after the definition
(for instance from the `#import "<filename>.h"` files) and substitute
each construct a defined expression. For example a code definiting
initial value of membrane voltage: `_(V,-80.0)` would be substituted
by =var_V_,=.

The following code listing illustrates the idea of expansion of
function-like macros included from header files. This case shows for
enumeration of all dynamical variables:

```
/* Enumerate the dynamical ("state") variables within the vector
   of dynamical variables */
enum {
  #define _(variable,initial) var_##variable,
  #include "<ionic>_other.h"
  #include "<ionic>_ngate.h"
  #include "<ionic>_tgate.h"
  #undef _
  NV            /* total number of variables */
};
```

Notice, that due to the use of enumeration the `var_V` can be used
interchangeably with a number corresponding to the position position
of specific function-like macro (starting from `0`). The value of `NV`
now corresponds to the total number of dynamical variables. The macro
`NV` is required by the ionic module. In the same fashion we define
other macros such as number of "other" (`NO`), tabulated (`NT`) and
non-tabulated gating variables (`NN`), as well as number of tabulated
transition rates (`NTAB`).

The counting and initialisation of variables can be done through
including of separate header files named `<ionic>_<type>.h` into the
main file (`<ionic>.c`). Here `<type>` stands for `other` ("other"
variables), `ngate` (non-tabulated gating variables), `tgate`
(tabulated gating variables), `par` (parameters), `fun` (other
voltage-dependent tabulated functions).

### IONIC\_CREATE\_HEAD(<ionic>) and IONIC\_CREATE\_TAIL(<ionic>)

The `IONIC_CREATE_HEAD(<ionic>)` and `IONIC_CREATE_TAIL(<ionic>)`
macros define a `IonicCreate` `C`-function. Once the macros have been
expanded the name of the `C`-function becomes `create_<ionic>`. The
`create_<ionic>` is called only once and its purpose is to alocate
memory of the data structures to the default values or to the values
provided from the `BBScript`.

The Markov chains are defined within this function through a template
macro `SUBCHAIN(fun_tr, index, min, max, incr, sc)`. This assigns the
transition rates function `trans_rates_mat`, the index of the control
(independent) variable for tabulation `i_control`, the minimum `tmin`,
and the maximum `tmax` limits of the control variable in the table,
the tabulation step `tincr` on the corresponding tabulation scale and
the scale of the tabulation `scale`. The predefined macro also
calculates the number of subchains (`num_sub`) automatically.

### IONIC\_FTAB\_HEAD(<ionic>) and IONIC\_FTAB\_HEAD(<ionic>)

The `IONIC_FTAB_HEAD(<ionic>)` and `IONIC_FTAB_HEAD(<ionic>)` macros
define `IonicFtab` `C`-function which computes the tabulated
transition rates (voltage dependent) of gating variables. The
dynamical equations of the gating variables have a standard form, so
the time derivative can be inferred from the known form and given
values of transition rates.

### IONIC\_FDDT\_HEAD(<ionic>,NV,NTAB,NO,NN) and IONIC\_FDDT\_TAIL(<ionic>)

The `IONIC_FDDT_HEAD(<ionic>,NV,NTAB,NO,NN)` and
`IONIC_FDDT_TAIL(<ionic>)` macros define `IonicFddt` type
`C`-function. This `C`-function computing the time derivative of the
non-gating ("other") variables as defined on the right hand side of
the equation and computing the non-tabulated transition rates
(dependent on "other" variables).

Once the macros have been expanded the name of the
`C`-function becomes `fddt_<ionic>`. The variables in the template of
the macros are the numerical value of total number of variables `NV`,
the number of voltage-dependent tabulated functions `NTAB`, the number
of non-gating "other" variables `NO`, and the number of
"non-tabulated" gating variables `NN`.

### CHANNEL\_TR\_MATRIX(<ionic>\_<subchain>)

The `C`-functions to fill transition rates matrices of Markov chains
are generated using a macro `CHANNEL_TR_MATRIX(<ionic>_<subchain>)`
which defines `TransRatesMat` `C`-function. After the macro has been
expanded the name of the `C`-function becomes `<ionic>_<subchain>`.

The macro `_RATE` helps to generate `TransRatesMat` functions that
fills the transition rates matrix of a subchain.

```
CHANNEL_TR_MATRIX(<ionic>_ical){
  #define v u[0]
  #define _(n,i)
  #define _VFUN(name,expression) real name=expression;
  #define _RATE(from,to,direct,reverse)     \
    TR_MAT(<channel>,from,to,direct,reverse)
  #include "<ionic>_<channel>.h"
  #undef _RATE
  #undef _VFUN
  #undef _
  #undef v
  return 1;
}
```

The transition rates matrix is constructed from macro
`_RATE(from,to,direct,reverse)`, where as the name suggest the first
two arguments specify two states `from`, `to` between which the
transition rates are defined in `direct`, and `reverse` expressions.

The diagram Figure 5 ilustrates the process of filling the
entries of the transition rates matrix. The pannel (a) shows the form
of the function-like macro with arguments. The arguments are
substituted during the preprocessing phase of the compilation with the
names of the states and the expressions for the transtion rates. A
diagram of a part of the Markov chain described by given function-like
macro is shown on the pannel (b). The panel (c) shows the
corresponding entries in the transition rates matrix filled with the
expressions for the transition rates.

Figure 5: Construction of the transition rates matrix: (a) a form of the function-like macro with arguments; (b) a diagram of the Markov chain (with additional states S_1_ and S_2_) corresponding to the function-like macro; (c) part of the transition rates matrix constructed from the function-like macro.

In the figure, the possition of the state is given by `<channel>_from`
and `<channel>_to` where the `from` and `to` are substituted with the
arguments of the function-like macro.

### Minimalist Example of Ionic Module

A minimalist example of ionic module including Markov chains can be found in

```
src/hh52m.c
```

# Visualisation

Beatbox has the functionality to output `ppm` files which
allow easy visualisation, especially of large data sets. The
visualisation can be further improved using `BBXVIEW`
package.

To convert `ppm` to `jpg/png`, use something
like this in a shell script from `netpbm` and usually 0. If
voltage is being output to the `ppm` files, then the:

```
for (( i = $start; i <= $finish; i++))
do
   ppmtojpeg out/ord2_${i}.ppm > jpegs/ord2_${i}.jpg
done
```

Further, to make an animation,
use ImageMagick using the
convert command:

```
convert -delay 20 -loop 0  sphere*.gif animatespheres.gif
```

A shell script to handle these tasks will have the following contents:

```
#!/bin/bash
for (( i = $start; i <= $finish; i++))
do
   ppmtojpeg out/ord2_${i}.ppm > jpegs/ord2_${i}.jpg
done
#
convert -delay 20 -loop 0  sphere*.gif animatespheres.gif
```

You will need to make this shell script executable. For example, if
you save it as myscript.sh, then you need to do the following:

```
chmod 766 myscript.sh
```

After this, you can run your script like any other program from
the command line as:

```
./myscript.sh
```

`ParaView` can be used to make animations using the
3D `ppm` data. To do this, first run the
script ppm\_2\_paraview. This perl script
needs the inputs bgr bgg bgb channel threshold
`filename.ppm` where the rgb values are from the bbs file,
or default values `(255 255 255)`. The channel is again
from the bbs file threshold is usually around 127. Once the vtk files
are produced in this manner, use paraview to prepare the visualisation
interactively using the first file. An output to animation will then
produce the desired
`jpeg`s / `tiff`s
/ `png`s. Use `convert` from ImagemMagick to make
a low maintainence animated `gif` that can be put into
powerpoint or web pages.

# Advanced

call graphs to show how the code works for developer's.

# How to implement a CellML cell model into Beatbox

Following is a method involving minimal manipulation of CellML cell
model code to incorporate it into Beatbox format. We use the
OHara-Rudy 2011 model as an example to illustrate the method.

## Step 1: Get the CellML file

Published cardiac and other simulation models are distributed by means
of the CellML Project website, that can be found
here: CellML Project. In
particular, the cell models are listed in the
repository: CellML
Repository. The desired CellML file should be downloaded from the
repository. The file that we are looking for is usually has an
extension of `.cellml`. As an example, the OHara-Rudy (ORd)
cell model file looks like this: ORd
CellML file. There are other formats in which the cell model may
be provided (C, C++, MATLAB,...) and the best location to get the
CellML file is from the provided tar.gz download all files section.

## Step 2: Convert the CellML file to C code

The information in the CellML file obtained as in Step 1 needs to be
converted into suitable C format. In principle, the converted codes
as delivered by the CellML website may be sufficient. However, to
obtain all the information required to construct the cell model in
Beatbox format (i.e. initial conditions and the algebraic-differential
equations for the model dynamics) is ideally obtained by means of COR
software. COR (Cellular Open Resource) is primarily a Windows
software, but may be installed on Linux and MAC-OS if the operating
system is 32 bit or if there are 32 bit emulators available. As of the
date of this document, a Linux variant is also being developed. So the
next thing to do is download and
install COR. Once it is
installed, open the CellML file that you downloaded in Step 1 and
export it to C. The exported files are a `.c` and
a `.h` with the same name as the CellML file. For example,
the ORd exported files
are:Ohara\_Rudy\_2011.c
and Ohara\_Rudy\_2011.h.

## Step 3: Define the constant modelling default parameters

The header file as exported in Step 2 simply declares all constants
and variables. The format that the declarations appear in the header
file depend on the original CellML file and how the model authors
provided the original codes to CellML. In the C file
(`Ohara_Rudy_2011.c` in our case), there is hopefully an
annotated section (called "Constants" or similar) that shows the model
constants and this section should be identified. It should be borne in
mind that these constants should be simply numerical assignments to
place holders, i.e. a formula should not appear on the right hand side
of these assignments. Further, due to the Beatbox format, these
modelling constants should all be real (i.e. `float`
or `double`) numbers. All derived constants and integer
constants are handled otherwise (see below).

Take this list of constants and put them in a file called, say
`ord_par.h`. The format of the list of constants should be further
manipulated such that Beatbox macro's can do what they should do. The
final `ord_par.h` should look like
this: ord\_par.h.

## Step 4: Define the initial conditions to the model

A `void init(void)` (or similarly named function) is
provide in the exported C file. Contents of this function without any
alterations should be copied to another file. In the ORd example, we
choose to call this file `ord_init.h`. Note that none of
the contents of the initialisation have been altered. The exported C
file probably (hopefully) uses a vector to maintain values of dynamic
variables, and that should also be retained to reduce manipulation of
original code. The prepared `ord_init.c` file can be found
here: ord\_init.h. Note that unlike in the
parameter file of Step 3, the code in the initialisation has not been
manipulated at all.

## Step 5: Define the actual model calculation

After having defined the constant real parameters and assigned initial
values to the dynamic variables vector, we now construct the code that
will be used for the actual cell model integration. This step starts
with identifying the function called void compute(double t) or similar
in the exported C file. The contents of this function should be
imported into another header file, say called ord\_step.h. You may
notice that in this code, there are several derived variables
(e.g. currents, fluxes,...), integer constants, etc. These derived
variables are typically declared as "Computed Variables" or with
similar annotation. These declarations should be put at the top before
the model code. The final `ord_step.h` look like this:
ord\_step.h. Note that no integration (forward
Euler or otherwise) is included in this `ord_step.h` - this
is simply the code for computing the next time step increment. A
vector called dY[] may also be found in this code. We will deal with
this in a bit.

## Step 6: Define a variable to deal with the device in ord.on

A constant variable needs to be defined to tell several of Beatbox
macros about the new cell model. For example, in case of the ORd,
create a file ord.on with the following line of code:

```
#define ORD 1
```

You can see the file: ord.on.

## Step 7: Update rhslist.h

The list of RHS of cell models needs to be updated. This list is in:

- `Beatbox/src/rhslist.h`

of the package. Code, similar to what is shown below, should be added
to this file:

```
#if ORD
   D(ord)
#endif
```

Explanations on the meaning of the macro D, the choice of names
etc. are to be found in the developers guide.

## Step 8: Define the wrapper for the model simulation

The main C wrapper that initialises, declares local placeholders, and
provides the cell model code to the Euler solver is given a consistent
name. For example, in case of ORd, it is simply called ord.c. This
wrapper contains headers, macros, and basically includes the code from
the above files.

- To begin with, the file contains several generic headers, and if
  required, the cell model specific headers.
- Then the number of dynamic variables should be defined, typically
  using a `#define N` directive.
- Next, a structure is declared that includes the parameter file, and
  other modelling parameters. For cell models, this is typically the
  (constant) time step and the stimulus current variable. Using
  the `RHS_CREATE_HEAD` macro, we initialise members of the
  structure with constant parameters and initial values using the files
  that we created above. In the `RHS_RUN` macro, first the
  parameters are declared as part of the structure `S`. Since
  the local vector of variables is probably called something different
  from `u[]` (probably `Y[]`), we make that
  association here.
- The next thing to do is simply include the `_step.h`. If
  required, add the current stimulus to the appropriate ODE variable and
  then the macro can be closed.

## Step 9: Update the Makefile.am

There is a `Makefile.am` in the `Beatbox/src`
directory. Add entries for `.on`, `_step.h`,
`_init.h`, `_par.h`, and `.c` files
that you have just created. Save, do a distclean, configure, and make
again. Hopefully, it all compiles. To start using the cell model, make
a `bbs` file as shown in the template below.

## Step 10: Make a bbs file to simulate the AP

A `bbs` file template: ord0.bbs:

# A Practical Guide for Automake if you add new modules

The inclusion of cellular modules into BeatBox requires changes of BeatBox source files and files of the GNU build system of BeatBox `configure.ac` (the suffix is an abreviation from Autoconf) and `Makefile.am` (the suffix is an abreviation from Automake). Those files are used to create configuration `configure` and build script `Makefile`.

The building of a new ionic module starts by including its name into the `ioniclist.h` in a form `D(<ionic>)`.

```
D(hh52)
  D(hh52m)
```

The building of a new rhs module starts by including its name into the `rhslist.h` in a form `D(<rhs>)`.

```
#if HH
  D(hh)
#endif
```

The rhs modules also require to add a macro definition to the file `on.h` and generate a new file `<rhs>.on` with the same macro. This macro reads as:

```
#define HH 1
```

The main module file contains formal definition of the model. It is expected in the file `<name>.c`. The time stepping devices require the module module to initialise specific structures and functions.

All files used in for the implementation of those modules have to be listed within `common_sources` variable in `src/Makefile.am` in BeatBox root directory:

```
common_sources = \
  hh52.c hh52_other.h hh52_tgate.h hh52_par.h hh52_ftab.h\
  hh52m.c hh52m_ik.h hh52m_ina.h\
  hh.c \
  HH.on \
  hh.h \
```

this file maintains the list of the source files. The Automake takes care of generating the =Makefile=s specific for build on a target computer architecture, that BeatBox executable is aimed for. This allows for portability of the code to a large variety of different machines.

The template files `Makefile.am` and `configure.ac` (not modified in this example) are processed to generate and install corresponding `configure` and `Makefile` in the appropriate directories. This is done by a command

```
autoreconf -fi
```

in the root directory of BeatBox (`-f` for force, `-i` for install).

# Bash / perl scripting for increased Beatbox functionality

Content to be added.

# Beatbox Device Reference

## activation

### Overview

This is a computational device, which computes and stores
activation times at all points in the grid, within its space. The
activation time at a point of the grid is defined as the most recent
moment when the value of a certain field at that point has crossed a
given value in a given direction. The typical intended use is that the
"certain field" is the transmembrane voltage. The activation times are
in terms of the integer counter `t`, but their values are
real, and defined by linear interpolation between the current and the
previous value of the selected field at the given point. If activation
times in terms of the model time are required, these values should be
properly interpreted, typically just multiplied by the time
step. Since the device needs the current and the immediately preceding
values of the given field, it should be run at continuous time
intervals for correct work.

### Parameters

| Type | Name | Description |
| --- | --- | --- |
| `int` | `v0` | The layer containing the field used for definining the activation time, e.g. the transmembrane voltage layer. |
| `int` | `v1` | The layer used for storing the computed activation times. Must be different from `v0`. |
| `int` | `vd` | An auxiliary layer, used for storing previous values of the field. Must be different from both `v0` and `v1`, and must not be modified by any other devices. In particular, it should be outside the range of layers used by the cell model(s). |
| `real` | `threshold` | Activation is the event when the selected field crosses this value, in the direction defined by the next parameter, `sign`. |
| `int` | `sign` | Only the sign of this parameter matters. If positive, activation is defined when the selected field value crosses the threshold value upwards; if negative, activation is when the value crosses the threshold downwards; if zero, then crossing in either direction will be counted as activation. |

All parameters are mandatory.

### Typical Syntax

```
activation v0=[Volt] vd=[p] v1=[a] threshold=Vact sign=1;
```

where string macro `[Volt]` contains the number of the
layer used for storing transmembrane voltage, `[p]` and
`[a]` are layers outside the kinetic model, where layer
`[a]` will contain the most recent activation times in all
points through which the excitation wave passed at least
once. Excitation wave is considered to have passed through a point
when the voltage at that point crossed the value given by the
previously assigned k-variable `Vact` upwards.

### Instances in Example Scripts

```
beatbox/data/scripts/sequential/TenTusscherPanfilov_modelS/ttp06/benchmark.bbs
```

This script can be run e.g. using the following command:

```
Beatbox_SEQ benchmark.bbs 0.5.bbg 0.5 0.05 0.5_0.05.dat
```

where `0.5.bbg` is the geometry file, 0.5 and 0.05 are
space and time discretization steps, and `0.5_0.05.dat` is
the output file where the results of the benchmark will be stored.
More results will be also stored in files in the subdirectory
`0.5_0.05.dat.dir`. See the `Makefile` and
`eport.pdf` in that directory for more details.

## adi3d

### Overview

This is a computational device, which performs an
alternating-direction implicit (ADI) timestep for an isotropic
diffusion operator with Neumann boundary conditions in box geometry in
3D. We use the Brian algorithm as described e.g. in Carnahan et
al. book for implicit solution of heat equation (eqn. 7.56 on page
453).

### Parameters

| Type | Name | Description |
| --- | --- | --- |
| `int` | `v0` | The layer containing the diffusive field. |
| `int` | `v1` | An auxiliary layer, used for intermediate ADI calculations. Must be different from `v0`. |
| `real` | `ht` | Time step. |
| `real` | `hx` | Space step. |
| `real` | `D` | Diffusion coefficient. |
|  |  |  |

All parameters are mandatory.

### Typical Syntax

```
 adi3d v0=[u] v1=[aux] D=D hx=hx ht=ht;
```

where string macro `[u]` contains the number of the
layer used for storing the diffusive field (say the transmembrane
voltage),
`[aux]` is a layer outside the kinetic model, and
`D`, `hx` and `ht` are k-variables
for the diffusion coefficient, space step and time step.

### Instances in Example Scripts

```
beatbox/data/scripts/sequential/Barkley_model/implicit.bbs
```

This script can be run using the following command:

```
Beatbox_SEQ implicit.bbs
```

and the directory containing this script also contains the
pre-requisite file `bkl1.rec`.
It also contains Perl script `view.pl`.
To view the result of simultion,
you say in that directory:

```
./view.pl implicit.dir
```

on the command line.

## clock

### Overview

Prints the current integer timestep to `stdout`.

### Parameters

| Type | Name | Description |
| --- | --- | --- |
| `int` | `when` | rate of output |
| `int` | `col0` | starting column of graphics. |
| `int` | `row0` | starting row of graphics. |
| `int` | `color` | Color code of line graphics. |

### Typical Syntax

The clock device is mainly used during run time graphics simulations.
The typical syntax of `clock` device is as follows.
The clock device is mainly used during run time graphics simulations.
The typical syntax of `clock` device is as follows.

```
clock when=often color=WHITE row0=10 col0=20;
```

### Instances of clock in Example Scripts

```
beatbox/data/scripts/sequential/FitzHughNagumo_model/fhn0.bbs
beatbox/data/scripts/sequential/FitzHughNagumo_model/fhn1.bbs
beatbox/data/scripts/sequential/FitzHughNagumo_model/fhn2.bbs
```

The inputs, outputs, and methods of running for fhn0-fhn2.bbs
have been described in previous sections.

## ctlpoint

### Overview

Saves the entire simulation state for disaster recovery, or to
facilitate a branching point in long simulations.

### Parameters

| Type | Name | Description |
| --- | --- | --- |
| `str` | `file` | Control point file to be restored from / saved to. |
| `int` | `enrich` | Can the restored control point define k variables not present in the script. |

## d\_dt

### Overview

Computes the time derivative of a layer (dynamical variable) at a
given location.

### Parameters

| Type | Name | Description |
| --- | --- | --- |
| `real` | `ht` | time step |
| `int` | `vd` | Dynamical variable for which derivative is sought. |

### Typical Syntax

d\_dt ht=ht vd=1;

### Instances in Example Scripts

```
beatbox/data/sequential/FitzHughNagumo_model/fhn2.bbs
```

The inputs, outputs, and method of simulation for fhn2.bbs have been
described above.

## diff

### Overview

Computes the Laplacian (or Laplacian-Beltrami for anisotropic case),
with Neumann (resp. non-flux) boundary conditions.

### Parameters

`diff` must be used with the default
space. The `v0` space parameter is used to indicate the
diffused variable, usually the transmembrane
voltage. The `v1` space parameter is used to indicate the
layer in which the Laplacian should be stored.

| Type | Name | Description | Isotropic or Anisotropic |
| --- | --- | --- | --- |
| `int` | `v0` | The layer containing the diffusive field. | Both |
| `int` | `v1` | The layer that will contain the Laplacian of the diffusive field. Must be different from `v0`. | Both |
|  |  |  |  |
| `real` | `hx` | Space step. | Both |
| `real` | `D` | Isotropic diffusion coefficient. | Isotropic only |
| `real` | `Dpar` | Anisotropic diffusion coefficient parallel to the fibres. | Anisotropic only |
| `real` | `Dtrans` | Anisotropic diffusion coefficient in the transverse direction, perpendicular to the fibres. | Anisotropic only |

### Typical Syntax

The typical syntax of `diff` device is as follows. Assuming
that the diffusing layer is `diff_layer` (a number) and the
layer in which the Laplacian is to be stored
is `lap_layer`, the syntax for the isotropic diffusion is:

```
diff v0=diff_layer v1=lap_layer D=D hx=hx;
```

where uniform diffusion is used throughout the tissue. In case of
anisotropic diffusion, the syntax changes to account for diffusion
parallel to the fibres (signified by `Dpar`) and transverse
to the fibres (signified by `Dtrans`) and is:

```
diff v0=diff_layer v1=lap_layer Dpar=D Dtrans=D/4 hx=hx;
```

### Instances of diff in Example Scripts

All example scripts can be found in data/scripts of the package
distribution. Examples of isotropic diffusion can be found in the
following:

```
beatbox/data/scripts/sequential/FitzHughNagumo_model/fhn1.bbs
beatbox/data/scripts/sequential/FitzHughNagumo_model/fhn2.bbs
```

and for the anisotropic case can be found in

```
beatbox/data/scripts/sequential/LRD_model/LRD_ffr.bbs
```

The inputs, outputs, and method of simulation
for `fhn1.bbs`, `fhn2.bbs`, and
`LRD_ffr.bbs` have been described above.

## diffstep

### Overview

As `diff` device, but in addition to calculating the
diffusion operator ∇*D*∇*V*,
it implements the explicit Euler timestep with this operator for the
diffused variable. That, is, if the diffusive variable is *V* and
the time step is `ht`, then after this
device's work, the field in the `diff_layer` will be
incremented by `ht` times the Laplacian.

*V*(*x*,*y*,*z*,*t*)
 → 
*V*(*x*,*y*,*z*,*t*+*h*_*t*_)
 = 
*V*(*x*,*y*,*z*,*t*)
+
*h*_*t*_
∇(*D (*x*,*y*,*z*)*∇ *V(*x*,*y*,*z*,*t*)* )

Hence this device can be used to make the diffusion substep in an
operator-splitting scheme, without the need to transfer the values of
the Laplacian to the timestepper for the reaction term.

### Parameters

`diffstep` must be used with the default
space. The `v0` space parameter is used to indicate the
diffused variable, usually the transmembrane
voltage. The `v1` space parameter is used to indicate the
layer in which the Laplacian should be stored.

| Type | Name | Description | Isotropic or Anisotropic |
| --- | --- | --- | --- |
| `int` | `v0` | The layer containing the diffusive field. | Both |
| `int` | `v1` | The layer that will contain the Laplacian of the diffusive field. Must be different from `v0`. | Both |
| `real` | `ht` | Time step. | Both |
| `real` | `hx` | Space step. | Both |
| `real` | `D` | Isotropic diffusion coefficient. | Isotropic only |
| `real` | `Dpar` | Anisotropic diffusion coefficient parallel to the fibres. | Anisotropic only |
| `real` | `Dtrans` | Anisotropic diffusion coefficient in the transverse direction, perpendicular to the fibres. | Anisotropic only |

### Typical Syntax

is very similar to `diff`, only it should contain
`ht=` definition; otherwise parameter `ht` will
be assume the default value of zero and the work of this device will
be identical to that of `diff` only slightly less
efficient. So, assuming
that the diffusing layer is `diff_layer` (a number) and the
layer in which the Laplacian is to be stored
is `lap_layer`, the syntax for the isotropic diffusion is:

```
diffstep v0=diff_layer v1=lap_layer D=D hx=hx ht=ht;
```

where uniform diffusion is used throughout the tissue. In case of
anisotropic diffusion, the syntax changes to account for diffusion
parallel to the fibres (signified by `Dpar`) and transverse
to the fibres (signified by `Dtrans`) and is:

```
diffstep v0=diff_layer v1=lap_layer Dpar=D Dtrans=D/4 hx=hx ht=ht;
```

Notice that if a positive value for `ht` is given then after this
device's work, the field in the `diff_layer` will be
incremented by `ht` times the Laplacian, and the layer
`diff_layer` may be recycled for another purpose, say as a
Laplacian layer for another diffusion device.

CAVEAT: in the (straightforward) operator-splitting schemes, the value of parameter
`ht` in this device should, obviously, be equal to the time
step used in the devices that do other substeps (unless the contrary
is required by a more sophisticated operator-splitting scheme
e.g. Strang splitting, where some of the substeps are done with time
step `ht/2`).
If `ht=0` (which is the default value), then the contents
of the layer `diff_layer` will remain unchanged, and the
only result of the work of the `diffstep` device will be
the values of the Laplacian stored in the layer
`lap_layer`, i.e. the outcome will be identical to that of
a `diff` device.

### Instances of diff in Example Scripts

All example script can be found in data/scripts of the package
distribution:
following:

```
beatbox/data/scripts/sequential/CRN/compare1D.bbs
```

This script is considered in detail in the section for the
`rushlarsen` device.

## dump

### Overview

Writes the contents of the simulation medium within its space to a
binary file.

### Parameters

| Type | Name | Description |
| --- | --- | --- |
| `str` | `file` | Relative path to the dump file. |
| `int` | `append` | Should the file be appended to? |

### Typical Syntax

The typical syntax of `dump` device is as follows. Assuming
that the dynamical layers are between `ode_layer0` (a
number) and the layer `ode_layer1` the syntax for the dump
device is:

```
dump when=end v0=ode_layer0 v1=ode_layer1 append=0 file=my_dump_file.dmp;
```

### Instances of dump in Example Scripts

All example scripts can be found in `beatbox/data/scripts` of the
distribution package. Examples of dump device can be
found in the following:

```
beatbox/data/scripts/sequential/LRD_model/LRD_ffr.bbs
```

The simulation state saved in such a manner may be used to continue
the simulation using the `load` device. The inputs,
outputs, and method of simulation for `LRD_ffr.bbs` have
been described above.

## elliptic

### Overview

Solves an elliptic problem (Poisson equation) for the
Laplace-Beltrami operator corresponding to the fiber direction field
of the current geometry and given diffusion coefficients along and
across fibers (as in `diff` device). The primary intended
use is calculation of the extracellular (or intracellular) potential
field for a given transmembrane field in the bidomain model. The
method of solution is Full Multigrid with vertex-centered
restrition/prolongation operators with bi/tri-linear interpolation,
and (multicoloured) Gauss-Seidel or Jacobi smoother. Can be used
either for Dirichlet boundary conditions, where boundary values are
those that are just outside the device box, or Neumann boundary
conditions. In the latter case, this will require an extra "pinning"
condition, to select a unique solution out of infinitely many: the
solution at a given point should have a given value.

### Parameters

| Type | Name | Default | Description |
| --- | --- | --- | --- |
| `real` | `Dpar` | - | Anisotropic diffusion coefficient parallel to the fibres. |
| `real` | `Dtrans` | - | Anisotropic diffusion coefficient in the transverse direction, perpendicular to the fibres. |
| `real` | `hx` | - | Space step. |
| `real` | `tolerance` | - | One step tolerance: if maximal pointwise change of solution during one iteration, the iterations are considered converged. |
| `int` | `maxiter` | - | Maximal number of final iterations after the Full Multigrid cycle. |
| `int` | `maxiter` | - | Maximal number of final iterations after the Full Multigrid cycle. |
| `int` | `pin` | 0 | If nonzero, then a pinning condition will be engaged. |
| `int` | `xpin` | - | x-coordinate of the pinning point. |
| `int` | `ypin` | - | y-coordinate of the pinning point. |
| `int` | `zpin` | - | z-coordinate of the pinning point. |
| `real` | `upin` | - | The pinning value. |
| `int` | `upper_level` | -1 | The maximal number of lavels in multigrid. The original (basic) grid is at level 0, and each next level has the number of points in each dimension approximately twice smaller. When this parameter is 0 or -1, straightforward iterations at basic level (i.e. without multigrid) are performed; only for `upper_level=-1` they are performed right with the `New` array, whereas for `upper_level=0` the right-hand side and solution data are copied to a from the separate structures designed for multigrid. |
| `string` | `smoother` | `GS` | Name of the smoother used at each iteration. This can be either `Jacobi`, or `GS` for Gauss-Seidel. |
| `string` | `transfer` | `correct` | The method of transfer of the problem from one level to the other. Can be either `transfer` or `correct`. In case of `transfer`, all levels compute the same problem but at different discretization. In case of  `correct` , higher levels compute corrections to the lower-level solutions. |
| `real` | `damping` | `1.0` | Relaxation coefficient. Normally `damping=1`. If `0<damping<1`, the iterations are done half-way; use `damping&gr1` for the successive over-relaxation (not recommended when multigrid is engaged). |
| `real` | `delta` | `1.0` | Tolerance factor for multigrid. The tolerance of iterations of the next level is `delta` times bigger than the tolerance of the previous level. |
| `int` | `FMGlevel` | `upper_level` | The range of the Full Multigrid (Nested) iterations. |
| `int` | `vcycles` | `1` | The number of V-cycles per one nested iteration. |
| `int` | `preiter` | `maxiter` | The number of pre-iterations in a V-cycle. |
| `int` | `postiter` | `maxiter` | The number of post-iterations in a V-cycle. |
| `int` | `postiter` | `maxiter` | The number of post-iterations in a V-cycle. |
| `int` | `tol0, tol1, ...` | (same from prev level) | parameter `tol` can be specified for individual multigrid levels 0, 1, ... |
| `int` | `preiter0, preiter1, ...` | (same from prev level) | parameter `preiter` can be specified for individual multigrid levels 0, 1, ... |
| `int` | `postiter0, postiter1, ...` | (same from prev level) | parameter `postiter` can be specified for individual multigrid levels 0, 1, ... |
| `str` | `debug` | none | Relative path to output file for debugging purposes. If `stdout`, will print to screen. |
| `str` | `profile` | none | Relative path to output profiling information. This will measure separately time spent at each of the levels of the multigrid (and noticeably slow down computations). Now is available only in sequential mode. If `stdout`, will print to screen. |
|  |  |  |  |

### Typical Syntax

see in the example of use referred to below.

### Instances of `elliptic` in Example Scripts

An examples of `elliptic` device can be found in the following:

```
beatbox/data/scripts/sequential/ZFK_model/zfkmod2.bbs
```

It does not require command-line parameters so can be run e.g. by

```
Beatbox_SEQ zfkbid2.bbs
```

This calculates a bidomain model with the one-component Zeldovich-Frank-Kamenetsky (a.k.a.
Nagumo) kinetics, with initial and (Dirichlet) boundary conditions
chosen in such a way as to accommodate an exact analytical solution
for a front propagating at the angle of 30 degrees wrt the x-axis.
For comparison, there is a file `zfkmod2.bbs` script in the
same directory, which solves a similar problem in the monodomain
formulation.

## euler

### Overview

General pointwise ODE stepper for the rhs modules.

### Parameters

| Type | Name | Description |
| --- | --- | --- |
| `real` | `ht` | Timestep duration. |
| `str` | `ode` | Name of the RHS module to use. |
| `int` | `rest` | If nonzero, the device will make so many steps with the given model to find an approximation of the initial conditions, and then fill the whole device space with this initial condition. |
| `codeblock` | `par` | The parameters of the RHS module; their list depends on the module. The codeblock contains k-assignments of the form `<parameter>=<k-expression>`, separated by blank spaces. |

The parameter `ht` defines a duration of one time step used in forward
Euler calculations.

The parameter `ode` is a name of the rhs module that implements the
cellular kinetics. This module is used for the simulation. The `ode`
model has to follow the rhs format.

The parameter `rest` is used if the device determines the initial
conditions of the dynamical variables by a steady state
approximation. The device runs the simulation for the number of steps
specified in `rest` using the initial conditions as specified in the
code of `rhs` module.

Model dependent parameters of rhs module are passed to euler device
through the `par` parameter in `BBScript`. The assignment has to
follow the format `{<parameter>=<value>}` (within curly brackets `{}`
as shown). When multiple parameters are assigned, their definition
must be all given within the same curly brackets and each assignment
must be separated by a blank space (such as space, or newline).

### Typical Syntax

The typical syntax of euler device is as follows. Assuming that the
ODE layers are between `ode_layer0` (a number) and the layer
`ode_layer1`, the ODE module (i.e. cell model) that you are using od
ode\_module_, and the user defined model parameters are defined in bbs
parameters p1, and p2, the syntax is:

```
euler name=name_to_device_call v0=ode_layer0 v1=ode_later1 ht=ht ode=ode_module rest=100 par={par1=p1; par2=p2;};
```

You can given the device call a convinient name, `name_to_device_call`
in this case, to refer to your device elsewhere in the bbs script.

### Instances of euler in Example Scripts

All example scripts can be found in `beatbox/data/scripts` of the
package distribution. Examples of euler device can be found in the
following:

```
beatbox/data/scripts/sequential/FitzHughNagumo_model/fhn0.bbs
beatbox/data/scripts/sequential/FitzHughNagumo_model/fhn1.bbs
beatbox/data/scripts/sequential/FitzHughNagumo_model/fhn2.bbs
```

Indeed, all bbs scripts in the examples use the euler device.

## grad2d

### Overview

Computes the 2D gradient at a given x,y,z location.

### Parameters

The only parameter is the space step.

### Typical Syntax

grad2d hx=hx;

### Instances in Example Scripts

```
beatbox/data/sequential/FitzHughNagumo_model/fhn2.bbs
```

The input, output, and simulation method of fhn2.bbs has been described
previously.

## imgout

### Overview

Produces png images of the space. Values from specified layers of the
simulation medium are mapped to colour channels by linear
interpolation using specified minima and maxima. imgout produces a
new, sequentially numbered file every time it is run.

### Parameters

| Type | Name | Description |
| --- | --- | --- |
| `Sequence` | filter | On the fly producing png images. |
| `int` | `r` | Layer of the simulation medium from which the red colour channel should be mapped. |
| `real` | `r0` | Value of the layer assigned to r that should map to 0. |
| `real` | `r1` | Value of the layer assigned to r that should map to 255. |
| `int` | `g` | Layer of the simulation medium from which the blue colour channel should be mapped. |
| `real` | `g0` | Value of the layer assigned to `g` that should map to 0. |
| `real` | `g1` | Value of the layer assigned to `g` that should map to 255. |
| `int` | `b` | Layer of the simulation medium from which the green colour channel should be mapped. |
| `real` | `b0` | Value of the layer assigned to b that should map to 0. |
| `real` | `b1` | Value of the layer assigned to `b` that should map to 255. |

### Typical Syntax

The typical syntax of `imgout` device is as follows.

```
imgout filter="pnmflip -tb | pnmtopng > [outdir]/uvi%07.0f.png" mode="w" r=[u] r0=umin r1=umax g=[v] g0=vmin g1=vmax b=[i] b0=-1 b1=1;
```

### Instances of ppmout in Example Scripts

Examples of ppmout device can be found in the following:

```
beatbox/data/scripts/sequential/FitzHughNagumo_model/fhn2.bbs
```

## k\_clock

### Overview

Prints the current time to `stdout`.

### Parameters

| Type | Name | Description |
| --- | --- | --- |
| `int` | `when` | rate of output |
| `int` | `col0` | starting column of graphics. |
| `int` | `row0` | starting row of graphics. |
| `int` | `color` | Color code of line graphics. |
| `real` | `code` | usually set to t\*dt |
| `string` | `format` | string defining format of output real |

### Typical Syntax

The k\_clock device is mainly used during run time graphics simulations.
The typical syntax of `k_clock` device is as follows.

```
k_clock when=often color=WHITE row0=1 col0=21 code=t*ht format="T=%4.1f ms";
```

### Instances of `k_clock` in Example Scripts

```
beatbox/data/scripts/sequential/FitzHughNagumo_model/fhn0.bbs
```

The inputs, outputs, and methods of running for fhn0.bbs
have been described in previous sections.

## k\_draw

### Overview

Draws the visualisation window.

### Parameters

| Type | Name | Description |
| --- | --- | --- |
| `int` | `col0` | starting column of graphics. |
| `int` | `col1` | end column of graphics. |
| `int` | `row0` | starting row of graphics. |
| `int` | `col1` | end column of graphics. |
| `int` | `color` | Color code of line graphics. |

### Typical Syntax

The typical syntax of k\_draw device is as follows.

```
def str win1 col0=col0 col1=col1 row0=row0 row1=row1 color=15*16+15;
k_draw when=always [win1] color=BLUE*16;
```

### Instances in Example Scripts

```
beatbox/data/scripts/sequential/FitzHughNagumo_model/fhn0.bbs
```

## k func

### Overview

Allows the user to specify user defined functions to be executed as
part of the simulation run.

### Parameters

| Type | Name | Description |
| --- | --- | --- |
| `int` | `restricted` | If this parameter is nonzero, this device will never run in any non-tissue points. |
| `str` | `file` | Path to file for phase distribution. See Beatbox Scripting Guide. |
| `str` | `debug` | Relative path to output file for debugging purposes. If `stdout`, will print to screen. |
| `codeblock` | `pgm` | The function to be executed when the device is run. The codeblock contains k-assignments of the form `<k-variable>=<k-expression>`, separated by semicolons. Users should note that the behaviour of `k_func` changes significantly in relation to the nowhere parameter.When `nowhere=1`, assignments can be made to k variables only. Assignments from the simulation medium or random number generators are disallowed. When `nowhere=0`, assignments can be made to the simulation medium only. It is possible to define intermediate variables in `k_func` programs, using the def keyword. Variables must be initialised before they are used, and are reinitialised on each iteration of the `k_func` program. |

### Typical Syntax

The typical syntax of `k_func` device is as follows.

```
k_func name=timing nowhere=1 pgm={
  begin = eq(t,0);          // 1 when t is equal to zero, 0 otherwise. 
  often = eq(mod(t,10),0);  // 1 if t is divisible by 10, 0 otherwise.
  end   = ge(t,100000);	    // 1 if t is greater or equal than 100000, 
                            // 0 otherwise.
};
```

### Instances in Example Scripts

```
beatbox/data/scripts/sequential/FitzHughNagumo_model/fhn0.bbs
```

## k\_paint

### Overview

`k_paint` is a device for run-time graphics to paint the field using VGA colour palette.

### Parameters

| Type | Name | Description |
| --- | --- | --- |
| `int` | `when` | Output rate |
| `int` | `col0` | starting column of graphics. |
| `int` | `col1` | end column of graphics. |
| `int` | `row0` | starting row of graphics. |
| `int` | `col1` | end column of graphics. |
| `int` | `color` | Color code of line graphics. |

### Typical Syntax

The typical syntax of `k_paint` device is as follows.

```
k_paint when=out nabs=nx nord=ny          // how many abscissa and ordinate values
  col0=col0 col1=col1 row0=row0 row1=row1 // output it into the left panel
  color=WHITE*16+WHITE                    // white borders for the panel
  pgm={                                   // program should calculate colour for given (abs,ord)
                                          // and we take abs=x and ord=y
  col=ifge0(u(abs,ord,0,[u]),LIGHTRED,0)  // red component there if u field is above 0
  + ifge0(u(abs,ord,0,[v]),GREEN,0)};     // and green if [v] field is above 0
```

### Instances in Example Scripts

```
beatbox/data/scripts/sequential/FitzHughNagumo_model/fhn2.bbs
```

## k\_plot

### Overview

Plots the visualised variables.

### Parameters

| Type | Name | Description |
| --- | --- | --- |
| `int` | `col0` | starting column of graphics. |
| `int` | `col1` | end column of graphics. |
| `int` | `row0` | starting row of graphics. |
| `int` | `row1` | end column of graphics. |
| `int` | `color` | Color code of line graphics. |
| `int` | `linewidth` | Width of line. |
| `int` | `absmin` | Minimum value of variable. |
| `int` | `absmax` | Maximum value of variable. |
| `int` | `clean` | Clean or not. |

### Typical Syntax

The typical syntax of `k_plot` device is as follows:

```
k_plot name=uplot when=often 
  col0=col0 col1=col1 row0=row0 row1=row1 // defines the part of the graph window for this output
  color=WHITE*16+WHITE			  // colour of the "window" border	
  lines=1 				  // connect the dots
  clean=1                                 // clean window before drawing this graph
  ordmin=umin ordmax=umax		  // limits for the ordinate of the plot
  N=nx					  // the abscissa will be integer running from 1 to nx
  pgm={ord=u(abs,0,0,[u]);col=LIGHTRED};  // ordinate is value of u variable at the point, plot in light red
```

### Instances in Example Scripts

```
beatbox/data/scripts/sequential/FitzHughNagumo_model/fhn1.bbs
```

## k\_poincare

### Overview

Watches a "trajectory", represented by a set of k-assignments, for the
moment when a chosen component of this trajectory crosses the zero level.
You can choose to watch for crossing up, crossing down, or
crossing in either direction.

### Parameters

Since the `k_poincare` device works with a k-variable, it
does not apply to any subset of the dynamic grid, so must be run
with `nowhere=1`.

| Type | Name | Description |
| --- | --- | --- |
| `codeblock` | `pgm` | Semicolon-separated set of k-assignments, defining the trajectory. Semantics of the k-assignments are not the same and are not straightforward; the details are explained below under "Typical syntax". This is a mandatory parameter. |
| `int` | `which` | Identifies which trajectory component is watched for. Here `which=0` means it will be the component defined by the first assignment, `which=1` means it will be the second assignment, etc. Defaults to `0`. |
| `int` | `sign` | The sign of the crossings to be detected:  - `1` indicates positive crossings only (from negative to positive), - `-1` indicates negative crossings only (from positive to negative), - `0` indicates that crossings in either direction will be accepted.  Defaults to `1`. |

### Typical Syntax

The typical syntax of `k_poincare` device is as follows.

```
k_poincare nowhere=1 sign=1 pgm={front1=U1-umid;tfront1=t};
```

In this example, parameter `which` defaults to
`0`, so the first component of the trajectory, defined by
the k-assignment `front1=U1-umid`, is watched for. As a
result, the value of k-variable `front1` will be
`1` if the value of the expression of `U1-umid`
has changed from negative to positive since the last call of this device;
and it will `0` otherwise. This counter-intuitive semantics
applies only to the k-assignment identified by the `which`
parameter. For all other k-assigments, the semantics is more
expectable: the k-variable in the left-hand side takes the value of
the k-expression in the right-hand side, *if the crossing has
happened*; otherwise the value of the k-variable in the left-hand
side remains unchanged. More precisely, the value of
the k-expression in the right-hand side is estimated for the "time of
crossing", using linear interpolation between the previous and the
current calls of the device. In the given example, there is only one
"other" assignment, and the interpolated
value of the step counter `t` will be stored
`tfront1`.

### Instances of k\_poincare in Example Scripts

```
beatbox/data/scripts/sequential/FitzHughNagumo_model/fhn1.bbs
```

## load

### Overview

Loads data from a file saved by `dump` into the simulation
medium.

### Parameters

| Type | Name | Description |
| --- | --- | --- |
| `str` | file | Relative path to the file to be read. |
| `int` | rewind | Should the file be rewound before reading? |

### Typical Syntax

The typical syntax of `load` device is as follows. Assuming
that the dynamical layers are between `ode_layer0` (a
number) and the layer `ode_layer1` the syntax for the dump
device is, and that the dump file (see above) has been saved to a file
called `my_dump_file.dmp`, then the syntax is:

```
load when=begin v0=ode_layer0 v1=ode_layer1 append=0 file=my_dump_file.dmp;
```

### Instances of load in Example Scripts

All example scripts can be found in `beatbox/data/scripts` of the
package distribution. Examples of dump device can be found in the
following:

```
beatbox/data/scripts/sequential/FitzHughNagumo_model/fhn1_load.bbs
```

The purpose of this file is to give an example of the use of load
device. The input that this file expects is the dump
file `fhn1.dmp`. The output of this script is a dump file
at the end of the run. To run this script, use the following command:

```
Beatbox_SEQ fhn1_load.bbs
```

## poincare

### Overview

This device has a similar purpose to `k_poincare` device
described above, but is more efficient and less flexible. Rather than
watching for an arbitrary k-expression, it samples a dynamic variable
at a single point in the mesh, to detect a crossing in time of a
specified value. When the crossing has happened, the device just raises a
flag and remembers the value of the time step, linearly interpolated
to the moment of crossing, rather than that of any k-variable.

### Parameters

The `poincare` device must be run on a single point. Upper
space parameters `x1`, `y1`, `z1`
and `v1` are unused by this device. The `v0`
space parameter is the grid layer from which dynamic variable is to be sampled at point
`(x0,y0,z0)`.

| Type | Name | Description |
| --- | --- | --- |
| k-variable | `result` | Name of the k-variable into which the result should be stored: 1 if a crossing has been detected on this timestep, 0 otherwise. This must be a real variable. |
| k-variable | `timestep` | Name of the k-variable into which the timestep of the last crossing should be stored. An assignment will only be made to this variable on the timestep when a crossing is detected. This must be a real variable (even though it will be only assigned integer values). |
| `real` | `cross` | The crossing threshold. If previous and current values of the sampled dynamic variable straddle`cross`, a crossing is detected. |
| `int` | `sign` | The sign of the crossings to be detected:  - 1 indicates positive crossings only, - -1 indicates negative crossings only, - 0 indicates that crossings in either direction will be accepted. |

### Typical Syntax

The typical syntax of `poincare` device is as follows.

```
poincare x0=xout y0=yout z0=zout v0=[u] cross=umid sign=1 result=front timestep=tfront;
```

In this example, when the value of the `[u]` fild at point
syntax, front1 will become 1 when the variable `U1`
is increasing (slope sign 1). Additionally, the value of the integer
time counter `t` will be stored `tfront1`.

### Instances of k\_poincare in Example Scripts

```
beatbox/data/scripts/sequential/FitzHughNagumo_model/fhn1.bbs
```

## ppmout

### Overview

Produces PPM images of the space. Values from specified layers of the
simulation medium are mapped to colour channels by linear
interpolation using specified minima and maxima. ppmout produces a
new, sequentially numbered file every time it is run. In
three-dimensional meshes, ppmout produces non-standard PPM files, in
which multiple z-layers are stacked into a single file.

### Parameters

| Type | Name | Description |
| --- | --- | --- |
| `Sequence` | file | Format specifier for the sequence of files produced. |
| `int` | `r` | Layer of the simulation medium from which the red colour channel should be mapped. |
| `real` | `r0` | Value of the layer assigned to r that should map to 0. |
| `real` | `r1` | Value of the layer assigned to r that should map to 255. |
| `int` | `g` | Layer of the simulation medium from which the blue colour channel should be mapped. |
| `real` | `g0` | Value of the layer assigned to `g` that should map to 0. |
| `real` | `g1` | Value of the layer assigned to `g` that should map to 255. |
| `int` | `b` | Layer of the simulation medium from which the green colour channel should be mapped. |
| `real` | `b0` | Value of the layer assigned to b that should map to 0. |
| `real` | `b1` | Value of the layer assigned to `b` that should map to 255. |
| Geometry Only | | |
| `int` | `bgr` | Red value of the background colour, to be displayed at void points. |
| `int` | `bgg` | Green value of the background colour, to be displayed at void points. |
| `int` | `bgb` | Blue value of the background colour, to be displayed at void points. |

### Typical Syntax

The typical syntax of `ppmout` device is as follows.

```
ppmout file="ppm/%04d.ppm" mode="w" r=[u] r0=umin r1=umax g=[v] g0=vmin g1=vmax b=[i] b0=0 b1=255;
```

where the red, green, and blue channel are defined by maximum,
minumum, and mid-point values of user defined dynamical variables.

### Instances of ppmout in Example Scripts

All example scripts can be found in data/scripts of the package
distribution. Examples of dump device can be found in the following:

```
beatbox/data/scripts/sequential/fhn2.bbs
```

## record

### Overview

record writes values from the simulation medium to file in ASCII
format. A hierarchical set of separators
— `vsep`, `xsep`, `ysep`
and `zsep` — are used between
values. `recordsep` is used between records, i.e. separate
runs of the record device.

### Parameters

| Type | Name | Description |
| --- | --- | --- |
| `str` | `file` | Relative path to the file to be written. |
| `int` | `append` | Should the file be appended to? |
| `str` | `filehead` | Data to be placed at the top of the record file. |
| `str` | `vsep` | v-axis separator. Must be at most 2 characters in length. |
| `str` | `xsep` | x-axis separator. Must be at most 2 characters in length. |
| `str` | `ysep` | y-axis separator. Must be at most 2 characters in length. |
| `str` | `zsep` | z-axis separator. Must be at most 2 characters in length. |
| `str` | `recordsep` | Record separator. Must be at most 2 characters in length. |

### Typical Syntax

The typical syntax of `record` device is as follows.

```
record when=often file=output_record_file_name.rec append=0 x0=1 x1=xmax-1 y0=1 y1=ymax-1 z0=1 z1=zmax-1 v0=ode_layer0 v1=ode_layer1;
```

### Instances of record in Example Scripts

All example scripts can be found in data/scripts of the package distribution. Examples of
record device can be found in the following:

```
beatbox/data/scripts/sequential/fhn0.bbs
```

## reduce

### Overview

Reduce performs a reduction operation — sum, product, minimum or
maximum — over its space. The result of the operation is assigned to a
specified k-variable.

### Parameters

| Type | Name | Description |
| --- | --- | --- |
| `k-variable` | `result` | Name of the k-variable into which the result should be stored. |
| `str` | `operation` | Name of the operation to perform. Accepted values are “sum”, “product”, “min” and “max”. |
| `str` | `debug` | Name of the file to print debugging information to, possibly "stdout". |

### Typical Syntax

The typical syntax of `reduce` device is as follows.

```
reduce name=U_sample operation=max v0=[u] result=U;
```

### Instances of reduce in Example Scripts

All example scripts can be found in data/scripts of the package distribution. Examples of
record device can be found in the following:

```
beatbox/data/scripts/sequential/fhn0.bbs
beatbox/data/scripts/sequential/fhn1.bbs
```

## rk4

### Overview

Runge-Kutta 4th order; general pointwise ODE stepper for the rhs modules.

### Parameters

| Type | Name | Description |
| --- | --- | --- |
| `real` | `ht` | Timestep duration. |
| `str` | `ode` | Name of the RHS module to use. |
| `int` | `rest` | If nonzero, the device will make so many steps with the given model to find an approximation of the initial conditions, and then fill the whole device space with this initial condition. |
| `codeblock` | `par` | The parameters of the RHS module; their list depends on the module. The codeblock contains k-assignments of the form `<parameter>=<k-expression>`, separated by blank spaces. |

The list and meaning of parameters are exactly the same as for the
`euler` device; we repeat it here for
user's convenience.

The parameter `ht` defines a duration of one time step used
by the solver.

The parameter `ode` is a name of the rhs module that implements the
cellular kinetics. This module is used for the simulation. The `ode`
model has to follow the rhs format.

The parameter `rest` is used if the device determines the initial
conditions of the dynamical variables by a steady state
approximation. The device runs the simulation for the number of steps
specified in `rest` using the initial conditions as specified in the
code of `rhs` module.

Model dependent parameters of rhs module are passed to rk4 device
through the `par` parameter in `BBScript`. The assignment has to
follow the format `{<parameter>=<value>}` (within curly brackets `{}`
as shown). When multiple parameters are assigned, their definition
must be all given within the same curly brackets and each assignment
must be separated by a blank space (such as space, or newline).

### Typical Syntax

The typical syntax of `rk4` device is precisely the same as
that of `euler` device. For instance,

```
rk4 when=run v0=[u] v1=[v] ht=ht ode=cgle par={alp=0 Omg=-1 bet=0};
```

(this is from the example quoted below).

### Instances of rk4 in Example Scripts

One example is provided in
`beatbox/data/scripts/sequential/RK4` of the package
distribution. This example solves an initial-value problem for a
system of two coupled nonlinear ODEs, with a known exact solutions,
and prints out the norm of the difference between the numerical
solution and the exact answer at the final moment of time. The example
is complete with a `Makefile` which allows to compute a
series of runs and produce the graph of the numerical convergence,
illustrating the fourth-order accuracy of the solver.

## rushlarsen

### Overview

The rushlarsen device is an integration device for ionic models which
allows to exploit Rush-Larsen exponential integration scheme for the
gate variables and Matrix Rush-Larsen scheme for the Markov
chains. The device tabulates the transition rates that depend on the
voltage (and possibly on unchangeable physical constants) before the
simulation time for the reference at the run time in order to speed-up
the simulations.

Ionic models have to be defined in a different way from a generic RHS
model description, as this definition has to make distinction between
gate variables and non-gate variables.

They do implement the formulas for the time derivative of all
dynamical variables only for "other" variables. The equations for
gating and Markov chain are implied from their standard from a
standard form and transition rates definition. The details about the
definition of ionic modules is found in *specification of ionic modules.*

### Parameters

Table 1: Parameters to set up rushlarsen device in `BBScript`

| type | name | description |
| --- | --- | --- |
| `real` | `ht` | time step duration. |
| `str` | `ionic` | name of the cellular model in ionic format. |
| `int` | `rest` | number of steps to approximate initial conditions of the dynamical variables (resting state). When the value is set to zero (`0`), the default initial conditions from the module are used. |
| `codeblock` | `par` | model-dependent parameters of ionic module. The parameter is set by a codeblock in a form `par={<parameter>=<value>}` separated by blank spaces if more than one parameter is specified. |
| `str` | `order` | the order of execution of substeps: one of `tog`, `tgo`, `totg`, where the substeps are done consecutively: `t` stands for table look-up, `o` stands for "other" variables and computation non-tabulated gates, `g` stands for tabulated gating variables. |
| `str` | `exp_ngate` | defines method of computation of non-tabulated gates: non-zero for exponential (Rush-Larsen); zero (`0` default) for forward Euler method. |
| `real` | `Vmin, Vmax` | minimal and maximal value of voltage for tabulation. Default is `-200`, `200` respectivelly. |
| `real` | `dV` | voltage-step for the tabulation of gating transition rates. Default is `0.01`. Value `0.0` disables tabulation. |
| `str` | `exp_mc` | specifies method of integration of Markov chains: `mcfe` forward Euler, `tabmrl` MRL with tabulation, `ntabmrl` MRL without tabulation (MRL only if subchain has a control variable, otherwise uses forward Euler) |

The parameter `ht` defines the duration of one time step used in
forward Euler and Rush-Larsen calculations.

The model of cellular kinetics is passed to rushlarsen device through
the string parameter `ionic`. The module has to follow the ionic
format.

Module dependent coefficients in the model equations of the ionic
model can be set using the `BBScript` parameter `par`. This assignment
is done through a block of code in a form `<parameter>=<value>`. The
`<parameter>` is a name of the coefficient specific to the on
particular ionic module (specified in `ionic`). If more than one
"parameter" is specified all specification must be within the same
curly brackets and each assignment must be separated by a blank space.

The rushlarsen device can the approximate resting state of the
variables to be used as initial conditions. This is done by simulation
for the number of steps specified in `rest` parameter.

The computation of the different groups of dynamical variables
(i.e. tabulated and non-tabulated gates, and "other" variables), is
done consecutively. The particular order in which the computations are
performed can be controlled by the `order` parameter. The `order` can
be set to the value of one of the following codes: `tog`, `tgo`, or
`totg`. The order of letters in the codes corresponds to the order in
which the particular group is computed. The letter `t` stands for
finding the reference to a particular entry in a look up table of the
tabulated voltage-dependent gates. The letter `g` stands for computing
of the tabulated gates. The letter `o` stands for computing of the
remaining variables i.e. non-gating variables followed by computing of
non-tabulated gating variables and Markov chain variables.

For example, when the code `totg` is specifies the computation will be
done in the following order:

1. tabulated gates, followed by the computation of
2. non-gating, non-tabulated and Markov chain variables, and then
3. tabulated gates again (now for updated values of dynamical
   variables), and finally the computation of
4. tabulated gating variables.

This is repeated for each time step during the whole simulation.

The parameter `exp_ngate` is used to choose the integration algorithm
of the non-tabulated gating variables. The value `0` (default) stands
for computation by forward Euler. If the value is non-zero, the
non-tabulated gates are computed using Rush-Larsen technique. In
cases, when the non-tabulated gates are not the source of numerical
instability the forward Euler method is faster, because it avoids
computationally expensive calculation of exponentials.

Three parameters are used to define the tabulation of the transition
rates of the voltage-dependent gate variables. Namely those parameters
set up the lower and upper limits of the tabulation by `Vmin` (default
`-200`) and `Vmax` (default `200`) respectively, and the step in the
voltage grid by `dV` (default `0.01`).

If the value of `dV=0.0`, the tabulation is be disabled and all
transition rates are computed on-the-fly. If the value of membrane
voltage exceeds the limits of tabulation, the transition rates are
found on-the-fly, until the value of voltage is restored to within the
given limits of the table.

Parameters for the integration of Markov chains `exp_mc` can be
provided in a `BBScript`. The possible values are `mcfe` for forward
Euler, `tabmrl` for MRL with tabulation, and `ntabmrl` for MRL without
tabulation. However, the method which can be used used on a particular
subchain depends on other characteristics of the subchain such as a
dependency of multiple variables.

### Typical Syntax

The typical syntax of rushlarsen device is as follows. Assuming that
the ionic model occupies layers from between `ionic_layer0` to
`ionic_layer1`, the code of the ionic model that you are using od
ionic\_model_ and the user-defined (different from the default) model
parameters with names `par1,par2` are contained in k-variables `p1,p2`,
the syntax is:

```
rushlarsen name=name_to_device_call v0=ionic_layer0 v1=ionic_layer1 ht=ht ionic=ionic_model exp_mc=tabmrl par={par1=p1; par2=p2;};
```

### Instances of `rushlarsen` in Example Scripts

All example scripts can be found in `beatbox/data/scripts` of the
package distribution. Examples of scripts for simulation of ionic
modules with rushlarsen device can be found in the following:

```
beatbox/data/scripts/sequential/BR/tog.bbs
beatbox/data/scripts/sequential/BR/tgo.bbs
beatbox/data/scripts/sequential/BR/otg.bbs
beatbox/data/scripts/sequential/CRN/compare1D.bbs
```

for models without Markov chains.

See more detailed discussion of these scripts below in the sections
dedicated to the `br77` and `crn98` ionic models.

A script for simulation with Markov chain can be found in:

```
beatbox/data/scripts/hh52/hh52m.bbs
```

## sample

### Overview

Assigns a dynamic variable value from a specified point in the mesh to
a k variable.

### Parameters

The sample device must be run on a single point. Upper space
parameters, `x1`, `y1`, `z1`
and `v1` are unused by this device. The `v0`
space parameter is the dynamic variable to be sampled at
point `(x0, y0, z0)`.

| Type | Name | Description |
| --- | --- | --- |
| `k` variable | result | Name of the k variable into which the sampled value should be stored. |

### Typical Syntax

The typical syntax of `sample` device is as follows.

```
sample x0=x_loc y0=y_loc z0=z_loc v0=ode_layer result=name_of_place_holder;
```

### Instances of sample in Example Scripts

All example scripts can be found in data/scripts of the package distribution. Examples of
record device can be found in the following:

```
beatbox/data/scripts/sequential/fhn0.bbs
beatbox/data/scripts/sequential/fhn1.bbs
```

## singz

### Overview

Detects, plots, remembers in k-variables and/or outputs to file the "singular points",
defined as intersections of isolines of two selected dynamic
fields. Mostly will be used to detect spiral wave tips. It also
computes the "tip orientation", in two ways: as the azymuthal angle of
the gradient of the first field (angle phi, in radians), and the same
for the second field (angle psi), and the cosine of the angle between
the two gradients. Device `singz` is essentially
two-dimensional; when called in a 3D simulation, it simply does the
same job in every *z*=const cross-section, hence the 'z' in the
name of the device. At present, true three-dimensional filaments can
be obtained by post-processing, say by
`ezview`.

The results of the device are available in three forms:
shown graphically,
written in an output file
and remembered in k-variables.
The graphical window for the device is defined in
the usual way; the tips are represented by pixel-size dots, and the
tips from the most recent call are shown in a different colour; this
is controlled through standard device parameters (details to
follow). The file output is defined by specific device parameters, as
described below. Since there are potentially more than one
`z`-section involved, and each section may contain more
than one singular point, k-variables store statistics of the found
points: their total number, centroid and standard deviations of the
coordinates.

### Parameters

| Type | Name | Default | Description |
| --- | --- | --- | --- |
| `int` | `v0` |  | The layer in which the first dynamic field is located. |
| `int` | `v1` |  | The layer in which the second dynamic field is located. |
| `real` | `c0` |  | The constant defining the first dynamic field's isoline. |
| `real` | `c1` |  | The constant defining the second dynamic field's isoline. |
| `str` | `file` |  | Relative path to the output file that will contain the coordinates of the found singular points. |
| `int` | `append` | 0 | Should the file be appended to? |
| `str` | `pointsep` | " " | In the file output, separator between records of individual points |
| `str` | `sectionsep` | `"\t"` | In the file output, separator between outputs from different z-sections, if any |
| `str` | `recordsepsep` | `"\n"` | In the file output, separator between outputs from different calls of the device, done at different times. |
| `int` | `everypoint` | `0` | In the file output, time `t` value will precede coordinates of every found singularity point. |
| `int` | `everysection` | `0` | In the file output, time `t` value will precede the output from every z-section. |
| `int` | `everyrecord` | `1` | In the file output, time `t` value will precede the output from every call of the device.   **NB** Exactly one of `everypoint,everysection,everyrecord` must be 1, the others must be 0. |
| `int` | `precise_orientation` | 0 | If nonzero, the orientation of the tips is calculated using a higher-order scheme, involving a 4x4 template.   Not available in MPI because the thickness of the inter-domain "halo" slices is restricted to one grid point. |
| `k-variable` | `Npoints` |  | Name of the k-variable for the number of singular points. |
| `k-variable` | `xmean` |  | Name of the k-variable for the mean `x`-coordinates of the singular points. |
| `k-variable` | `ymean` |  | Name of the k-variable for the mean `y`-coordinates of the singular points. |
| `k-variable` | `zmean` |  | Name of the k-variable for the mean `z`-coordinates of the singular points. |
| `k-variable` | `xstddev` |  | Name of the k-variable for the standard deviation of the `x`-coordinates of the singular points. |
| `k-variable` | `ystddev` |  | Name of the k-variable for the standard deviation of the `y`-coordinates of the singular points. |
| `k-variable` | `zstddev` |  | Name of the k-variable for the standard deviation of the `z`-coordinates of the singular points. |

All k-variables must be of real type, and are optional
parameters. If a k-variable is not specified, the corresponding
quantity is not stored anywhere. The mean and standard deviation are
assigned only if nonzero number of points are detected.

### Typical Syntax

We give three examples, two from two-dimensional simulations and one
from a three-dimensional simulation.

As in
`beatbox/data/scripts/sequential/FitzHughNagumo_model/fhn2.bbs`:

```
// Detect the tip of the spiral
singz when=out
  v0=[u] c0=0         // trace intersection of
  v1=[v] c1=0         // u(x,y,t)=0 and v(x,y,t)=0
  col0=col2 col1=col3 // plot it 
  row0=row0 row1=row1 // in the right panel
  color=WHITE*16+LIGHTRED // red trace with white head
  file=[0].trj        // and write into file fhn2.trj
;
```

This will, at all time steps when the k-variable `out` is
nonzero, find intersections of the lines *u(x,y)=0* and
*v(x,y)=0*. The results will be shown in the area of the
graphic window between columns `col2` and
`colr3` and between rows `row2` and
`row3`. The tips will be shown by pixel-size dots, light-red for
"previous" points and white for the most recent points. The results
will be also output into a disk file, eponymous with the script except the
extension `.trj`. This example uses default values of the
output customization parameters, which will generate output like

```
         0  50.757942 50.763065 0.891785 -0.556892 0.121816 	
       100  51.842682 52.343216 0.983348 -0.491837 0.095465 	
       200  51.964546 53.362553 1.206323 -0.253890 0.110358 	
       300  51.616615 54.403961 1.372360 0.091922 0.286295 	
       400  50.858425 55.406269 1.458319 0.323225 0.422047 	
 ...
```

where
the first column contains values of the time step counter
`t`;
the secondcolumn contains `x`-coordinates of the
singular points,
the third column contains `y`-coordinates of the
singular points,
the fourth and fifth columns contain the polar angles of the gradient
vectors of the first field `v0=[u]` and of the second field
`v1=[v]` used for the tip detection, estimated at the tip,
and the sixth column contains the cosine of the angle between the two
gradients.

As in
`beatbox/data/scripts/sequential/FitzHughNagumo_model/singz.bbs`:

```
// Detect the tip of the spiral
def real ntip; def real xtip; def real ytip; // k-variables to record the tip
singz when=out
  v0=[u] c0=0         // trace intersection of
  v1=[v] c1=0         // u(x,y,t)=0 and v(x,y,t)=0
  Npoints=ntip 	      // number of tips detected
  xmean=xtip          // ... their mean x coord 
  ymean=ytip          // ... their mean y coord
;
```

This will work as in the previous example, except the found tips are
neither drawn on screen nor recorded into the file, but are just
remembered in k-variables `xmean` and
`ymean`. These variables can be used in other devices. In
this particular example, these are used by `k_draw` to
display on screen as a curve (rather than by dots), and by
`k_print` to output to a file.
  
NB: `ntip` is a real variable, even though it is
intended to contain only integer values. Thanks to this, this variable
can be used as a control variable by other devices, which are intended
to work if and only if a nonzero number of tips are recorded.

As in
`beatbox/data/scripts/parallel/Barkley/scroll.bbs`:

```
singz when=often 
          file=[outdir]/singz.dat append=0 
          v0=[u] c0=umid 
          v1=[v] c1=vmid
          everypoint=1 
          everysection=0 
          everyrecord=0
          pointsep="\n" 
          sectionsep="" 
          recordsep="\n"
;
```

This is a 3D simulation and parameters `every...` and
`...sep` are chosen non-default. This device will only do
file output, which looks like this:

```
         0 40.917686 40.583931 1.000000 0.724878 1.409299 0.774785
         0 40.899208 40.603081 2.000000 0.705210 0.175109 0.862756
         0 40.879932 40.623329 3.000000 0.332309 0.257325 0.997190
         0 40.859558 40.645096 4.000000 2.811086 0.256183 -0.832778
         0 40.837597 40.669064 5.000000 0.618518 0.096927 0.867028
...


        14 40.991848 42.630165 15.000000 3.056580 -3.002670 0.975031
        14 40.968472 42.656673 16.000000 0.429959 -0.182781 0.818076
        14 40.943298 42.676113 17.000000 2.346203 -3.094432 0.665562
        14 40.914688 42.689327 18.000000 0.760891 -0.161832 0.603651
        14 40.878613 42.699989 19.000000 1.629990 -1.939269 -0.909936
...


        28 40.983856 43.442799 21.000000 1.105454 -2.485222 -0.900845
        28 40.935574 43.450146 22.000000 1.148618 -0.336345 0.085727
        28 40.887096 43.457321 23.000000 0.525845 -0.246125 0.716538
        28 40.834824 43.464394 24.000000 3.000749 -0.231454 -0.995898
        28 40.780441 43.471146 25.000000 0.581991 -0.102920 0.774476
...
```

There is now more columns than in the 2D case: the added is the fourth
column, which contains the `z`-coordinates of the tip
points. The lines corresponding to different values of time counter
are separated by extra empty lines; however, if this is run in
parallel, there may be extra empty lines, which are to be eliminated
by postprocessing.

This may be considered as a "feature" that hopefully will be fixed a some stage.

### Instances of sample in Example Scripts

Examples of
`singz` device can be found in the following:

```
beatbox/data/scripts/BeatboxWorkshop/CRN_model/pd_crn2.bbs
beatbox/data/scripts/BeatboxWorkshop/FitzHughNagumo_model/fhn2.bbs
beatbox/data/scripts/parallel/Barkley/scroll.bbs
beatbox/data/scripts/parallel_Hector/phasedbn_parallel/pd_crn2.bbs
beatbox/data/scripts/sequential/Barkley_model/implicit.bbs
beatbox/data/scripts/sequential/CRN_model/pd_crn2.bbs
beatbox/data/scripts/sequential/FitzHughNagumo_model/fhn2.bbs
beatbox/data/scripts/sequential/FitzHughNagumo_model/fhn_crossFieldStim_ffr_slice.bbs
beatbox/data/scripts/sequential/FitzHughNagumo_model/fhn_spiral_ffr_slice.bbs
beatbox/data/scripts/sequential/FitzHughNagumo_model/fhn_spiral_ffr_slice_aniso.bbs
beatbox/data/tests/fhn2.bbs
beatbox/data/tests/fhn3.bbs
beatbox/data/tests/fhn3side.bbs
```

## stop

### Overview

Stops the simulation run.

### Parameters

None.

### Typical Syntax

The typical syntax of `stop` device is as follows. Assuming
that the variable for end has been defined in the variable `end`, the syntax is

```
stop when=end;
```

### Instances of stop in Example Scripts

All bbs scripts must have a call to the stop device. The stop devices occurs
in all the example bbs scripts.

## update

`update` updates the run time visualisation.

### Parameters

| Type | Name | Description |
| --- | --- | --- |
| `int` | `when` | Frequency of updating |

### Typical Syntax

```
update when=often; // run time graphics are updated when often is true.
```

### Instances in Example Scripts

```
beatbox/data/sequential/pd/scripts_crn0.bbs
beatbox/data/sequential/pd/scripts_crn1.bbs
```

## vtkout2

### Overview

Outputs legacy VTK file for 2D model. The header is in ASCII, and the data
are in double binary format.

### Parameters

| Type | Name | Description |
| --- | --- | --- |
| `int` | `r` | Dynamical layer to output |
| `int` | `r0, r1` | minimum and maximum of the expected values in later r |

### Typical Syntax

```
/* Note: the g and b layers are there to allow vtkout2 syntax to be similar to the ppmout syntax */
vtkout2 when=often file="vtk/fk2%06d.bin.vtk" mode="w" r=[u] r0=umin r1=umax g=[v] g0=vmin g1=vmax b=[i] b0=0 b1=255;
```

### Instances in Example Scripts

data/scripts/sequential/FentonKarma\_model/fk2\_san.bbs

# Beatbox Cell Model Reference

## Generic ODE definitions

### Fitz-Hugh Nagumo Cell Model

This model was published in:

> "Varieties of spiral wave behavior: An
> experimentalist’s approach to the theory of excitable media", by
> Arthur Winfree, *Chaos* **1(3):** pp. 303-334, 1991,

and simulates a generic action potential. It consists of 2
variables. The voltage is in layer 0. The name of the module
is `fhncub` and `fhncubpar`. This implementation
is based on formulas given in the Chaos paper. The time integration is
using explicit Euler for both variables. The example of FHN cell
model AP is in:

```
beatbox/data/scripts/sequential/FitzHughNagumo_model/fhn0_ap.bbs
```

This bbs script has no inputs, and its output is a record file called
`fhn_ap.vtg`. To run, use the command:

```
Beatbox_SEQ fhn0_ap.bbs
```

After running this bbs script, you can produce an AP profile as shown
in the figure below.

### Fenton Karma Cell Model

The Fenton Karma model (FK) consists of 3 variables. The voltage is in
layer 0. The name of the module is `fk`. This model was
published in:

> "Vortex dynamics in three-dimensional continuous myocardium with fiber
> rotation: Filament instability and fibrillation", by F Fenton and A
> Karma, *Chaos*, **8(1):** pp. 20-47, 1998,

and simulates a generic action potential. This implementation is based
on formulas given in the paper. The time integration is using explicit
Euler for both variables. The example of FK cell model AP is in:

```
beatbox/data/scripts/sequential/FentonKarma_model/fk0_ap.bbs
```

This bbs script has no inputs, and its output is a record file called
fk0\_ap.vtg. To run, use the command:

```
Beatbox_SEQ fk0_ap.bbs
```

After running this bbs script, you can produce an AP profile as shown
in the figure below.

### Fenton Karma Modified Cell Model

The FK model (previous subsection) was modified during the Beatbox
project (2011-2013) and developed to produce mouse sinoatrial node and
atrial action potentials. It has been accepted for publication as
a *IEEE Proceedings (EMBC 2013)*. The name of the module is
called `fkmod`. The examples of the cell model are in:

```
beatbox/data/scripts/sequential/FentonKarma_model/fk0_atrial.bbs
beatbox/data/scripts/sequential/FentonKarma_model/fk0_san.bbs
```

where a `Makefile` detailing the SAN and atrial command
line inputs is give. To run the atrial cell, type:

```
make atrial
```

and to run the SAN cell, type:

```
make san
```

We get this figure for SAN AP:

and atrial AP profile as shown below.

### LRD

This model was published in:

> "A dynamic model of the cardiac ventricular action potential", by CH
> Luo and Y Rudy, *Circ. Res.*, **74(6):** pp. 1071-1096, 1994,

and simulates the human ventricular action potential. It consists of
24 variables. The voltage is in layer 0. The name of the module
is `lrd`. This code is based on Darren Sandham's code,
University of Liverpool, 2005. The time integration of gating
variables is using Rush-Larsen method, and all other variables is
using explicit Euler. The example of LRd cell model AP is in:

```
beatbox/data/scripts/sequential/LRD_model/lrd0_ap.bbs
```

This script requires no input and outputs a vtg file with the AP
data. It can be run using:

```
Beatbox_SEQ lrd0_ap.bbs
```

You can produce an AP profile as shown in the figure below.

### CRN

This model was published in:

> "Ionic mechanisms underlying human atrial action potential properties:
> insights from a mathematical model", by Courtemanche M, Ramirez RJ,
> Nattel S*, Am J Physiol Heart Circ Physiol*
> **275:H301-H321.**: pp. H301-H321, 1998,

and simulates the human atrial action potential. It consists of 21
variables. The voltage is in layer 0. The name of the module
is `crn`. This implementation is based on code received
from the author. It does not provide the true ODE right-hand sides,
instead it performs the timestep, using Rush-Larsen formula for all
gating variables and explicit Euler for all other variables. Hence it
requires the time step as parameter `ht`. The example of
CRN cell model AP is in:

```
beatbox/data/scripts/sequential/CRN_model/crn0_ap.bbs
```

This script requires no input and outputs a vtg file with the AP
data. It can be run using:

```
Beatbox_SEQ crn0_ap.bbs
```

You can produce an AP profile as shown in the figure below.

### TTNNP04 Human Ventricle Model from author source code

This model was published in:

> "A model for human ventricular tissue",
> by K. H. W. J. ten Tusscher, D. Noble, P. J. Noble and A. V. Panfilov,
> *Am J Physiol Heart Circ Physiol*
> **286**: pp. H1573-H1589, 2004,

and simulates the human ventricular action potential. It consists of
17 variables. The voltage is in layer 0. The name of the module
is `ttnnp04`. This implementation is based on
author's code retrieved 2013/02/02. It does not provide the true
ODE right-hand sides, instead it performs the timestep, using
Rush-Larsen formula for all gating variables and explicit Euler for
all other variables. Hence it requires the time step as
parameter `ht`.

```
beatbox/data/scripts/sequential/TenTusscherPanfilov_modelS/ttnnp04_ap.bbs
```

Beatbox program to demonstrate the ten Tusscher et al. 2004 human
ventricle AP. There are no inputs,and the output is ttnnp04\_ap.vtg.
To run,

```
Beatbox_SEQ ttnnp04_ap.bbs
```

You can produce an AP profile as shown in the figure below.

### TTP Human Ventricle Model from CELLML source

This model was published in:

> "Alternans and spiral breakup in a human ventricular tissue model", by
> K. H. W. J. ten Tusscher and A. V. Panfilov,
> *J Physiol Heart Circ Physiol* **291**: pp. H1088–H1100, 2006,

and simulates the human ventricular action potential. It consists of
19 variables. The voltage is in layer 0. The name of the module
is `ttnp`. This implementation is based on CELLML version
and provides true ODE right-hand sides, unlike TTP06 implementation
described below. Note that the resulting ODEs are very stiff and
require very small time steps. The example of TTNP cell model AP is
in

```
beatbox/data/scripts/sequential/TenTusscherPanfilov_modelS/ttp06_ap.bbs
```

Beatbox program to demonstrate the ten Tusscher et al. 2006 human
ventricle AP. There are no inputs,and the output is ttp06\_ap.vtg. To
run,

```
Beatbox_SEQ ttp06_ap.bbs
```

You can produce an AP profile as shown in the figure below.

### TTP06 from author source code

This model was published in:

> "Alternans and spiral breakup in a human ventricular tissue model",
> by K. H. W. J. ten Tusscher and A. V. Panfilov,
> *J Physiol Heart Circ Physiol* **291**: pp. H1088 –H1100, 2006,

and simulates the human ventricular action potential. It consists of
19 variables. The voltage is in layer 0. The name of the module
is `ttp06`. This implementation is based on
author's code  retrieved 2013/02/08. Unlike implementation TTNP
described above, this one does not provide the true ODE right-hand
sides, instead it performs the timestep, using Rush-Larsen formula for
all gating variables and explicit Euler for all other variables.
Hence it requires the time step as parameter `ht`. The
following script simulates the AP:

```
beatbox/data/scripts/sequential/TenTusscherPanfilov_modelS/ttp06.bbs
```

A slightly more advanced example of this model can be found in the directory:

```
beatbox/data/scripts/sequential/TenTusscherPanfilov_modelS/ttp06/
```

The `benchmark.bbs` script there does the computational
job, `Makefile` describes the workflow leading to the final
report and `report.pdf` is the report itself.

### cgle

This RHS module is not a cellular model as such, but can be used to
run simulations of the important theoretical reaction-diffusion
system, known as Complex Ginzburg-Landau Equation, assumed in the
complex-valued form

∂*w*/∂*t*
 = 
(1+*i*Ω) *w*
+
(-1+*i*α) *w*|*w*|^2^
+
(1+*i*β) ∇^2^*w*

(different but equivalent formulations can be found in literature).
The RHS module consists of 2 variables, layer 0 containing the
real part *u* and layer 1 containing the imaginary part *v* of the complex
field *w*. Apart from `Omg` for Ω,
`alp` for α and `bet` for β, this
module accepts parameters `Iu` and `Iv`, which
are expected to contain the Laplacians of the fields *u* and
*v* respectively, perhaps computed by the `diff`
device. This RHS module is used in one example in the distribution,
in `beatbox/data/scripts/sequential/RK4`. See the
description of the `rk4` device for detail
of this example.

## Ionic model definitions

### BR77

This model was published in:

> "Reconstruction of the action potential of ventricular myocardial
> fibres",
> by G. W. Beeler and H. Reuter,
> *J Physiol* **268**: pp. 177-210, 1977,

and is an early model of the mammalian ventricular action
potential. It consists of two non-gating variables variables (`V,
ca`) and six gating variables (`x1,m,h,j,d,f`). The
tabulated functions are the 12 transition rates for the six gates,
plus the voltage-dependent functions `ik1` and
`gix1`. The voltage is in layer 0. The name of the ionic model
is `br77`. This implementation is based on the paper.
following scripts simulates the AP:

```
beatbox/data/scripts/sequential/BR/tgo.bbs
beatbox/data/scripts/sequential/BR/tgo.bbs
beatbox/data/scripts/sequential/BR/tog.bbs
```

for different orders of execution of the substeps in the Rush-Larsen
scheme. These scripts simply calculate the action potentials for the
initial condition which is the voltage value of 50 mV above the
resting state and all other variables above the resting state. They
take two command-line parameters, one is the value of the time step,
and the other the name of the output file for the action
potential. The action potential is output in two columns, first for
the time, in milliseconds, and the second for the voltage in
millivolts.

### CRN98

This model was published in:

> "Ionic mechanisms underlying human atrial action potential properties:
> insights from a mathematical model", by Courtemanche M, Ramirez RJ,
> Nattel S*, Am J Physiol Heart Circ Physiol*
> **275:H301-H321.**: pp. H301-H321, 1998.

This is the same model as implemented in the "generic" RHS module
`crn` with the following differences:

- Three of the "dynamic variables" of the `CRN` module are in
  fact not dynamic variables, i.e. there are no differential equations
  for them, but they are explicit functions of the true dynamic
  variables, so they have been removed from the list of dynamic
  variables, thus shortening the length of the state vector from 24 to
  21. The removed variables are `cmdn,trpn,csqn`, they
  describe the states of the Ca buffers and are taken in the
  quasi-stationary approximation.- As required by the "ionic cell" format, the description of
    dynamics of gating variables and non-gating variables are separated,
    which allows the `rushlarsen` device to update gates by
    Rush-Larsen scheme. In that case the choice `order=tog`
    gives the the closest results to the `CRN` module, which
    corresponds to the Courtemanche's author code; exact correspondence
    is not possible as Courtmanche's code intermingles updates of
    different classes of variables. The best accuracy is however
    achieved at `order=totg`.

The voltage is in layer 0. The name of the ionic model
is `crn98`. This implementation is based on the
Courtemanch's author code, with necessary transformations to bring it
to the require format. An example of using this model can be found in

```
beatbox/data/scripts/sequential/CRN/compare1D.bbs
```

which calculates simultaneous propagation of pulses in the 1D cable in
the two implementations of the Courtemanche et al. 1998 model, one
with `euler` device and `crn` model, and the
other with `rushlarsen` device and `crn98`
model. The script does not take any command-line parameters and does
no output to disk files apart from the log file. The state vector for
the `crn` model is allocated in layers from 0 to 23, and
for the `crn98` model in layers from 24 to 44, with layer
45 used as auxiliar layer for calculating the diffusion. Unlike many
other examples, this script uses `diffstep` instead of
`diff`, so it can use the same layer 45 for both diffusion
operators, and does not require passing the value of the diffusion
terms to the kinetic timesteppers `euler` and
`rushlarsen` via the usual layer-substitution trick such as
`IV=@[aux]`. Both solutions are plotted on screen so the
user can visually compare the correspondence between the two
implementations. At the chosen parameters, the two curves
would almost completely overlap hence they are plotted with a shift of
one pixel horizontally (notice the `col0` and
`col1` parameters of the two `k_plot`
devices. The user is invited to experiment with different
`order` choices and discretization steps.

### TTP2006

This model is a reformulation of the authors code
*`ttp06`* published as:

> "Alternans and spiral breakup in a human ventricular tissue model", by
> K. H. W. J. ten Tusscher and A. V. Panfilov, *J Physiol Heart Circ
> Physiol* **291**: pp. H1088 –H1100, 2006,

The name of the module is `ttp2006`.

The total calcium concentration in each compartment (Sarcoplasmic
Reticulum, sub-space, and bulk intracellular space) are now given as
differential equations. The dissolved calcium concentration is then
obtained using a formula for quasi-stationary approximation.

This module is written in ionic format. This includes the following
differences with respect to `ttp2006`:

- the variables were divided to: tabulated gates (dependent only on
  voltage), non-tabulated gates (dependent on other variables (here on
  calcium concentrations), and other variables (voltage, and ionic
  concentrations; as required by the rushlarsen device.
- The RR gate of the RyR receptor on sarcoplasmic reticulum is now
  calculated using rushlarsen formula.

### HH52

The model converts the Hodgking-Huxley (1952) giant squid axon model
into ionic format. The implmentation is based on the RHS format in
`hh`. All the gates are tabulated and can be computed using
Rush-Larsen method.

### HH52M

Experimental module for testing and developement of matrix Rush-Larsen
method into rushlarsen device. The model is based on the
*`hh52`* model. The INa and IK current is substituted by equivalent Markov
chain definition.

An example of a script using rushlarsen module with `hh52m` cellular
model is in:

```
beatbox/data/scripts/sequential/hh52/hh52m.bbs
```


---

Copyright © (2010-2013) Vadim Biktashev, Alexander
Karpov,Irina Biktasheva, Ross McFarlane;  
Copyright © (2012-2013) Sanjay Kharche  
This file is part of the Beatbox distribution
