# Supplementary material for: BeatBox—HPC simulation environment for biophysically and anatomically realistic cardiac electrophysiology
Source: PLoS One. 2017 May 3;12(5):e0172292. doi: 10.1371/journal.pone.0172292 (PMC5415003; doi:10.1371/journal.pone.0172292)
Supplement: S1 Code — Zip-file S1_code.zip containing distribution of the BeatBox, version beatbox-public-v1.7.982, including source code, configuration and makefiles, documentation, sample scripts etc. (ZIP) [file pone.0172292.s001.zip › beatbox-public-v1.7.982.zip!/beatbox-heart-beatbox-public-05c855e/doc/beatbox_quickstart.html]

Beatbox Quick Start Guide


# Beatbox Quick Start Guide

---


## Table of Contents

---


# Beatbox, briefly

Beatbox is a unified cardiac simulation environment. It provides:

- A script interpreter to eliminate manipulation of low level code,
  and avoid lengthy recompilations each time a new simulation is
  constructed;
- Serial and parallel (MPI) simulations;
- Robust ODE solvers (Euler, RK4);
- Robust finite differences PDE solvers for isotropic and anisotropic
  tissue models on regular and irregular boundary domains;
- Major CellML cardiac models in the repository;
- Multiscale tissue modelling: 0D individual cell simulation, 1D fiber,
  2D sheet and 3D slab of tissue, up to anatomically realistic whole
  heart simulations using the provided tissue model repository.
- Run time measurements including tip tracing, filament tracing, ECG,
  samples of any variables;
- Extensibility: You can easily plug in your own solvers as well as cell
  kinetics models and tissue models (e.g. MRI finite difference meshes)
  into Beatbox's highly structured code.

# Who should read this guide

This quick-start guide is for users who wish to start using Beatbox in
their cardiac simulation project. This quick-start provides an easy to
read and follow document using which the user can start using Beatbox
immediately.

A full manual is
available **HERE**.

A full manual for making BBG files is
available **HERE**.

# Pointers to useful tutorials

If you are new to Unix like environments, electrophysiology, cardiology, or
anything to do with cardiac (or electrophysiology) simulations, then read the
following tutorials.

- For a tutorial on Linux, you can see the following:
  Linux Tutorial

# Prerequisites

Beatbox is a Unix-based piece of software. Thus, it will also work on
Mac (tested on Leopard and Snow Leopard OS X) and standard Linux:
tested on Fedora and Red Hat Linux, though any other Linux
distribution should work. The preferred working shell for Beatbox is
the `bash` shell. This choice of shell will assist in
easily executing the examples described below. For larger
non-interactive simulations, you may wish to choose `tcsh`
or `bash`.

Beatbox requires `gcc` (sequential) and `mpicc`
(MPI parallel) C compilers. Beatbox also requires standard math and
X11 libraries.

To obtain the Beatbox source code off the project repository, you need
a client program for Subversion (SVN). You can also obtain a copy of
the distribution source code from the authors.

# Obtaining Beatbox Source Code and Examples

These are a standard installation procedures assuming your
computer satisfies requirements stated previously. These instruction
assume that you are using `bash` or a similar command-line
shell.

## Releases

To obtain a BeatBox release, download the most recent tarball from
the repository, which at the moment of writing this is at
`http://empslocal.ex.ac.uk/people/staff/vnb262/software/BeatBox/`
, and unpack it. Chose the place at your convenience. It could be
your home directory or any other directory in your space.
Say, if you downloaded the file
`beatbox-1.1-417.tar.gz` into your
`$HOME` directory, then you can do

```
cd $HOME
gunzip -c beatbox-1.1-417.tar.gz | tar xvf -
cd beatbox-1.1-417
```

and you will be in what we shall call the "beatbox package directory".

## Development versions

Developers and more experienced users may prefer to get the most recent
versions of the package under development. For that,
a Subversion (SVN) client should be working on your
computer. Methods of obtaining SVN are detailed on their web page:

- http://www.sliksvn.com/en/download/

Your flavour of Linux may also support updating the SVN package using
`yum`, `apt-get`, or similar.

Assuming you have
SVN client on, you can create the `beatbox` directory and
download the package into it using the following commands:

```
cd $HOME
mkdir beatbox
cd beatbox
svn co --username=anonymous --password=beatbox https://beatbox-trac.epcc.ed.ac.uk/svn/trunk .
cd beatbox
```

and you will be in the BeatBox package directory.

Note the '.' at the end of the `svn` command. If you are already familiar
with SVN you may note that this will given you a read-only copy of the
directory; if you would like to contribute to the package, you are
welcome to 
contact the authors (see below).

# Compiling and Installing Beatbox

**For the impatient:** the basic commands for compiling on local machines
(including standard MPI clusters) as well as on HECToR (the UK
National Supercomputing facility) have been collected into two bash
shell scripts. To check if your download contains these install
scripts, and that their permissions are execute permissions, use the
following command in the BeatBox package directory:

```
ls -l bbx_compile*sh
```

which should produce something like the following:

```
-rwxrwxr-x. 1 user group 195 Aug  3 17:01 bbx_compile_hector.sh
-rwxrwxr-x. 1 user group  77 Aug 10 17:15 bbx_compile_local.sh
```

Simply running these shell scripts from the BeatBox package directory
will compile and install Beatbox, correspondingly on Hector:

```
./bbx_compile_hector.sh
```

or on a typical ("local") computer:

```
./bbx_compile_local.sh
```

Note that these
scripts will install the executables into `$HOME/bin`;
naturally, for this to work, that directory have to exist.

**Details and variations:**

- Look at the commands in the two bash scripts. The Hector script
  is different in loading the appropriate module, specifying
  explicitly location of the libraries and chosing a different default
  C compiler. Otherwise the two shells do the same taks: configure,
  compile and install. They do not have to be all run every time. Say
  if you only modify one of the source code files, you probably do not
  need to run the configure, just compile and install.
- If you would rather the executables were
  installed elsewhere, you will need to edit the path location in the
  install scripts using the `--prefix` flag which points the
  installation to the directory you specify. Say if you do

  ```
    ./configure --prefix=$HOME/somewhere
  ```

  then the executables will be installed into $HOME/somewhere/bin/
  directory.
- If you modify the code and wish the executables to be suitable
  for debugging, modify the configure command in the following way:

  ```
    ./configure CFLAGS="-g -O0" --prefix=$HOME/somewhere
  ```

  # Checking the Beatbox Installation

  The compile and installation procedure outlined in the previous section will
  produce two executables `Beatbox_SEQ`
  and `Beatbox` in the directory `$HOME/bin`
  (or another directory if you opted for that).
  This can be checked using the command:

  ```
  ls -l $HOME/bin
  ```

  which should give something similar to the following:

  ```
  total 1836
  -rwxr-xr-x. 1 usrname yourgroup  835665 Aug 10 17:23 Beatbox
  -rwxr-xr-x. 1 usrname yourgroup 1036636 Aug 10 17:23 Beatbox_SEQ
  ```

  `Beatbox_SEQ` is the sequential executable,
  and `Beatbox` is the parallel executable. To make the
  binaries accessible from anywhere on your machine, the
  `$HOME/bin` directory should be added to your path.
  Alternatively, you may copy the executables to a directory already
  in your path, or you can call them using their full path name.

  # Running Tests

  Sequential and parallel test scripts are provided to test Beatbox. The
  sequential test scripts are basically for execution on a single
  processor on your local computer. The provided examples of parallel
  test scripts are for execution on HECToR.

  A typical Beatbox simulation is constructed using the `bbs`
  high level scripting language specific to Beatbox. The bulk of
  the `bbs` script represents what should be computed during
  one time step. Other parts of the `bbs` script dictate the
  duration of simulated activity, stimulation protocols, output, and run
  time measurements. Thus, a `bbs` script serves as an input
  for the Beatbox executables `Beatbox_SEQ`
  and `Beatbox`, see examples below.

  On a local machine, usually in the sequential mode,
  the `bbs` scripts can be run directly from the command
  line.

  To help run the `bbs` scripts with variable input
  arguments, or for multiple runs, or in a batch mode on a cluster,
  examples of bash shell scripts are provided.

  ## Sequential Tests

  Examples of sequential scripts are provided in the
  directory `$HOME/beatbox/data/scripts/sequential`.

  ### Understanding Beatbox paradigm and bbs programs with a few examples using Fitz-Hugh-Nagumo cell model

  Here we consider examples of Beatbox `bbs` scripts for 0D
  individual cell simulation, 1D fiber, and 2D sheet of cells simulation
  with Fitz-Hugh-Nagumo (FHN) cell kinetics model. For every simulation
  script, we discuss the inputs, the simulation script, what it does,
  the outputs, and how the outputs could be visualised. To run these
  simulations, go to the Beatbox directory which contains the sequential
  scripts:

  ```
  cd $HOME/beatbox/data/scripts/sequential/FitzHughNagumo_model
  ```

  #### FHN 0D

  The Fitz-Hugh Nagumo cell model Action Potential (AP) is simulated
  using the bbs program `fhn0.bbs`.
  If the `$HOME/bin` directory containing
  the `Beatbox_SEQ` executable is in your path,
  a `bbs` script can be run directly from the command line
  using the command:

  ```
  Beatbox_SEQ fhn0.bbs
  ```

  or you will need to specify the full path to
  the `Beatbox_SEQ` executable otherwise:

  ```
  $HOME/bin/Beatbox_SEQ fhn0.bbs
  ```

  In this example, the sequential Beatbox
  binary, `Beatbox_SEQ` is called with the input of
  the `bbs` script `fhn0.bbs`. Here we give just a
  brief outline of the 0D FHN simulation script, please see the detailed
  comments in the file `fhn0.bbs`.

  - The `fhn0.bbs` script starts with the use
    of `state` device to define the size *xmax\*ymax\*zmax*
    of the medium and the number of dynamic variables *vmax*,
    followed by a series of variable and parameter declarations to prepare
    Beatbox simulation.
  - Then, `k_func` device defines timing variables for feedback
    stimulation to generate an Action Potential.
  - The `euler` device is used to update the ODE model at
    each time step.
  - At a defined instant, the FHN model two dynamic
    variables *u* and *v* are saved by the `record`
    device into the file called `fhn0.rec`, which can be
    visualised using gnuplot.

  The log of the program run is automatically saved into the
  file `fhn0.log` which replicates what you will have
  seen the output produced when you run the sequential code.

  The output file `fhn0.rec` will be used to generate initial
  conditions for the 1D fiber simulation by the `fhn1.bbs`
  script, which is discussed next.

  #### FHN 1D

  The FHN 1D model simulates a propagation of an AP through a 1D fiber
  of connected cells using the `bbs` script
  `fhn1.bbs`.

  The script `fhn1.bbs` makes use of including the parameter
  file `fhn.par`, and uses the 0D output
  file `fhn0.rec` to generate initial conditions for the 1D
  simulation via the phase distribution method.

  Then, the FHN 1D script `fhn1.bbs` can be run as an
  input file for the sequential binary `Beatbox_SEQ` from the
  command line

  ```
  $HOME/bin/Beatbox_SEQ fhn1.bbs
  ```

  The log of the program run will be automatically saved into the file
  `fhn1.log` with the output that will have been produced
  by the execution of the program.
  Here we give just a brief outline of the simulation script, please see
  the detailed comments in the file `fhn1.bbs`.
  - The `fhn1.bbs` script starts with the inclusion of the
    model parameter file `fhn.par`, please see the file for
    details,
  - then calls the `state` device to define the size
    *xmax\*ymax\*zmax* of the medium and the number of dynamic
    variables *vmax* that are going to be used.
  - The simulation protocol is set up using timing parameters for
    `beginning`, `ending`, and pacing.

    Are these equivalent to the simulation start time, end time
    and the output interval? Not sure what pacing is in this context.
  - The output from the 0D `fhn0.bbs` simulation, that is
    the `fhn0.rec` file is used to generate the initial
    conditions for the 1D simulation via the phase distribution method.
  - The `diff` device is used to compute the Laplacian and
    update the Neumann boundary conditions.
  - The `euler` device is used to update the ODE model at each
    time step.
  - Then, the program counts the AP fronts passing a defined point in
    the 1D strand, and after the 5th passing front records the results
    into `fhn1.rec`, which can be visualised using gnuplot.
  The output file `fhn1.rec` will be used to generate initial
  conditions for the 2D sheet of connected cells simulation by the
  `fhn2.bbs` script, which is discussed next.

  #### FHN 2D

  The FHN 2D model is simulated using the bbs
  script `fhn2.bbs`. It can be run at command line using the
  command:

  ```
  $HOME/bin/Beatbox_SEQ fhn2.bbs
  ```

  In this example, the sequential Beatbox
  binary, `Beatbox_SEQ` is called with the bbs
  script `fhn2.bbs`.

  The `fhn2.bbs` program consists of a series of variable and
  parameter declarations, and the use of the `state` device
  to prepare Beatbox for the simulation. Several input parameters are
  conveniently included in a file called `fhn.par`, which is
  included in the bbs program using `<fhn.par>`.

  As before the schedule of the simulation is set up using parameters
  used for `beginning`, `ending`,
  and `pacing` (among other actions) of the simulation. The
  function of the diffusion device `diff` and the ODE
  solver `euler` is explained in the comments.

  Re-entry is initiated using a series of `k_func` devices to
  set the appropriate values of variables to facilitate inducing of
  re-entry in the 2D medium. The protocol for initiating re-entry is by
  means of cross-field stimulation. The actual computation for the
  diffusion is carried out using the `diff` device, while the
  ODE is solved using the `euler` device. Consecutive frames
  from the 2D simulation are stored in a series of ppm files in
  the `ppm/` sub-directory. The ppm files consist of 3 layers
  as a rule, and are given red, green, and blue values. The 2D ppm files
  can be converted to jpegs, tiffs, pngs using
  standard `netpbm` programs (see the Visualisation section
  of the main documentation for details on
  how to do this).

  Here we give just a brief outline of the simulation script, please see
  the detailed comments in the file `fhn2.bbs`.
  - The `fhn2.bbs` script starts with the inclusion of the
    model parameter file `fhn.par`, please see the file for
    details.
  - It then calls the `state` device to define the size
    *xmax\*ymax\*zmax* of the medium and the number of dynamic
    variables *vmax* that are going to be used.- The timing parameters `begin` and `end` are defined
      using `k_func` device
      to be used to for beginning and ending of simulation.- Another instance of the `k_func` device is used to define
        the cross-field stimulation protocol to induce re-entry.- The `diff` device is used to compute the Laplacian and
          update the Neumann boundary conditions.- The `euler` device is used to update the ODE model at each
            time step.- Then, the program outputs a series of `PPM` files consisting
              of periodic snapshots of the simulation.
  The ppm output can be visualised using `netpbm` or any other suitable
  visualisation tool.

  #### FHN 3D

  The FHN 3D box model is simulated using the bbs
  script `fhn3.bbs`. It can be run at command line using the
  command:

  ```
  $HOME/bin/Beatbox_SEQ fhn3.bbs
  ```

  This should work even on small computers. Though note that you will
  need to ensure that the `fhn.par` located
  in `$HOME/beatbox/data/parameters` is in the same directory
  for this to run. If you did not create a `ppm` subdirectory
  for the last example be sure to create it for this example as well
  otherwise it will not run.

  In this example, the sequential Beatbox
  binary, `Beatbox_SEQ` is called with input of the bbs
  script `fhn3.bbs`. The `fhn3.bbs` script
  consists of a series of variable and parameter declarations, and the
  use of `state` device to prepare Beatbox for the
  simulation. Several input parameters are conveniently included in a
  file called `fhn.par`. So you need to
  have `fhn.par` in your working directory. The schedule of
  the simulation is set up using parameters used for beginning, ending,
  and pacing (among other actions) of the simulation. The format,
  function, output of `fhn3.bbs` is exactly the same as in
  the 2D case. After setting up the schedule parameters using
  the `k_func` device, another set of `k_func` are
  used to provide the cross-field stimulus. The `diff` device
  is then used to compute the diffusion of voltage throughout the 3D
  model. The `euler` device then computes the ODE
  (`fhncubpar` in this case) at all points of the finite
  difference grid. The `ppmout` device outputs ppm files at
  each time step. These ppm files are produced in a sub-directory
  called `ppm/` which is presumed to exist in the working
  directory. The ppm output of this `fhn3.bbs` can be
  visualised using `EZView`, a Beatbox related piece of
  software (see below).

  Here we give just a brief outline of the simulation script, please see
  the detailed comments in the file `fhn3.bbs`.
  - The `fhn3.bbs` script starts with the inclusion of the
    model parameter file `fhn.par`, please see the file for
    details.- It then calls the `state` device to define the size
      *xmax\*ymax\*zmax* of the medium and the number of dynamic
      variables *vmax* that are going to be used.- The timing parameters `begin` and `end` are defined
        using `k_func` device
        to be used to for beginning and ending of simulation.- Another instance of the `k_func` device is used to define
          the cross-field stimulation protocol to induce re-entry.- The `diff` device is used to compute the Laplacian and
            update the Neumann boundary conditions.- The `euler` device is used to update the ODE model at each
              time step.- Then, the program outputs a series of `PPM` files consisting
                of periodic snapshots of the simulation.
  The ppm output can be visualised using `netpbm` or any other suitable
  visualisation tool.

  ### Understanding Beatbox paradigm and bbs scripts with biophysically detailed CRN model

  In the previous section, we saw how to run bbs scripts using the
  command line. The bbs scripts themselves were simple in that they did
  not take any inputs. Often in cardiac simulation projects, the same
  program must be executed with a series of parameter values. In this
  section, we will see how to accomplish this using bash scripting. You
  may wish to use Perl or Python instead if you are already familiar
  with those scripting languages.

  We use cell, 1D and 2D models based on the CRN human atrial cell model
  and discuss the inputs, the simulation script, what it does, the
  outputs, and how to visualise/what to do with the outputs. To run
  these simulations, we can go to the following directory in the Beatbox
  installation:

  ```
  cd $HOME/beatbox/data/scripts/sequential/CRN_model
  ```

  #### CRN 0D

  First, we run the CRN cell model to produce a train of action
  potentials. To do this, a bash script command is provided:

  ```
  ./runcrn0.sh
  ```

  The above bash shell script (as with all bash shell scripts) is a text
  file consisting of various commands used to run our program. The above
  bash script contains the command:

  ```
  $HOME/bin/Beatbox_SEQ crn0.bbs
  ```

  This is the most basic of commands that will be used for running a
  Beatbox simulation. However, as of now, lets discuss the function and
  output of `crn0.bbs`. The bbs script stimulates the cell
  model using 10 current pulses. It consists of calling the
  binary `$HOME/bin/Beatbox_SEQ` and passing to it the bbs
  simulation script, in this case
  `crn0.bbs`. This bbs script declares the `state`
  of the simulation, which consists of 1 cell. The periodic
  stimulation, by means of a user defined current, is defined using
  the `k_func` device. Each time step is solved using
  the `euler` ODE solver device. The ASCII output for time
  and voltage is generated using the `sample` device, and put
  into an output file called `crn0.vtg`. The output from
  this bbs script (called `crn0.vtg`, but you can rename it
  to anything you want by editing the `crn0.bbs` file) is a 2
  column record of the time and voltage. This output can be plotted
  using gnuplot,
  or SigmaPlot.
  As you will see below, and in the full manual, arguments can be given
  to the bbs script.

  Here we give just a
  brief outline of the 0D CRN simulation script, please see the detailed
  comments in the file `crn0.bbs`.
  - The `crn0.bbs` script starts with declaring and assigning values to
    several simulation variables like `neqn` (number of equations in the cell
    model), time step `ht` and others.- It then calls the `state` device to define the size
      *xmax\*ymax\*zmax* of the medium and the number of dynamic
      variables *vmax* that are going to be used.- Then, `k_func` device defines timing variables `begin`
        (beginning of simulation), output (often and seldom), `end` (end of simulation),
        and the time `T` in physical units.- Then, another instance of the `k_func` device defines the priodic pacing.- The `euler` device is used to update the ODE model at
            each time step.- The voltage is them sampled using the `sample` device and written to a file
              by the `k_print` device into the file called `crn0.vtg`, which can be
              visualised using gnuplot.

  #### CRN 0D APD restitution

  Often, action potential or Ca2+ transient restitution is required in
  simulation studies. Action potential restitution consists of
  stimulating a cell model with several conditioning stimuli at a give
  basic cycle length, and then a final premature stimulus. The
  diastolic interval and action potential duration of the final
  excitation is recorded and AP features measured. To illustrate how
  action potential restitution can be computed, a bash shell script that
  loops over the premature stimulus interval is provided. To run the
  Beatbox restitution script, use the following bash script:

  ```
  ./restitutioncrn.sh
  ```

  We now discuss the above shell script, as well as the Beatbox bbs
  scripts for restitution that it uses
  called `crn0_rest.bbs`. The above shell script,
  unlike `runcrn0.sh`, introduces several aspects of how
  Beatbox works. Firstly, several simulation variables, like the number
  of conditioning stimuli (`Nstim`), and various file names
  can be defined in the shell script in preparation of passing it to the
  bbs program. Importantly, cell model parameters like `gk1`
  and `gcaL` can also be defined here. Once all the
  parameters are defined, they can be easily passed to the bbs script
  as indicated in the `restitutioncrn.sh` shell script:

  ```
  $HOME/bin/Beatbox_SEQ crn0_rest.bbs $Nstim $Period $fileName $fileName2 $repolarisation $gk1 $gcal
  ```

  Now that the contents of the shell script are clear, let us discuss
  the actual Beatbox bbs script, `crn0_rest.bbs`.

  Firstly, it has been constructed to take several parameters as
  input. The inputs to this specific bbs script are the number of
  conditioning stimuli, the pacing period, the file name for voltage
  trace, the file name for APD data, the amount of repolarisation at
  which restitution is to be computed, and model parameters
  (`gk1` and `gcaL`). Passing of the
  electrophysiological parameters is to show how diseased related to
  electrophysiological can be simulated using Beatbox. The
  program `crn0_rest.bbs` takes all these parameters and sets
  up the simulation.

  The stimulation protocol for the restitution is set up by means of a
  `k_func` device where several pacing parameters are
  defined. Further, the stimulus current amplitude and time profile are
  defined using another call to `k_func` device. It then
  solves the CRN model using the `euler` device. In
  the `.vtg` files, output from voltage, and intra-cellular
  ionic concentration profiles is provided using a combination
  of `sample` and `k_print` devices. Then using a
  combination of `k_func`, `k_print`,
  and `k_poincare` devices, the APD at the user defined
  repolarisation is measured as well as output to file. It is then
  appended to the specified output data file. The output of the above
  simulation can be visualised using gnuplot (or any other plotting
  software). Contents of the vtg files (providing time traces of
  voltage) are visualised using gnuplot as:

  ```
  plot 'crn_basal0.vtg' u 1:2 w l
  ```

  and those of the restitution as (`.apd` file):

  ```
  plot 'crn_basal.apd' u 1:2 w lp
  ```

  Here we give just a brief outline of the 0D CRN restitution simulation script, please see the detailed
  comments in the file `crn0_rest.bbs`.
  - The `crn0_rest.bbs` script starts by assigning the input values to the script
    to variables to be used in the simulation. For e.g. `Nstim` is assigned the second input
    at command line.- The `crn0_rest.bbs` script then declares and assigns values to
      several simulation variables like `neqn` (number of equations in the cell
      model), time step `ht` and others.- It then calls the `state` device to define the size
        *xmax\*ymax\*zmax* of the medium and the number of dynamic
        variables *vmax* that are going to be used.- Then, `k_func` device defines timing variables `begin`
          (beginning of simulation), output (often and seldom), `end` (end of simulation),
          time `T` in physical units, as well as stimulation flags `stim` and
          `penult`.- Then, another instance of the `k_func` device defines the priodic pacing.- The `euler` device is then used to update the ODE model at
              each time step.- The voltage, Ca transient, and other variables are then sampled using the `sample` device and written to a file
                by the `k_print` device into a user defined file which can be
                visualised using gnuplot.- Then the `k_poincare` device is used to measure the APD. The APD measurements are
                  written to file using `k_print` device which can be visualised using gnuplot.

  #### CRN 1D CV restitution

  Now we look at how to construct a 1D strand and initiate propagations
  through the strand. Since we already know a few things about shell
  scripting (see above), we will learn how to conduct conduction
  velocity restitution in a 1D human atrial strand. Conduction velocity
  restitution involves pacing the 1D strand with conditioning pulses (1
  or more) and then applying a premature pulse. The conduction velocity
  of this premature propagating excitation is then measured. To see how
  this is done using Beatbox, use the bash
  script `runcrn1.sh` and the bbs
  script `crn1.bbs`. To simply run the simulation, use the
  bash script command:

  ```
  ./runcrn1.sh
  ```

  The contents of the above script are straightforward. You can set the
  smallest and largest `S2` (premature pulse), as well as the
  output filename and values of a couple of model parameters if
  simulating electrophysiologically diseased CV restitution. In this
  shell script, the simulation actually runs with the command

  ```
  $HOME/bin/Beatbox_SEQ crn1.bbs $S2 $fileName 0.09 0.1235
  ```

  where the Beatbox\_SEQ binary is given the `crn1.bbs` bbs
  script, the value of S2, the output filename, and values of a couple
  of model parameters, in this case `gK1` and
  `gCaL`. The `crn1.bbs` bbs program takes these
  input parameters and starts by setting up the 1D model using
  the `state` device. Several variables and parameters
  required for the simulation are defined using the `k_func`
  device. The actual computation for the diffusion is carried out using
  the `diff` device, while the ODE is solved using
  the `euler` device. A combination
  of `sample`, `k_poincare`,
  and `k_func` devices is used to measure the CV in the 1D
  strand. The measured CV is written to a file with name `crn1.cv`.

  This output can be examined easily as it is an ASCII text
  file. Another output file, with file name specified by the user and
  also in ASCII, is used to record the voltage distribution along the
  strand. If this output file is called say `crn1d500.vtg`,
  then it can be examined using gnuplot using the following commands:

  ```
  set pm3d map
  splot 'crn1d500.vtg' matrix
  ```

  Here we give just a brief outline of the simulation script, please see
  the detailed comments in the file `crn1.bbs`.
  - The `crn1.bbs` script starts with assigning input parameters
    to simulation parameters.- It then calls the `state` device to define the size
      *xmax\*ymax\*zmax* of the medium and the number of dynamic
      variables *vmax* that are going to be used.- The simulation protocol is set up using timing parameters for
        `beginning`, `ending`, and pacing.- The output from the 0D `fhn0.bbs` simulation, that is
          the `fhn0.rec` file is used to generate the initial
          conditions for the 1D simulation via the phase distribution method.- The `diff` device is used to compute the Laplacian and
            update the Neumann boundary conditions.- The `euler` device is used to update the ODE model at each
              time step.- Then, the program computes conduction velocity of consecutive propagations
                using the `sample`, `k_poincare` and `k_func`
                devices.- The CV measurements are put into a `.cv` file using the
                  record device.- The record device outputs the voltage distribution in the strand to a
                    text file, which can be used to make voltage-time plots of the 1D propagation
                    using gnuplot (as shown above).

  ### Phase distribution method for inducing 2D re-entry

  An important part of cardiac simulations is to study the evolution of
  re-entrant waves. Beatbox has a unique method for inducing spiral and
  scroll waves precisely at a desired location in 2D and 3D
  models. Although this method may seem cumbersome at first sight, it
  saves an enormous amount of time and effort once the basics are set
  up. In comparison to the S1-S2 method which requires experimenting
  in case case (e.g. control, AF, HF, ...) to initiate spiral in the
  middle of the sheet, the phase distribution method can be used
  universally once the following cell and 1D records are established.
  The phase distribution method, allows the inducing of re-entry at a
  prescribed location. The details of the method can be found in the
  senior authors publications. The way in which Beatbox can be used to
  implement this method in sequential mode is as follows. The parallel
  variant is discussed in the next section.

  Step 1: Cell model stimulated at a fast rate
  :   First, the cell model is stimulated at a sufficiently rapid pacing
      rate, and all dynamical variables during the last few excitations
      recorded. In the scripts provided, the cell model is encoded in
      `pd_crn0.bbs` and the bash script for running this bbs script is
      `pd_runcrn0.sh`. It is run as:

      ```
      ./pd_crn0.sh
      ```

      Running the above script will show some graphics output on screen at
      run time. Graphics output in sequential mode is another feature of
      Beatbox which allows examination of output visually at run time. The
      above shell script itself is quite simple and calls the Beatbox
      sequential program as:

      ```
      $HOME/bin/Beatbox_SEQ pd_crn0.bbs
      ```

      We now look at the bbs script `pd_crn0.bbs`.

      In this bbs script, relevant parameters and variables are first
      declared and initialised. The model is constructed using
      the `state` device. The phase distribution method requires
      fast pacing of the cell model, which is done by raising the voltage to
      a high value each time it reaches close to its resting potential.
      Such feedback stimulation ensures that all cell model variables are in
      the oscillating state. The graphics window depicts the phase space
      plot of voltage and gating variable `oi` in the CRN model
      on the left, and the successive AP profiles on the right. The
      graphical output is done using a combination
      of `screen`, `clock`,
      `k_clock`, `k_draw` and `update`
      devices. The model is integrated using the `euler`
      device. The output of this simulation is a record of all the model
      variables for several pulses. It will appear as a data file
      named `pd_crn0.rec`.

  Step 2: 1D model stimulated using the 0-D record
  :   This `pd_crn0.rec` file forms input to the 1D strand model
      simulation, which is run as:

      ```
      ./pd_crn1.sh
      ```

      The 1D bbs script `pd_crn1.bbs` starts by setting up all
      the relevant parameters and variables in the simulation, and also the
      sets up the 1D model in Beatbox using the `state`
      device. As in the 0-D simulation above, this bbs program is also set
      up to show graphical output at run time. It shows the propagating
      voltage distribution and the variable `oi` in the CRN model
      as functions of space. The graphical output is generated using a
      combination of `screen`, `clock`,
      `k_clock`, `k_plot` and `update`
      devices. The 1D record from the middle cell of the strand is generated
      using `sample`, `k_poincare`
      and `k_func` devices. It is written to file using the
      `record` device. The output of this 1D simulation is a
      record file called `pd_crn1.rec`.

  Step 3: Phase distribution in the 2D sheet
  :   The record of all dynamical variables from the middle of the strand
      during the final propagating excitation is used to distribute the
      phase of 1 excitation in an Archemedian spiral in a 2D setting, thus
      instantly initiating re-entry. After the 1D simulation executes
      successfully, the 2D simulation is executed using the script:

      ```
      ./pd_crn2.sh
      ```

      This script creates output sub-directories as well as calls the bbs
      program `pd_crn2.bbs` using the sequential Beatbox binary,
      `Beatbox_SEQ`. The contents of `pd_crn2.bbs`
      illustrates several features of the package. In common with all other
      bbs scripts, `pd_crn2.bbs` starts by setting up the
      simulation using variables, parameters, and the `state`
      device. In particular, phase distribution method based initial
      conditions are imposed using the 1D record data to initiate a spiral
      wave at a desired location. The diffusion part of the problem is
      solved using the `diff` device, while the ODE part is
      solved using the `euler` device. Consecutive frames from the 2D
      simulation are stored in a series of ppm files in
      the `ppm/` sub-directory.

      The spiral wave tip at each time step is recorded in the file
      `spiral_tips.dat` using the `singz` device. The
      contents of this file can be visualised using gnuplot. Representative
      AP profiles from various locations in the 2D sheet are provided in the
      output file `pd_crn2.samples`, and the contents can again
      be seen using gnuplot. Frames from the simulation are recorded in
      sequentially numbered PPM files. The ppm files generated by the above
      2D simulation are in a subdirectory called `ppm`. To
      visualise the contents, of say `crn2d_0001.ppm`, we can use
      `netpbm` as follows:

      ```
      ppmtojpeg crn2_0001.ppm crn2_0001.jpg
      ```

      The above command can also be put into a loop using shell scripting,
      or any other suitable method.

  ### Misc

  Each of these runs should exit legally without any error messages. If
  there are error messages, check:

  1. your compilation of the executable worked ok;
  2. that the `$HOME/bin` path is in your environment path (you
     can use `.bashrc` for this if you are using the bash shell);
  3. that you are indeed in
     the `$HOME/beatbox/data/scripts/sequential/` directory.
  To run several of the provided sequential scripts to improve
  confidence in Beatbox using various scripts, use the following bash
  script:

  ```
  ./RunTest.sh
  ```

  ## Parallel Test Scripts

  HECToR is really a subclass of the parallel platforms
  that you could run on - why make it so specific to HECToR?

  To run tests on HECToR, you will need:

  1. the Beatbox parallel binary called `Beatbox`;
  2. a submission script specifically for HECToR;
  3. You have access to HECToR.

  The scripts are not generic enough - for instance
  they assume that whoever is running them will have access to the
  account id e203. I think it would be much better to tell people
  how to run the code as opposed to hiding it all in scripts.

  All the scripts are provided in the directory:

  ```
  $HOME/beatbox/data/scripts/parallel_Hector
  ```

  Further, the submission of the test jobs is facilitated by means of 3
  bash scripts that should be run interactively. To simulate a large 3D
  box using CRN kinetics, just run the bash script:

  ```
  ./bigbox_crn.sh
  ```

  This bash script will:

  1. copy the binary from standard location
     (`$HOME/bin/Beatbox`) to your work space in directories
     called `bigboxcrn_run2` and `bigboxcrn_run2`;
  2. copy the bbs script `bigbox_crn.bbs` to the two newly
     created directories;
  3. produce 2 HECToR job submission scripts;
  4. edit the script line `#PBS -A e203` and specify your
     own valid account against which the execution time will be charged;
  5. submit the parallel jobs to HECToR queues.

  The status of your jobs can be checked using:

  ```
  qstat -u my_user_name
  ```

  Bash scripts for FHN and LRD kinetics are also provided
  (`bigbox_fhn.sh` and `bigbox_lrd.sh`
  respectively).

  The run the 2D phase distribution method based re-entry simulation
  on Hector, you will need the 1D record as generated in the previous section (pd\_crn1.rec).
  In the test examples, the bbs script, the rec file, and the submission assistant script
  are all given in your directory:

  ```
  $HOME/beatbox/data/scripts/parallel_Hector/phasedbn_parallel/
  ```

  Using the submission assistant script given, submit the job to Hector queues as:

  ```
  ./submit_pdcrn2.sh
  ```

  # Visualising 3D ppm output

  Visualisation of the 3D output is by means of the visualisation
  package `EZView`. `EZView` can be obtained from:

  - http://www.maths.liv.ac.uk/~vadim/software/ezview/

  The script for `EZView` is given in the Beatbox distribution:

  ```
  $HOME/beatbox/data/scripts/viz3d/ppmz.task12
  ```

  # Support and feedback

  After working through this quick start guide, you may want to read the
  full manual. A full manual is
  available **HERE**.
  You can get in touch with the authors for support and provide your feedback:

  - Dr Irina Biktasheva
  - Professor Vadim Biktashev
  - Dr Sanjay Kharche

  You can also use this feedback form.

  |  |  |
  | --- | --- |
  | Your name: |  |
  | Your email: |  |
  | Your comments: | |
  |  | |
  |  | |


---

**Copyright © (2010-2012) Vadim Biktashev, Alexander
Karpov,Irina Biktasheva, Ross McFarlane;  
Copyright © 2012Sanjay Kharche  
This file is part of Beatbox.** 
Sanjay.Kharche@liverpool.ac.uk

Last modified: Thu Mar 28 12:26:52 GMT 2013
