# Supplementary material for: BeatBox—HPC simulation environment for biophysically and anatomically realistic cardiac electrophysiology
Source: PLoS One. 2017 May 3;12(5):e0172292. doi: 10.1371/journal.pone.0172292 (PMC5415003; doi:10.1371/journal.pone.0172292)
Supplement: S1 Code — Zip-file S1_code.zip containing distribution of the BeatBox, version beatbox-public-v1.7.982, including source code, configuration and makefiles, documentation, sample scripts etc. (ZIP) [file pone.0172292.s001.zip › beatbox-public-v1.7.982.zip!/beatbox-heart-beatbox-public-05c855e/doc/faq.html]

Beatbox Quick Start Guide


# Beatbox: Archive of User Queries and Solutions

---


## Table of Contents

---


# Mailing list

The Beatbox user mailing list is as follows:

- BEATBOX-USER@jiscmail.ac.uk.

You need to be subscribed in order to be able to post to this list.
Subscription information and a mail archive for this list is available
at:

- https://www.jiscmail.ac.uk/cgi-bin/webadmin?A1=ind1211&L=beatbox-users.

# The queries

## New users Q: Are there practical workshops being conducted where I can bring my specific project, and produce my bbs scripts and set up my scientific project. A: Yes, shortly in the near future. Q: Where can I find information on writing a bbs script? A: Operating system Q: What operating system are supported by Beatbox? A: Beatbox performs best on Red Hat, MAC OS, and Fedora. With suitable configuration, it can also be used in Ubuntu. sample and k\_print Q: I have a 500 x 500 2D sheet with FHN kinetics. I know that I can use the ppm, but I want to sample output at 10,000 points in my simulation, and write it to file using k\_print. Is there a maximum number of sample calls that I can make in a bbs script? A: You can increase the number of devices by altering the `MAXDEV` parameter in `devices.h`. Initial conditions Q: How can I provide initial conditions for my cell model simulation in my bbs script. A: Use the `k_func` device to set your initial conditions at `time=begin`. Clampling a variable to experimental recording Q: How can I clamp my voltage to experimental data. A: Looping Q: How can I loop over several values of pacing cycle length or a parameter value? A: The current method to do this is by means of shell scripting. Looping functionality will be provided in the bbs programming language in the future. HPC systems and local MPI runs fail for no apparent reason, but with SEG fault 11 Q: I am trying to run a bbs script which for no apparent reson fails when run in parallel. The segmentation fault I get is: mpirun noticed that process rank 0 with PID XYZ on node yourcomputer.address.uk exited on signal 11 (Segmentation fault). A: Such errors typically occur when part of your code is trying to access unallocated memory. It may also occur on machines like Hector where the compute nodes do not have all the environment variables as the login nodes. In this case, the first thing that you can do is increase the shell stacksize by means of: ``` ulimit -s unlimited (for bash) ``` or ``` unlimit stacksize (for csh/tcsh) ``` For Mac OS, there is a hard limit on the shell stacksize. For most systems it is: ``` ulimit -s 65531 ``` If this does not help, you should post your bbs script and output of the run (error and output files on Hector) to the user mailing list. --- **Copyright © (2010-2013) Vadim Biktashev, Alexander Karpov,Irina Biktasheva, Ross McFarlane; Copyright © 2012-2013 Sanjay Kharche This file is part of Beatbox.** Sanjay.Kharche@liverpool.ac.uk Last modified: Feb 22 2013
